# mAb therapy controls CNS‐resident lyssavirus infection via a CD4 T cell‐dependent mechanism

**DOI:** 10.15252/emmm.202216394

**Published:** 2023-09-28

**Authors:** Kate E Mastraccio, Celeste Huaman, Si'Ana A Coggins, Caitlyn Clouse, Madeline Rader, Lianying Yan, Pratyusha Mandal, Imran Hussain, Anwar E Ahmed, Trung Ho, Austin Feasley, Bang K Vu, Ina L Smith, Wanda Markotter, Dawn L Weir, Eric D Laing, Christopher C Broder, Brian C Schaefer

**Affiliations:** ^1^ Department of Microbiology and Immunology Uniformed Services University Bethesda MD USA; ^2^ Henry M. Jackson Foundation for the Advancement of Military Medicine, Inc. MD Bethesda USA; ^3^ Department of Preventive Medicine and Biostatistics Uniformed Services University Bethesda MD USA; ^4^ Risk Evaluation and Preparedness Program, Health and Biosecurity CSIRO Black Mountain ACT Australia; ^5^ Centre for Viral Zoonoses, Department of Medical Virology, Faculty of Health Sciences University of Pretoria Pretoria South Africa; ^6^ Centre for Emerging Zoonotic and Parasitic Diseases National Institute for Communicable Diseases, National Health Laboratory Service Pretoria South Africa; ^7^ Present address: Wadsworth Center New York State Department of Health Albany NY USA; ^8^ Present address: Lentigen Technology, Inc. Gaithersburg MD USA; ^9^ Present address: The Center for Bio/Molecular Science and Engineering U.S. Naval Research Laboratory Washington DC USA

**Keywords:** adaptive immunity, Australian bat lyssavirus, Fc function, monoclonal antibody, rabies, Immunology, Microbiology, Virology & Host Pathogen Interaction, Neuroscience

## Abstract

Infections with rabies virus (RABV) and related lyssaviruses are uniformly fatal once virus accesses the central nervous system (CNS) and causes disease signs. Current immunotherapies are thus focused on the early, pre‐symptomatic stage of disease, with the goal of peripheral neutralization of virus to prevent CNS infection. Here, we evaluated the therapeutic efficacy of F11, an anti‐lyssavirus human monoclonal antibody (mAb), on established lyssavirus infections. We show that a single dose of F11 limits viral load in the brain and reverses disease signs following infection with a lethal dose of lyssavirus, even when administered after initiation of robust virus replication in the CNS. Importantly, we found that F11‐dependent neutralization is not sufficient to protect animals from mortality, and a CD4 T cell‐dependent adaptive immune response is required for successful control of infection. F11 significantly changes the spectrum of leukocyte populations in the brain, and the FcRγ‐binding function of F11 contributes to therapeutic efficacy. Thus, mAb therapy can drive potent neutralization‐independent T cell‐mediated effects, even against an established CNS infection by a lethal neurotropic virus.

## Graphical abstract

Rabies is a fatal viral disease of humans, with uniform mortality once central nervous system (CNS) invasion occurs and symptoms appear. This study demonstrates that a single‐dose monoclonal (mAb) therapy can yield a functional cure for rabies, even after robust CNS replication.

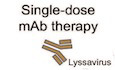

**The paper explained**

**Problem**

Rabies is a disease caused by lyssavirus infection, which is uniformly fatal once the virus invades the central nervous system and symptoms become apparent. Despite the existence of both a highly effective vaccine and post‐exposure prophylaxis, rabies still causes nearly 60,000 deaths a year, predominantly in Africa and Asia. There is no known treatment for symptomatic rabies that can reliably prevent death.

**Results**

In this study, we demonstrated that a peripherally administered single‐dose monoclonal antibody, F11, can efficiently promote the survival of lyssavirus‐infected mice, even after robust viral replication in the CNS. Therapeutic efficacy requires CD4 T cells, but not B cells or CD8 T cells. F11 therapy changes the nature of immune cell infiltration in the brain that is induced by lyssavirus infection, and the therapeutic effect requires functional interaction between F11 and host Fc receptors. Although lyssavirus infection was not completely cleared from the brains of treated animals, disease signs resolved and virus was maintained at a stable low level for at least 4 months post‐infection. Thus, this therapy appears to yield a functional cure for rabies in this animal model.

**Impact**

Although rabies is a very rare disease in industrialized countries, it is still a significant cause of fatality in the developing world, with young people representing a disproportionate number of cases. Despite a formal goal of the World Health Organization of eradicating rabies by 2030, progress to date has been slow, and there thus remains an urgent need for effective therapies for symptomatic rabies. Realistic deployment of impactful therapies for rabies in the developing world will require a treatment that is both cost‐effective and easy to administer in the context of minimally equipped healthcare facilities. Our demonstration that a single dose, peripherally administered monoclonal antibody therapy successfully promotes survival and reverses disease signs in lyssavirus‐infected animals suggests that it may be possible to develop a similar human therapy that meets the above criteria.

## Introduction

Nearly half of all emerging viruses are neurotropic or cause neurological clinical symptoms (Olival & Daszak, 2005). The outlook for human patients with neurotropic viral infections is generally quite grim, even in the developed world, as there are few efficacious therapies, once residence in the CNS is established (Studahl *et al*, 2013; Ludlow *et al*, 2016). Recovery from these infections, when possible, is primarily dependent on successful control of viral infection by the host immune system. Notably, many viruses in this class, including lyssaviruses, have evolved effective mechanisms to evade host immune responses (Lafon, 2011; Studahl *et al*, 2013; Ludlow *et al*, 2016), meaning there is little to no chance of survival upon CNS invasion and concomitant appearance of neurological symptoms. To date, there has been no small molecule‐ or biologic‐based therapeutic approach demonstrated to reliably treat CNS‐resident lyssavirus infection in humans, preventing mortality (Wu *et al*, 2011; Smith *et al*, 2019).

Fifteen of the seventeen recognized lyssavirus species are associated with bat hosts (Davison *et al*, 2017–2020; Markotter & Coertse, 2018). Australian bat lyssavirus (ABLV), an emerging bat‐borne lyssavirus transmitted via a bite or scratch from infected bats (Allworth *et al*, 1996; Hanna *et al*, 2000; Francis *et al*, 2014), has been documented to cause fatal disease in humans and horses. Clinical disease in humans is identical to that of rabies, with ABLV‐infected individuals experiencing symptoms such as ataxia, muscle spasms, difficulty swallowing, paralysis, and encephalitis (Gould *et al*, 1998). Studies elucidating lyssavirus *in vivo* pathogenesis have been primarily based on experimental infections with RABV. In general, lyssaviruses enter the CNS from a peripheral site of inoculation and ascend to the brain via retrograde axonal transport; lyssaviruses then undergo centrifugal spread to various extraneural tissues, facilitating transmission to subsequent hosts (Gillet *et al*, 1986; Charlton *et al*, 1997; Hanlon *et al*, 1999; Dietzschold *et al*, 2008; Velandia‐Romero *et al*, 2013).

Currently, the recommended post‐exposure prophylaxis (PEP) for a suspected exposure to RABV or a rabies‐related lyssavirus prescribes wound cleansing and an immunotherapy approach, consisting of RABV‐vaccination, and, in the case of bite wounds, exposure site injection of polyspecific human or equine rabies immunoglobulin (HRIG or ERIG, respectively) (Manning *et al*, 2008; Both *et al*, 2012; World Health Organization, 2018). The current consensus opinion is that human lyssavirus infection is not treatable once disease signs are apparent (Both *et al*, 2012). Recent studies have shown that administration of attenuated rabies vaccine strains to hamsters previously infected with a lethal RABV strain can protect against mortality. However, efficacy drops off rapidly as the therapy is delivered at increasingly later times post‐infection, with day 4 post‐therapy reported as the latest time point to confer any (22%) survival (Franka *et al*, 2009; Wu *et al*, 2011). Although further development of attenuated and genetically manipulated lyssavirus strains has been suggested as the most promising approach for symptomatic rabies infections (Smith *et al*, 2019), both the potential for emergence of revertants and manufacturing concerns have resulted in resistance to the use of attenuated viruses as vaccines or therapies (Afrough *et al*, 2019).

Monoclonal antibodies (mAbs) represent a highly specific alternative to HRIG. Existing data strongly suggest that HRIG has no therapeutic value once virus enters the CNS, an observation attributed to the likely inability of this therapeutic antibody cocktail to cross the blood–brain barrier (Both *et al*, 2012). A small number of studies have assessed RABV‐neutralizing mAbs in the post‐exposure treatment of rabies. Although these animal studies suggested that neutralizing mAbs may have therapeutic value, administration of mAbs was performed prior to or within 2 h of RABV infection (Schumacher *et al*, 1989; Dietzschold *et al*, 1990, 1992; Prosniak *et al*, 2003; De Benedictis *et al*, 2016), clearly before RABV replication in the CNS. Notably, a recent translational study using mice demonstrated that symptomatic RABV infections in the CNS can be cleared via the administration of two mAbs via simultaneous CNS infusion and peripheral injection (de Melo *et al*, 2020). These data thus suggest that antibody‐based immunotherapy may enable resolution of symptomatic rabies. However, the efficacy of peripherally administered lyssavirus‐neutralizing mAbs following CNS involvement remains poorly defined.

In this study, we employ our recently developed *in vivo* bioluminescence methodology (Mastraccio *et al*, 2020) to assess candidate lyssavirus therapeutics. More specifically, we used longitudinal imaging of lethal‐dose ABLV infection in a mouse model to trace virus location and relative viral load in the presence and absence of therapeutic intervention. We demonstrate that a single dose of human anti‐ABLV/RABV mAb F11 (Weir *et al*, 2021) can limit lyssavirus replication in the CNS, resolve morbidity and promote survival for more than 4 months post‐treatment, even when administered after demonstrable viral replication in the CNS and onset of disease signs. Surprisingly, we found that F11 efficacy requires an intact host adaptive immune response, particularly CD4 T cells, and that immune cell infiltrates in the brains of F11‐treated animals differ significantly from untreated animals. Disruption of F11 FcRγ binding substantially reduces protection against mortality, further demonstrating a role for host immunity in the mechanism of F11 therapeutic efficacy. Together, these observations show that a peripherally administered single dose of F11 induces durable control of lyssavirus infection via a mechanism that requires a functional Fc domain and host CD4 T cells.

## Results

### Control of ABLV‐luc infection by human mAb F11, post‐virus replication in the CNS

**Figure 1:**
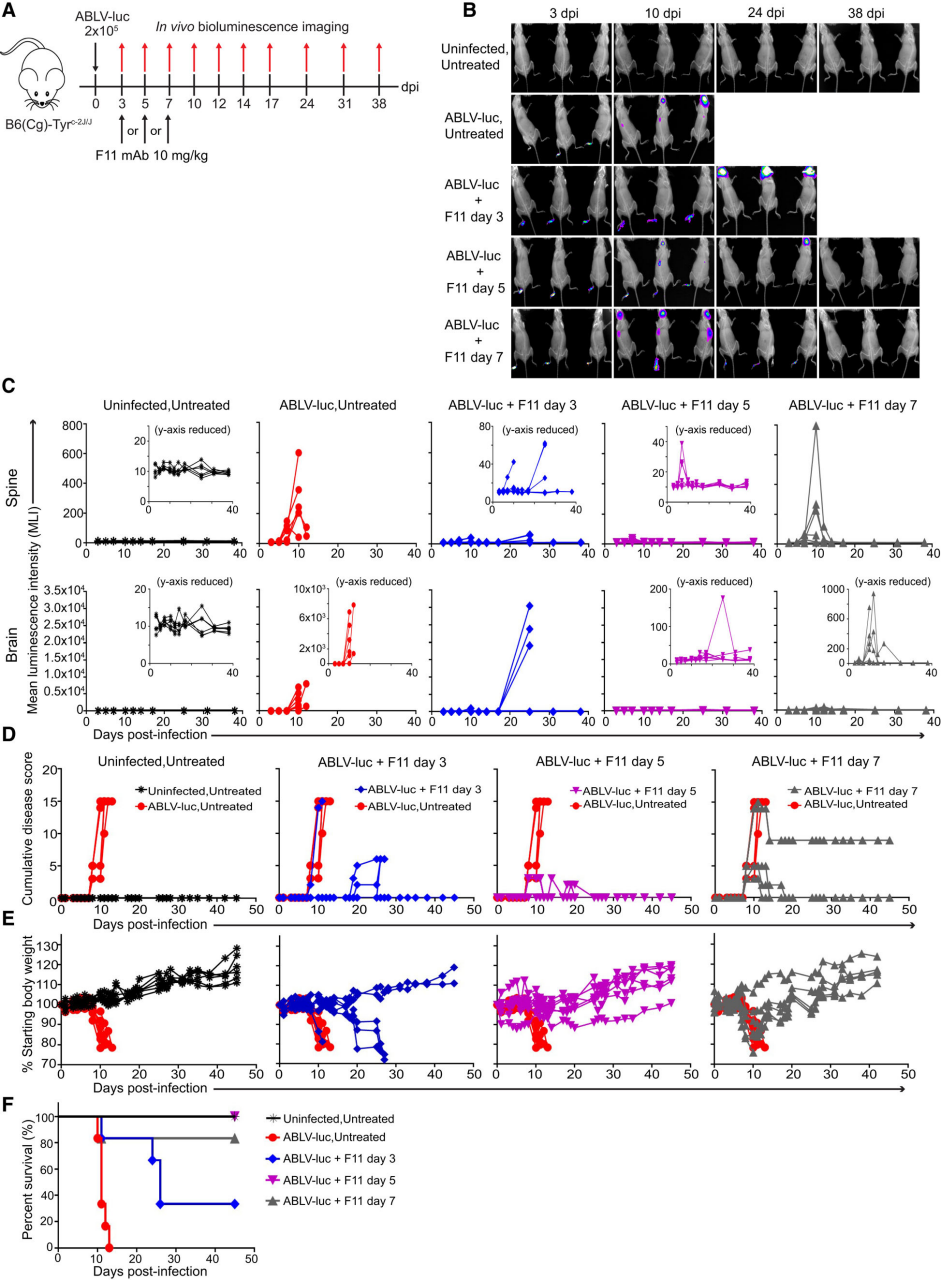
Single dose F11 mAb therapy controls ABLV‐luc infection in the CNS and promotes survival Mice were infected with 2 × 10^5^ FFU of ABLV‐luc on day 0 and mAb F11 (10 mg/kg) was administered on day 3, 5, or 7 (*n* = 6 mice/group).Bioluminescence imaging of mice on days 3, 10, 24, and 38 post‐infection. See Fig EV1D for full series.Viral burden was quantified as mean luminescence intensity in spines (top row) and brains (bottom row) of mice. Insets are same data with reduced y‐axis scale, to show detail.Cumulative disease scores were determined by clinical presentation following ABLV‐luc challenge.Percent starting body weight as an indicator of disease.Kaplan–Meier survival plot. ABLV‐luc Untreated vs. ABLV‐luc F11 day 3, *P* = NS; ABLV‐luc Untreated vs. ABLV‐luc F11 day 5, *P* = 0.0003; ABLV‐luc Untreated vs. ABLV‐luc F11 day 7, *P* = 0.0036. Log‐rank test with Tukey–Kramer correction for multiple comparisons.

**Figure EV1:**
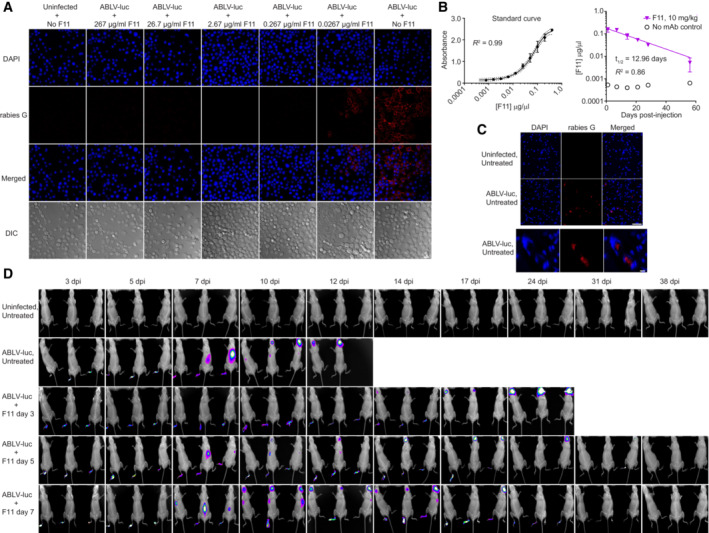
Therapy with F11 mAb controls ABLV‐luc infection in B6 Albino mice Ten‐fold serial dilutions of F11 were incubated with 8 × 10^4^ FFU of ABLV‐luc, added to mouse N2a cells, and cells were stained with anti‐rabies G to detect virions. Bar, 20 μm.Left graph is standard curve of absorbance vs. concentration of F11 diluted in normal mouse serum, measured by ELISA. Right graph is average concentration of F11 in serum of mice over time. Mice were injected on day 0 with 10 mg/kg F11. Sera were collected from 4 mice per time point at 1, 7, 14, 21, 28 and 56 days post injection. Concentration was calculated based on standard curve, and F11 *in vivo* half‐life was calculated via non‐linear regression using one‐phase exponential decay with a least squares fit. Error bars are SD.Coronal sections of brain stem from ABLV‐luc‐infected mice (day 11 post‐infection) were stained to confirm the presence of virus. Bar, top set 50 μm, bottom set 10 μm.Bioluminescence imaging of mice infected with 2 × 10^5^ FFU of ABLV‐luc and treated with F11 on day 3, 5, or 7 post‐infection. Source data are available online for this figure.

F11 is a recombinant human IgG1 mAb that targets the ABLV envelope glycoprotein (G) (see Materials and Methods and Weir *et al*, 2021). As shown in Fig EV1A, F11 demonstrates potent *in vitro* neutralization of ABLV‐luc with a neutralization titer of 0.267 μg/ml. We determined that the *in vivo* half‐life of F11 is approximately 13 days in mice (Fig EV1B), a value consistent with the range determined for other human IgG1 mAbs in rodents (Walker *et al*, 2019). To evaluate the *in vivo* efficacy of mAb therapy on established lyssavirus infection, mice were infected with a lethal dose (2 × 10^5^ FFU) of ABLV‐luc and treated with a single injection of F11 (10 mg/kg body weight) on day 3, 5, or 7 post‐infection (Fig 1A). In addition to bioluminescence imaging, the presence of virus within the CNS was confirmed via immunofluorescence microscopy (Fig EV1C). Treatment initially appeared to prevent CNS infection of day 3‐treated mice, despite ongoing replication in the footpad. However, in these mice, virus appeared in the brain with delayed kinetics (day 14) and was ultimately lethal in 67% of animals by day 26 post‐infection (Figs 1B and C, and EV1D).

While unable to prevent virus from infecting the CNS, treatment with F11 greatly reduced viral burden in the CNS of animals treated at day 5 or 7 post‐infection, as compared to untreated controls. Using bioluminescence imaging, by day 38 post‐infection, we were no longer able to detect virus in the footpad, spine or brain of 100% of mice treated on day 5 and in 83% of mice (100% of surviving mice) treated on day 7 (Figs 1B and C, and EV1D). Similar to untreated animals, mice treated on days 5 and 7 began exhibiting signs of clinical disease on day 8 post‐infection (Fig 1D and E). However, weight loss and clinical disease resolved in all of the mice treated on day 5 and in the majority of mice treated on day 7, by days 25 and 18, respectively. Hind limb paralysis did remain in 33% of day 7‐treated mice despite absence of a luciferase signal (Fig 1D and E).

By contrast, with the exception of one mouse that began exhibiting signs of illness on day 8, clinical disease did not become apparent in day 3‐treated mice until day 19 post‐infection, including rapid weight loss in the days prior to death (Fig 1D and E). Finally, survival analysis showed that treatment of mice with F11 on day 5 or 7 resulted in 100% and 83% survival, respectively, whereas only 33% of mice treated on day 3 survived (Fig 1F). These data demonstrate that F11 therapy is highly effective for treatment of ABLV infections, even when delivered post‐virus replication in the CNS. Moreover, 92% of mice treated on days 5 and 7 that experience severe virus‐induced symptoms ultimately survive, with the majority of these animals showing no long‐term disease signs.

### F11 effectively treats established RABV infection

**Figure 2:**
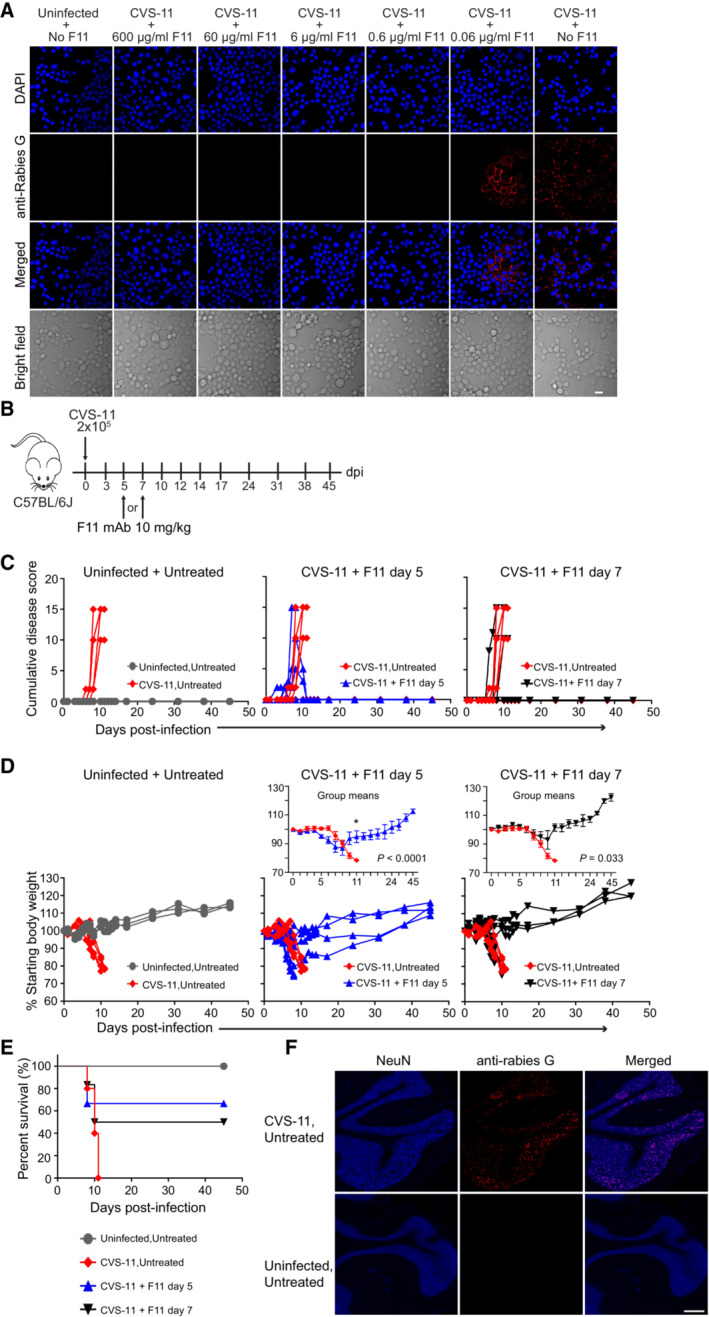
F11 mAb therapy is effective against the lethal rodent‐adapted rabies strain, CVS‐11 A
Ten‐fold serial dilutions of F11 were incubated with 1 × 10^5^ FFU of CVS‐11, added to mouse N2A cells, and cells were stained with anti‐rabies G to detect virions. Bar, 20 μm.B
Mice were infected with 2 × 10^5^ FFU of CVS‐11 on day 0 and mAb F11 (10 mg/kg) was administered on day 5 or 7 (*n* = 3 Uninfected, Untreated mice; *n* = 5 CVS‐11, Untreated; *n* = 6 each: CVS‐11, F11 day 5 and CVS‐11, F11 day 7).C, D
(C) Cumulative disease scores, and (D) Percent starting body weight, over time post‐infection. Insets are means, error bars are SEM. CVS‐11, Untreated vs. CVS‐11, F11 day 5, *P* < 0.0001, CVS‐11, Untreated vs. CVS‐11, F11 day 7, *P* = 0.033. Mixed model with first‐order autoregressive correlation structure. *P = 0.039, on post‐infection day 11 (F11 day 5 treatment group) after Tukey–Kramer adjustment for multiple comparisons.E
Kaplan–Meier survival plot. CVS‐11, Untreated vs. CVS‐11, F11 day 5, *P* = NS; CVS‐11, Untreated vs. CVS‐11, F11 day 7, *P* = NS. Logrank test with Tukey–Kramer correction for multiple comparisons.F
Coronal sections of cerebellum from CVS‐11‐infected mice (day 8 post‐infection) were stained to confirm the presence of virus. Bar, 200 μm. Source data are available online for this figure.

Genetically distinct from other lyssaviruses, ABLV is most closely related to RABV (Horton *et al*, 2010). To assess whether F11 could confer cross‐protection with RABV, mice were infected with 2 × 10^5^ FFU of RABV strain, CVS‐11. As shown in Fig 2A, like ABLV, CVS‐11 is also potently neutralized by F11 *in vitro*, exhibiting a neutralization titer of 0.6 μg/ml. Infected mice were treated with a single dose of F11 on day 5 or day 7 post‐infection and monitored for clinical signs of disease (Fig 2B). Clinical disease and weight loss resolved in 67% of mice treated on day 5 and in 50% of the mice treated on day 7, whereas all untreated mice succumbed to infection between days 8 and 11 post‐infection (Fig 2C and D). Weight loss data were significantly different between both F11 treatment groups and untreated, CVS‐11‐infected animals, although survival data were not (due to modest group sizes and the incomplete protective effect). Notably, those mice that exhibited resolution of clinical signs also survived long‐term (Fig 2E). Importantly, the pathogenic CVS‐11 strain induced clear disease signs as early as day 3 post‐infection, and three out of six mice exhibiting signs on or before day 5 returned to a baseline disease score and survived, post‐F11 treatment. The presence of CVS‐11 within the brains of infected, untreated animals was confirmed via immunofluorescence microscopy (Fig 2F). These data show that F11 is therapeutically effective against the highly virulent rodent‐adapted RABV strain, CVS‐11, demonstrating that efficacy is not unique to ABLV infection. Moreover, these data show that F11 therapy can be effective even after clear onset of disease signs.

### Mice lacking functional adaptive immunity succumb to ABLV‐luc infection despite F11 treatment

As shown in Fig 1, administration of F11 on day 7 post‐infection leads to control of virus replication and survival of a majority of animals, even though ABLV is already robustly replicating within the spine by this time point. However, our data strongly suggest that the mode of virus control is more complicated than simple virus neutralization. Specifically, in animals treated at day 3, we observed rapidly increasing virus replication in the brains of a majority of animals between days 17 and 24 post‐infection (which corresponds to 14 to 21 days post‐F11 administration). Thus, if simple neutralization accounted for the efficacy of F11 therapy, all treatment groups should have exhibited failure to neutralize ABLV by 21 days post‐F11 administration (for the day 5‐ and day 7‐treated animals, this would correspond to days 19–26 and days 21–28 post‐infection, respectively). However, although the majority of day 5‐ and all day 7‐treated mice exhibited readily detectable viral replication in the spine and brain by day 7 post‐infection, with some showing replication as late as day 24 post‐infection, none of these animals exhibited increased viral replication leading to mortality during the 14–21 day period post‐F11 therapy. Thus, the data in Fig 1 do not support virus neutralization as a mechanism sufficient to control ABLV infection in the CNS, conferring protection from virus‐induced mortality.

**Figure 3:**
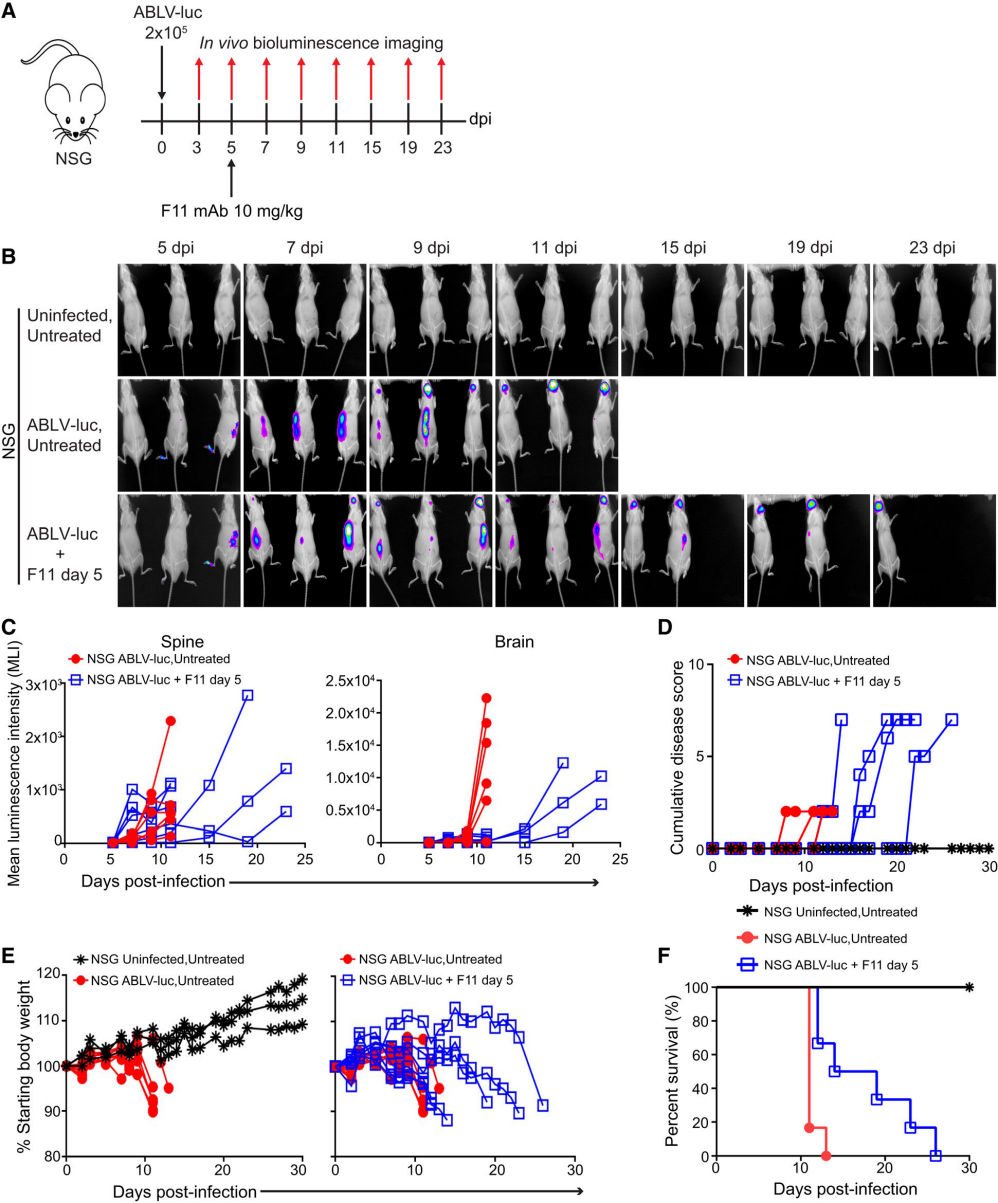
Adaptive immunity is essential for virus control and survival mediated by F11 therapy NSG mice (*n* = 3 Uninfected, Untreated; *n* = 6 ABLV‐luc, Untreated; *n* = 6 ABLV‐luc, F11 day 5) were infected with 2 × 10^5^ FFU of ABLV‐luc on day 0 and mAb F11 (10 mg/kg) was administered on day 5.Bioluminescence imaging of mice following infection.Viral burden was quantified as mean luminescence intensity in the spines and brains.Disease score over time post‐infection.Percent starting body weight.Kaplan–Meier survival plot. ABLV‐luc, Untreated vs. ABLV‐luc, F11 day 5, *P* = 0.005. Logrank test.

**Figure EV2:**
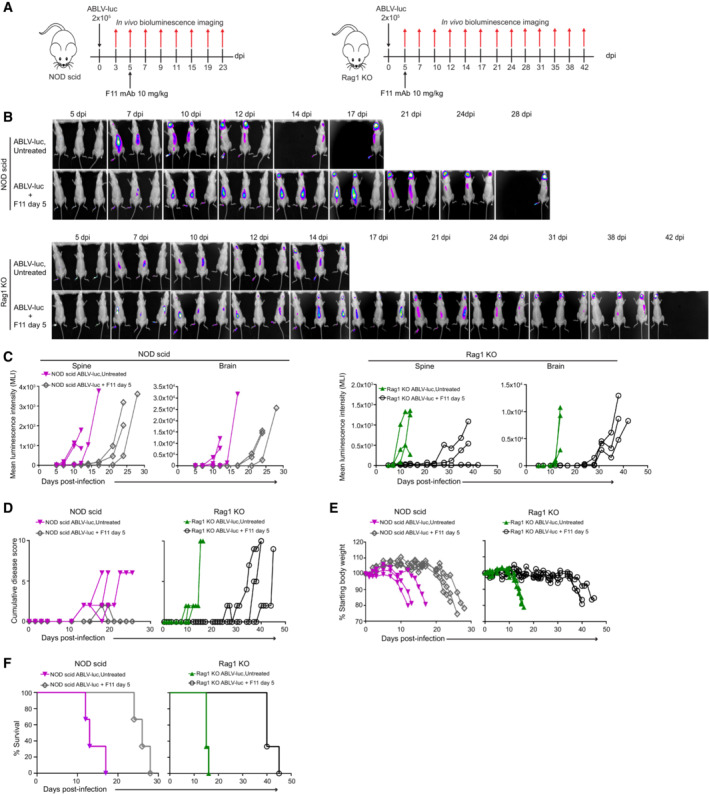
Failure of F11 therapy to promote survival in two additional strains of mice lacking adaptive immune cells NOD‐SCID and Rag 1KO mice were infected with 2 × 10^5^ FFU of ABLV‐luc on day 0 and mAb F11 (10 mg/kg) was administered on day 5 (*n* = 6 mice/group).Bioluminescence imaging of mice was conducted on the indicated days post‐infection.Viral burden was quantified as mean luminescence intensity in spines and brains of mice.Cumulative disease scores were determined by clinical presentation following. ABLV‐luc challenge.Percent starting body weight over time as an indicator of disease.Kaplan–Meier survival plot. NOD‐SCID ABLV‐luc, Untreated vs. NOD‐SCID ABLV‐luc, F11, *P* = 0.025. Rag1 KO ABLV‐luc, Untreated vs. Rag1 KO ABLV‐luc, F11, *P* = 0.022. Logrank test.

Based on the above observations, we hypothesized that F11 facilitates host‐derived cellular and/or humoral immune responses that are responsible for controlling viral infection within the brain. A prediction of this model is that F11 would not be effective at controlling infections in mice lacking an effective adaptive and/or natural killer (NK) cell immune response. To test this hypothesis, we chose the NSG strain, which is deficient in T cells, B cells, and functional NK cells. We infected NSG mice with 2 × 10^5^ FFU of ABLV‐luc and administered F11 on day 5 (Fig 3A). Using bioluminescent imaging, we found that F11‐treated NSG mice were unable to control ABLV‐luc infection in the CNS. Specifically, F11 treatment did not sustain a reduction in viral burden in the brains or spines of NSG mice, although amplification and anatomical progression of the virus was delayed compared to untreated mice (Fig 3B and C). Manifestation of disease signs, weight loss and mortality were also delayed, relative to untreated animals. Ultimately, all F11‐treated NSG mice succumbed to infection by day 26 (Fig 3D–F). Similar results were obtained with NOD‐SCID mice and mice lacking Rag1 expression (Rag1 KO), which lack T and B cells but have functional NK cells (Fig EV2A–F). Taken together, the data in Figs 3 and EV2 further emphasize that simple virus neutralization is not sufficient to protect ABLV‐luc‐infected animals from mortality. Additionally, these data show that an adaptive immune response is essential for controlling ABLV‐luc infection, and that the presence of NK cells is not sufficient to control infection. These data demonstrate that regardless of the presence or absence of adaptive immune cells, F11 potently reduces ABLV‐luc burden in the first several days following *in vivo* administration at day 5 post‐infection. However, while mice with an intact adaptive immune response persistently control this infection, mice lacking adaptive immune cells exhibit rapid expansion of ABLV‐luc during approximately weeks 2–6 post‐infection. Thus, these data strongly suggest that facilitation of an antiviral adaptive immune response is necessary for F11‐dependent long‐term control of lyssavirus infection.

### B cells are dispensable for ABLV control

One possible mechanism by which F11 might control ABLV‐luc infection is simply via delaying robust virus replication for long enough that the host humoral (B cell) immune response is able to produce high amounts of neutralizing antibody, ultimately controlling infection. We thus assessed the requirement for host B cells in control of ABLV‐luc infection. To accomplish this, B cell‐deficient mice (muMt^−^) were infected with 2 × 10^5^ FFU of ABLV‐luc and treated with F11 on day 5. Control of ABLV infection and resolution of clinical disease was observed in all muMt^−^ mice (Appendix Fig S1A–E). Similar to WT mice, all F11‐treated muMt^−^ mice survived and exhibited minimal changes in body weight (Appendix Fig S1B and F). Thus, these data demonstrate that host B cells do not measurably contribute to the therapeutic efficacy of F11.

### CD4 T cells but not CD8 T cells are required for F11‐mediated survival following ABLV‐luc infection

Because B cell‐deficient mice showed no reduction in F11 efficacy in comparison to WT mice, we concluded that the key effector population must be a T cell subset. We first addressed the role of CD8 T cells, as the primary function of this T cell subset is the killing of cells infected with intracellular pathogens, such as viruses. Although we initially explored the use of a genetic model in which mice fail to differentiate the CD8 T cell lineage, the class I‐deficient (β2‐microglobulin^−/−^) model could not be used, because this model also lacks functional FcRn, which is required for IgG transport to extracellular spaces and which greatly extends the *in vivo* half‐life of IgG (Ghetie *et al*, 1996; Junghans & Anderson, 1996; Akilesh *et al*, 2007; Roopenian & Akilesh, 2007). The CD8^−/−^ model is also not appropriate for these studies, as it has been shown that these mice still produce functional cytotoxic cells in quantities sufficient to clear certain viral infections (Dalloul *et al*, 1996; Andrews *et al*, 2008).

**Figure 4:**
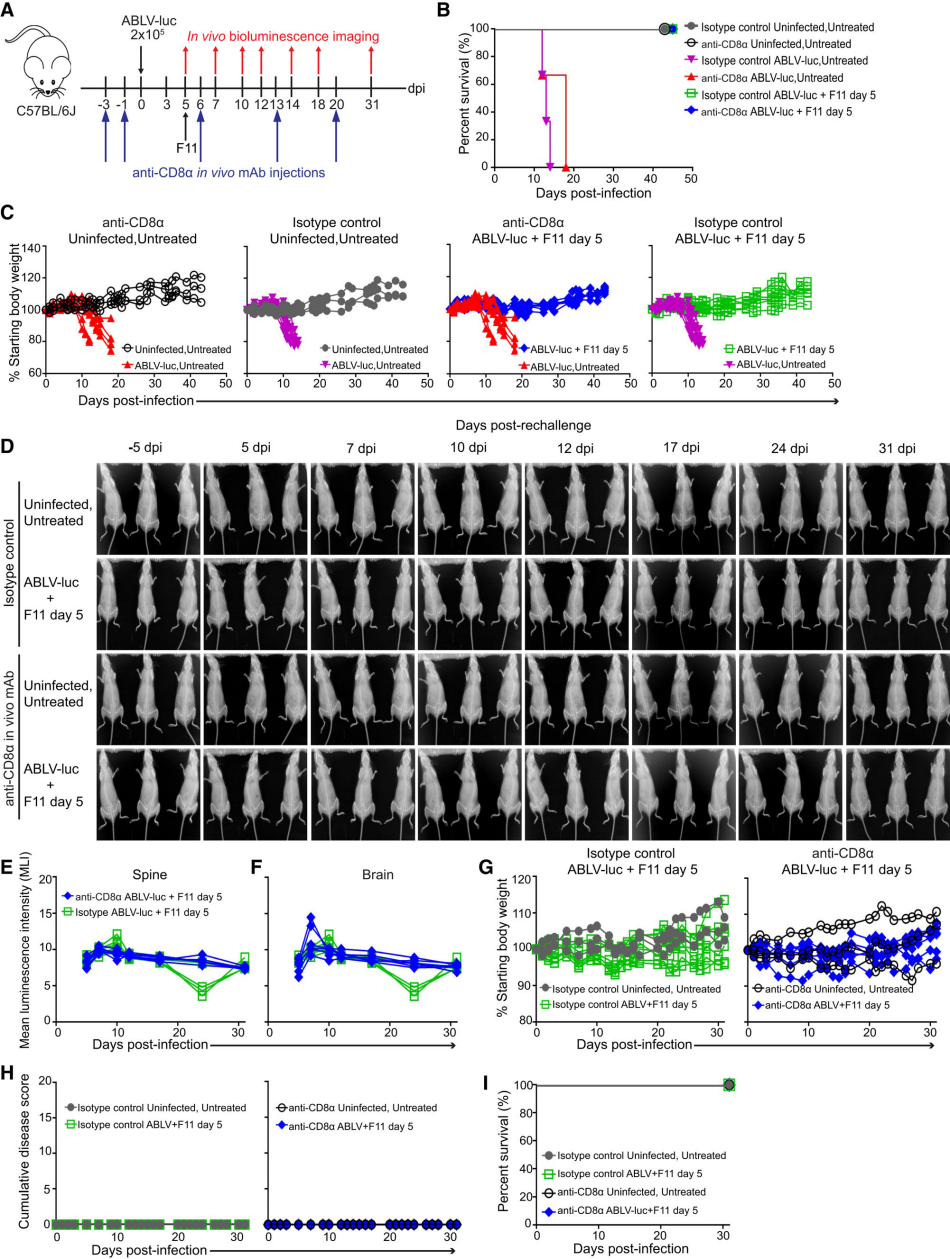
CD8 T cells are dispensable for F11‐depenent control of viral infection A
Isotype control and CD8 T cell‐depleted C57BL/6J mice were infected with 2 × 10^5^ FFU of ABLV‐luc on day 0 and mAb F11 (10 mg/kg) was administered on day 5 (*n* = 6 mice/group, *n* = 3 Uninfected, Untreated mice/group).B
Kaplan–Meier survival plot. Isotype, ABLV‐luc, Untreated vs. isotype, ABLV‐luc, F11, *P* = 0.0006; anti‐CD8α, ABLV‐luc, Untreated vs. anti‐CD8α, ABLV‐luc, F11, *P* = 0.0197; isotype, ABLV‐luc, Untreated vs. anti‐CD8α, ABLV‐luc, Untreated = NS. Logrank test with Tukey–Kramer correction for multiple comparisons.C
Percent starting body weight over time post‐infection. over time post‐infection.D
Bioluminescence imaging of mice on days −5, 5, 7, 10, 12, 17, 24, and 31 post‐rechallenge.E, F
Viral burden was quantified as mean luminescence intensity (MLI) in the spines (E) and brains (F) of rechallenged mice.G–I
Percent starting body weight (G), cumulative disease scores (H), and Kaplan–Meier survival plot (I) of rechallenged mice.

**Figure EV3:**
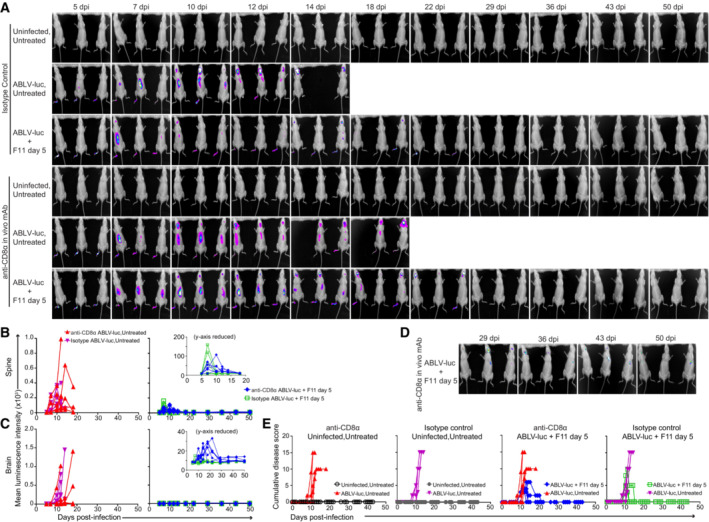
CD8 T cells do not play an essential role in F11‐dependent control of viral infection A
Bioluminescence imaging of isotype control and CD8 T cell‐depleted mice infected with 2 × 10^5^ FFU of ABLV‐luc and treated with mAb F11 on day 5 (*n* = 6 mice/group, *n* = 3 Uninfected, Untreated mice/group).B, C
Viral burden was quantified as mean luminescence intensity (MLI) in the spines (B) and brains (C) of infected mice. Insets are same data with a reduced y‐axis scale; note that inset y‐axis values are not multiplied by 10^3^.D
Bioluminescence imaging of CD8 T cell‐depleted mice maintaining low levels of virus within the brain (same data as (A), but with reduced intensity scale to enable visualization of low intensity luminescence).E
Cumulative disease scores were determined by clinical presentation following ABLV‐luc challenge.

Having ruled out a genetic approach, we instead used *in vivo* mAb‐depletion of CD8 T cells to determine whether CD8 T cells were required for control of viral infection. Preliminary studies confirmed an appropriate dose and timeline of anti‐CD8α mAb administration for ≥ 95% CD8 T cell depletion in mice through at least day 20 (Appendix Fig S2). Mice were infected with 2 × 10^5^ FFU of ABLV‐luc and treated with F11 on day 5 (Fig 4A). While elimination of a detectable bioluminescent signal in the brain was delayed in CD8 T cell‐depleted mice relative to mice given isotype control mAb (Fig EV3A–C), all F11‐treated mice survived (Fig 4B). Interestingly, very low levels of viral replication could still be detected in the spines and brains of CD8 T cell‐depleted mice through day 50 post‐infection (Fig EV3B–D; beyond this time point, no animals exhibited detectable viral replication), which is substantially longer than observed in WT mice. Thus, it seems likely that CD8 T cells may facilitate elimination of virus‐infected cells. However, as no differences in body weight were observed between CD8 T cell‐depleted mice and control mice (Fig 4C), and resolution of clinical disease was observed in all CD8 T cell‐depleted and control animals (Fig EV3E), these data strongly suggest that CD8 T cells are dispensable for F11‐mediated control of lyssavirus infection and protection from mortality.

To determine if mice that recovered from ABLV‐luc infection developed protective immunity, we rechallenged day 5‐treated mice on day 82 post‐infection with 2 × 10^5^ FFU of ABLV‐luc. Upon rechallenge, virus could not be detected by bioluminescence imaging in any tissue, including the inoculated footpad, in isotype control or in CD8 T cell‐depleted mice. No mice exhibited clinical disease (Fig 4D–H), and 100% of isotype control and CD8 T cell‐depleted mice survived (Fig 4I). Together, these data demonstrate that F11‐treated mice develop a protective immune response capable of preventing infection upon re‐challenge with a lethal dose of ABLV‐luc. Although CD8 T cells may contribute to control of virus infection, our data suggest this population is not essential for F11‐dependent survival post‐lyssavirus challenge. Additionally, these data may suggest that CD8 T cells are not essential for development of a protective memory response. However, given that we employed antibody depletion of CD8 T cells rather than a strain that completely lacks this subset, we cannot rule out that small numbers of memory CD8 T cells were produced during the primary infection, and that these CD8 memory cells play an essential role in the response to secondary challenge.

**Figure 5:**
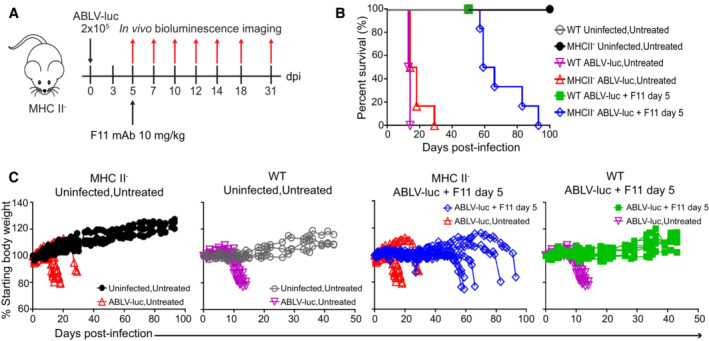
CD4 T cells are essential for control of viral infection and F11‐dependent survival C57BL/6J and MHCII^−^ mice were infected with 2 × 10^5^ FFU of ABLV‐luc on day 0 and mAb F11 (10 mg/kg) was administered on day 5 (*n* = 6 mice/group, except *n* = 3 Uninfected, Untreated mice).Kaplan–Meier survival plot. WT, ABLV‐luc Untreated vs. WT, ABLV‐luc F11, *P* = 0.0004; MHCII^−^, ABLV‐luc Untreated vs. MHCII^−^, ABLV‐luc, F11, *P* = NS; WT, ABLV‐luc, F11 vs. MHCII^−^, ABLV‐luc, F11, *P* = NS; WT, ABLV‐luc Untreated vs. MHCII^−^, ABLV‐luc, Untreated, *P* = NS. Logrank test with Tukey–Kramer correction for multiple comparisons.Percent starting body weight over time post‐infection.

**Figure EV4:**
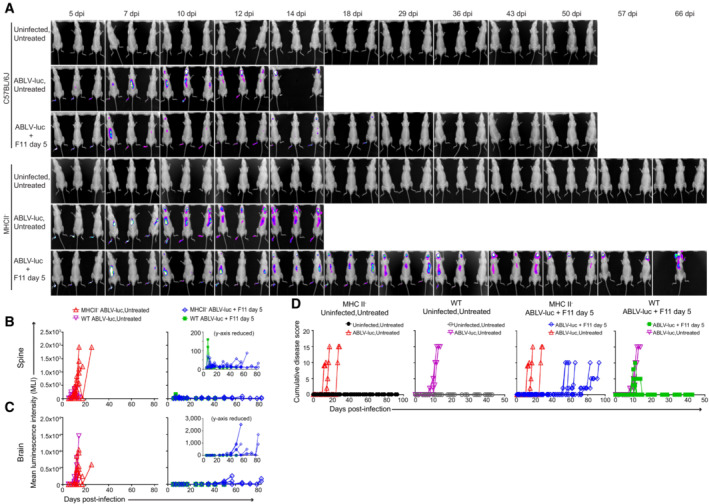
CD4 T cells are essential for F11‐dependent control of viral infection within the CNS A
Bioluminescence imaging of C57BL/6J and MHCII‐mice infected with 2 × 10^5^ FFU of ABLV‐luc and treated with mAb F11 on day 5 (*n* = 6 mice/group, except *n* = 3 Uninfected, Untreated mice).B, C
Viral burden was quantified as mean luminescence intensity (MLI) in the spines (B) and brains (C) of infected mice. Insets are same data with reduced y‐axis scale.D
Cumulative disease scores were determined by clinical presentation following ABLV‐luc challenge.

**Figure EV5:**
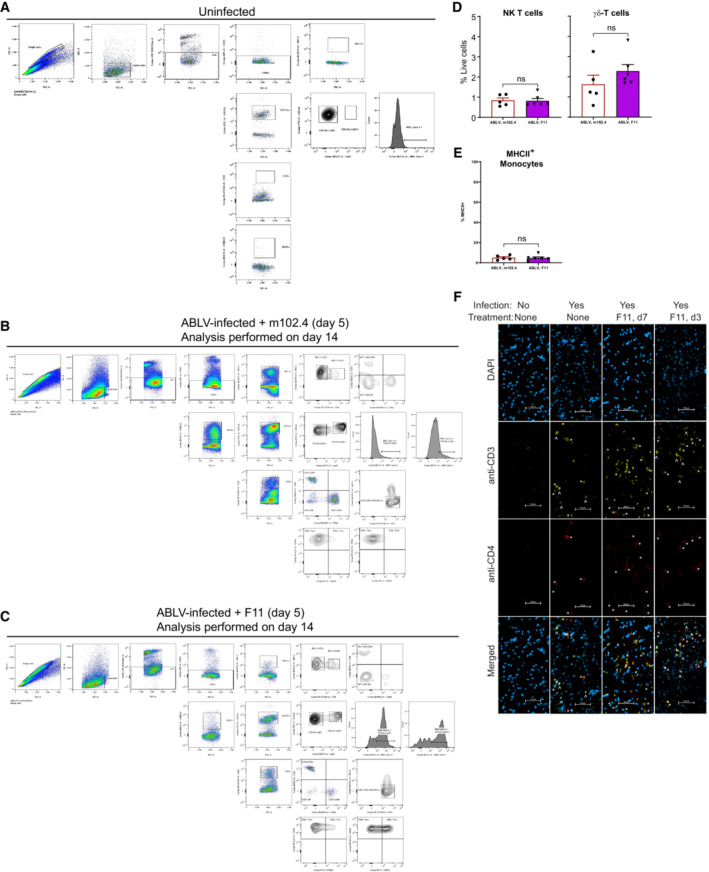
Flow cytometry and microscopy analyses of leukocyte populations in brains of ABLV‐luc‐infected mice A–C
Flow cytometry gating strategy for identification of brain leukocyte populations in mice that were (A) uninfected, (B) ABLV‐luc infected, treated with m102.4 on day 5 and euthanized on day 14 post‐infection, or (C) ABLV‐luc infected, treated with F11 on day 5 and euthanized on day 14 post‐infection.D
Animals were treated with m102.4 (*n* = 5) or F11 (*n* = 6), and cells were prepared from brains as described in Fig 6A and Materials and Methods. Mean percentages of NK T cells (left) and γδ T cells (right), *P* = NS for each population by unpaired *t*‐test and Mann–Whitney test, respectively. Error bars are SEM.E
Animals were treated with m102.4 (*n* = 5) or F11 (*n* = 6), and cells were prepared from brains as described in Fig 6A and Materials and Methods. Mean percentages of MHCII^+^ monocytes, *P* = NS by unpaired *t*‐test. Error bars are SEM.F
Hindbrains from mice of the indicated treatment groups were fixed and sectioned for histology, followed by staining with DAPI, anti‐CD3 and anti‐CD4. Examples of typical staining patterns are shown. *indicates cells positive for CD3 and CD4 (CD4‐pos), ^indicated cells positive for CD3 only (CD4‐neg). Bar, 50 μm. Source data are available online for this figure.

To investigate the contribution of CD4 T cells in control of viral infection, we infected CD4 T cell‐deficient mice (MHCII^−^) with 2 × 10^5^ FFU of ABLV‐luc. Mice were treated with F11 on day 5 (Fig 5A). Through bioluminescence imaging, we found that MHCII^−^ mice are unable to control infection with ABLV‐luc, exhibiting a persistent bioluminescent signal in the spinal cord and brain (Fig EV4A–C), which was substantially higher than the very weak bioluminescence observed in the CD8‐depleted animals. Although the kinetics of virus amplification to levels predictive of mortality in the CNS were delayed in F11‐treated MHCII^−^ mice (Fig EV4B and C), these animals ultimately exhibited high levels of virus replication in the brain in conjunction with weight loss and evolution of clinical disease signs. Indeed, 100% of MHCII^−^ animals reached euthanasia criteria between days 55 and 90 post‐infection (Fig 5B and C, and EV4D). Together, the data in Figs 3, EV2, 4, EV3, 5 demonstrate that F11‐mediated control of lyssavirus infection and prevention of lyssavirus‐mediated mortality requires an adaptive immune response, with CD4 T cells being the lymphocyte subset that is specifically required for this activity.

### A CD4 T cell‐biased brain infiltrate is indicative of a therapeutic response to F11

The simplest explanation for the above genetic data is that T cells infiltrate the brain of F11‐treated, lyssavirus infected mice to facilitate control of ABLV‐luc infection, with CD4 T cell playing a crucial role. Although normally excluded from the brain by the blood–brain barrier (BBB), cells of the immune system can be efficiently recruited to the brain in specific circumstances, including viral infection (Engelhardt, 2006; Arima *et al*, 2013). To determine whether F11 therapy changes the spectrum of leukocytes within the infected brain, we used flow cytometry to compare leukocyte subtypes in infected animals treated with either F11 or with the anti‐henipavirus G glycoprotein huIgG1 mAb, m102.4 (Zhu *et al*, 2008) (which has an identical constant region to F11, but has no measurable interaction with ABLV G (Weir *et al*, 2021)). Animals were infected with 2 × 10^5^ FFU of ABLV‐luc and treated with either F11 or with m102.4 at day 5 post‐infection. Animals were euthanized at day 14 or 15 post‐infection, and brains were removed and processed to single‐cell suspensions. Leukocytes were then purified, stained and analyzed by flow cytometry.

**Figure 6:**
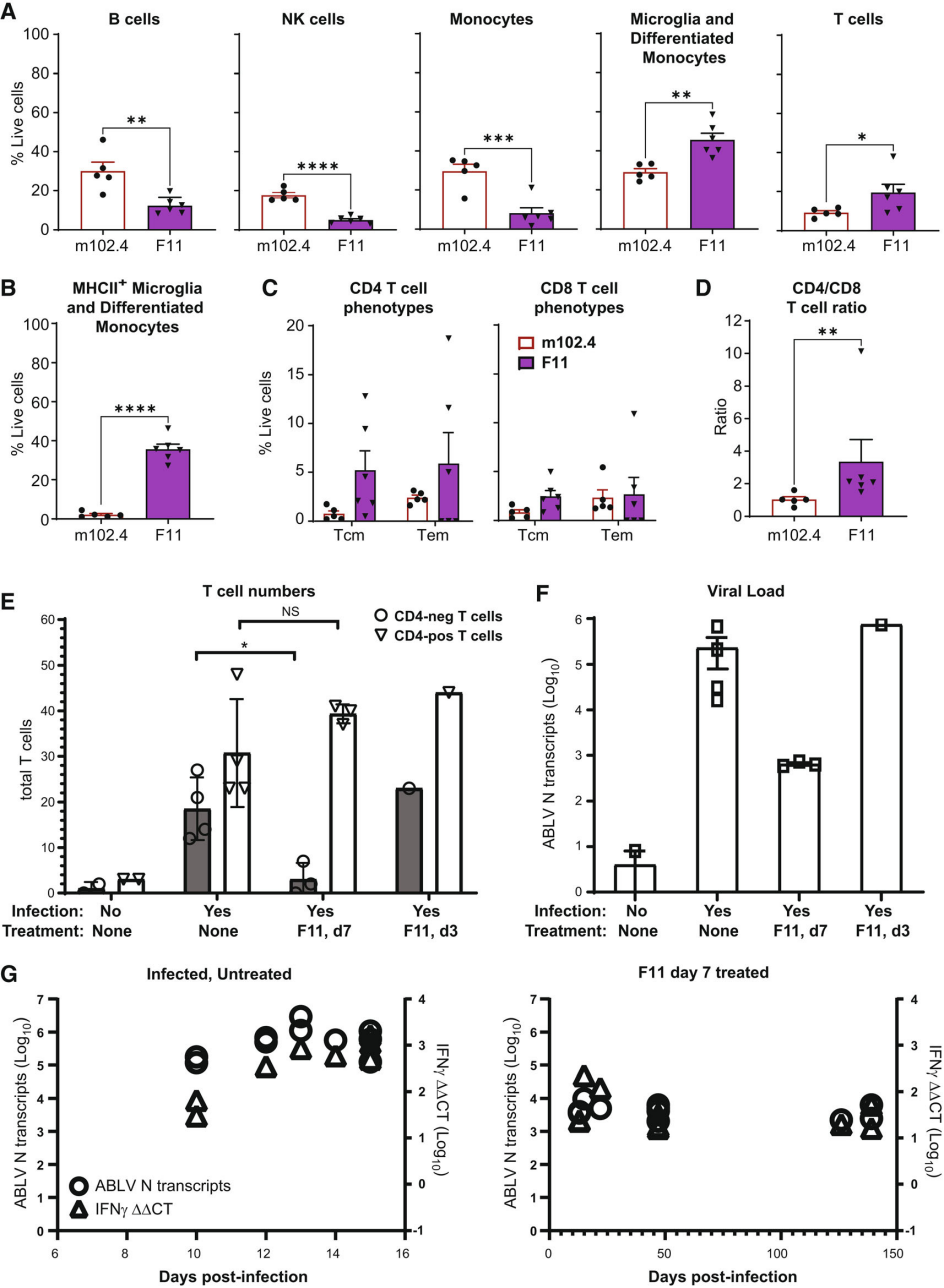
F11 treatment of ABLV‐infected aninals leads to a CD4‐dominant T cell response and chronic, low‐level viral persistence in the brain B6 albino mice were infected with 2 × 10^5^ FFU of ABLV‐luc on day 0 and treated with m102.4 (*n* = 5) or F11 (*n* = 6) (both at 10 mg/kg) on day 5 post‐infection. Brains were harvested at day 14 except for one brain from an F11‐treated animal harvested on day 15 post‐infection. Animals were injected with a labeled anti‐CD45 antibody just prior to euthanasia to enable exclusion of circulating leukocytes in downstream analyses. Leukocytes were purified from each brain followed by staining, fixation, and flow cytometry analysis, as detailed in Materials and Methods. Graphs show mean percentages of each population. Unpaired *t*‐test results of m102.4 vs. F11 groups as follows: B cells, **P = 0.0038; NK cells, ****P < 0.0001; monocytes, ***P = 0.0010; microglia/differentiated monocytes, **P = 0.0029. Mann–Whitney test: T cells, *P = 0.0303.Mean percentages of MHCII^+^ microglia/differentiated monocytes. Unpaired *t*‐test, ****P < 0.0001.Mean percentages of Tcm and Tem populations in CD4 T cells (left) and CD8 T cells (right). All means in (A–C) represent percent of live cells not labeled with the CD45 exclusionary marker.Ratio of CD4 T cells to CD8 T cells in each group. Mann–Whitney test: T cells, **P = 0.0087.B6 albino mice were uninfected or infected with 2 × 10^5^ FFU of ABLV‐luc on day 0. Some mice were treated on day 3 or day 7 post‐infection with mAb F11 (10 mg/kg). Brains were harvested at the peak of acute infection in untreated animals (day 13–15), except for the day 3 F11‐treated animal, which was euthanized on day 22, due to reaching euthanasia criteria. Sections of the hindbrain were stained to detect CD3 and CD4, and total numbers of CD4‐pos T cells and CD4‐neg T cells were quantified for each animal (see Materials and Methods). Numbers of CD4‐pos T cells in infected, untreated vs. infected, F11 treated day 7, *P* = NS. Numbers of CD4‐neg T cells in infected, untreated vs. infected, F11 treated day 7, *P = 0.0135. Unpaired *t* test with Welch's correction.qRT‐PCR was used to quantify ABLV‐N mRNAs in brains of the same animals in (E), as a proxy of viral load. ABLV‐N mRNAs were quantified as absolute numbers of transcripts per μg of brain RNA.qRT‐PCR was used to quantify ABLV‐N (left y‐axis) and IFNγ (right y‐axis) mRNAs in brains of individual animals euthanized at the indicated times post‐ABLV infection. ABLV‐N mRNAs were quantified as in (F), and IFNγ mRNAs were quantified by the ΔΔCT method, expressed as fold‐increase relative to uninfected control animals. Animals were ABLV‐infected and untreated (left) or F11‐treated, day 7 (right). Data information: Error bars are SEM for all panels.

As expected, this analysis demonstrated that with the exception of CD11b‐positive cells (predominantly CD11b^+^ Ly5C^−^ resident microglia), leukocytes are very rare in uninfected animals (Fig EV5A). By contrast, infected animals had readily detectable populations of B cells and NK cells, CD11b^+^ Ly5C^+^ monocytes and T cells, in addition to microglia (and differentiated monocytes, which have the same CD11b^+^ Ly5C^−^ phenotype) (Fig EV5B and C). Importantly, F11 therapy induces significant changes in the proportions of these leukocyte populations in comparison to animals treated with the irrelevant control mAb, m102.4: B cells, NK cells and CD11b^+^ Ly5C^+^ monocytes are present in lower proportions while T cells and CD11b^+^ Ly5C^–^ differentiated monocytes/microglia are present in higher proportions in the brains of F11 treated animals (Fig 6A). NK T cells and γδ T cells were present in small percentages that did not differ between F11 and m102.4‐treated animals (Fig EV5D). Interestingly, while CD11b^+^ Ly5C^+^ MHCII^+^ monocytes were present in equal proportions, CD11b^+^ Ly5C^–^ MHCII^+^ differentiated monocytes/microglia were 18‐fold more abundant in mice treated with F11 as compared to the m102.4 group (Fig 6B). As anticipated, all T cells in the brains of infected animals had effector‐memory phenotypes (Fig EV5B and C), and there were no notable distinctions between Tcm and Tem phenotypes for CD4 T cells or CD8 T cells with either mAb treatment (Fig 6C). However, CD4 T cells were both present at a higher proportion and a higher ratio in relation to CD8 T cells in the brains of animals treated with F11 as compared to the m102.4 group (Fig 6C and D). Thus, flow cytometry data demonstrate that F11 therapy induces a shift in leukocyte populations that infiltrate the lyssavirus‐infected brain, including a significant increase in abundance of MHCII^+^ differentiated monocytes/microglia and CD4 T cells relative to CD8 T cells.

To confirm the flow cytometry analysis of the shift in the CD4/CD8 T cell ratio in the brains of F11‐treated animals, we next performed a microscopy‐based analysis. For these experiments, B6 albino mice were infected with 2 × 10^5^ FFU of ABLV‐luc and treated with F11 on day 7. Because our preferred method of preparing brains for immunofluorescence microscopy involves fixation in paraformaldehyde, and because we were unable to identify an anti‐mouse CD8 mAb that stains paraformaldehyde‐fixed tissues, we were not able to stain CD8 T cells directly. Thus, we used a staining strategy in which brains were stained with anti‐CD3 and anti‐CD4. By this method, double‐staining cells are identified as CD4 T cells (“CD4‐pos”), and cells staining with anti‐CD3 only are identified as CD8 T cells, γδ‐T cells or CD4‐NK T cells (“CD4‐neg”; based on flow cytometry data, the majority of these are CD8 T cells). Microscopy analysis focused on the cerebellum and neighboring regions of the hindbrain, where bioluminescent signals indicative of ABLV‐luc infection were consistently most abundant (see above). While CD4‐pos and CD4‐neg T cells were rare within the brains of uninfected controls, both subsets were readily detected in the brains of infected, untreated mice. Interestingly, although CD4‐pos T cells were similarly abundant in the brains of infected, F11‐treated mice, the CD4‐neg subset was significantly less frequent in the animals treated with F11 on day 7 (Figs 6E and EV5F; Table 1).

**Table 1:** Summary of histological data and viral RNA copies.

Mouse ID	Days post‐infection	Number of cells		ABLV N RNA^a^	Disease signs
		CD4^+^ and CD3^+^	CD3^+^ only		
Uninfected, Untreated					
UU1A	15	3	0	8	N/A
UU3A	15	3	2	0	N/A
Infected, Untreated					
IU0A	15	29	14	16,290	Yes
IU2A	13	48	21	670,845	Yes
IU3A	13	23	27	209,948	Yes
IU4A	15	23	12	30,932	Yes
Infected, F11 day 7					
I7F0B	13	41	0	626	No
I7F1A	15	40	2	730	No
I7F2A	13	37	7	585	No
Infected, F11 day 3					
I3F0A	22	44	23	750,059	Yes

N/A, not applicable.

^a^ Viral RNAs are copies/μg of total RNA purified from a brain hemisphere at time of euthanasia.

To rigorously determine viral burden in these same animals, we developed a quantitative real‐time PCR (qRT‐PCR) assay to measure viral mRNA encoding the nucleoprotein (N), since N is the most abundant mRNA produced by transcription of the lyssavirus genome (Albertini *et al*, 2011). Notably, when compared to the infected, untreated group, the day 7‐treated animals had low levels of viral transcripts in the brain (2‐ to 3‐logs lower), showing that they were responding to F11 therapy (Fig 6F; Table 1), consistent with luciferase imaging data in similar experiments (e.g., Fig 1). We also performed staining of T cells in the brain of a day 3‐treated animal in which disease signs were apparent, indicative of a failure of F11 therapy (see Fig 1). Similar to infected, untreated animals, we observed high levels of both the CD4‐pos and CD4‐neg subsets in the brain of this animal (Figs 6E and EV5F; Table 1), as well as the expected high viral burden (Fig 6F, Table 1). In combination with the flow cytometry data, these microscopy results show that in lyssavirus‐infected animals in which F11 therapy is not given or is not successful, the infected brains exhibit infiltration of CD4 and CD8 T cells in similar proportions, with a high viral burden. By contrast, when F11 therapy is delivered and is efficacious, animals exhibit enhanced infiltration of a T cells that are predominantly CD4^+^ and a large reduction in viral burden. Overall, the combined flow cytometry and histological findings are consistent with the data of Figs 3, EV2, 4, EV3, 5, implicating CD4 T cells as a population required for long‐term F11‐dependent control of lyssavirus infection.

### Persistent low‐level ABLV‐luc infection and expression of cellular immune response genes in the brain following F11 therapy

Although our bioluminescence data suggested that F11 therapy may eliminate ABLV‐luc infection in the weeks following treatment, we could not be certain that virus did not continue replicating at levels beneath the limit of detection of this imaging modality. Thus, we performed additional infections of WT animals, with and without F11 treatment, to collect brains for analysis of total RNA by quantitative real‐time PCR (qRT‐PCR). For these experiments, we employed the primer set used in Fig 6F to detect positive‐sense viral mRNA encoding the nucleoprotein (*N*), and we also designed a primer set to detect negative‐sense genomes at the *M*‐*G* junction (see Materials and Methods for details). The inclusion of both probe sets was necessary to determine whether detected ABLV RNA represented actively transcribed genomes, in which case the abundance of *N*‐transcripts (representing the summation of *N*‐mRNAs and positive‐sense genomes) would be present in excess over negative sense genomes detected by the M‐G probe set (Finke & Conzelmann, 1997). By contrast, if only non‐replicative viral particles were present, the abundance of each type of RNA would be equivalent.

We employed known amounts of DNA standards to enable absolute quantification of *N*‐transcripts and negative‐sense genomes in uninfected controls, ABLV‐luc‐infected untreated animals, and ABLV‐luc‐infected F11 treated animals. As expected, ABLV RNAs were abundant in the brains of infected untreated animals, with as many as 3 × 10^6^ *N*‐transcripts and 2 × 10^5^ negative‐sense genomes per μg of RNA (Fig 6G, Table 2). In comparison to day 10 post‐infection when replicating virus is first evident by luciferase imaging, viral loads were higher at days 12–15, which is coincident with the expected mortality window (Fig 6G, Table 2). Surprisingly, the brains of day 7 F11‐treated, ABLV‐infected animals uniformly exhibited presence of viral RNAs, albeit at levels 2–3 logs lower than observed in untreated animals (approximately 4 × 10^3^ *N*‐transcripts and 1 × 10^3^ genomes per μg of brain RNA). These viral RNAs were detected at a relative narrow copy number range across eleven animals and throughout all assessed time points (days 13–139 post‐infection). Notably, in an independent study (Mastraccio *et al*, 2020), we have determined that below approximately 10^4^ *N*‐transcripts per μg of total brain RNA, the measured RLUs are generally indistinguishable from background, explaining why this persistent viral burden was not detected by bioluminescence imaging.

**Table 2:** qRT‐PCR detection of ABLV RNA species.

Mouse ID	Days post‐infection	QPCR Run 1			QPCR Run 2		
		ABLV N RNA (+)^a^	ABLV M‐G RNA (−)^a^	N/M‐G Ratio	ABLV N RNA (+)^a^	ABLV M‐G RNA (−)^a^	N/M‐G Ratio
Uninfected, Untreated							
UU2A	15	ND	ND	N/A	ND	ND	N/A
UU3A	15	ND	ND	N/A	ND	ND	N/A
UU0B	14	ND	ND	N/A	ND	ND	N/A
Average		N/A	N/A	N/A	N/A	N/A	N/A
Infected, Untreated							
IU0A	15	125,152	18,837	6.6	109,984	17,022	6.5
IU1A	15	1,067,634	85,547	12.5	1,355,210	91,231	14.9
IU2A	13	2,929,457	183,693	15.9	2,783,488	193,351	14.4
IU3A	13	1,109,930	120,448	9.2	1,075,543	123,369	8.7
IU4A	15	607,569	41,004	14.8	547,564	41,488	13.2
IU0B	12	678,057	65,285	10.4	638,719	66,448	9.6
IU1B	10	117,686	11,374	10.3	124,452	10,912	11.4
IU2B	10	176,588	19,767	8.9	164,662	18,915	8.7
IU3B	12	505,512	43,557	11.6	465,438	42,362	11.0
IU4B	14	567,562	72,025	7.9	480,823	75,013	6.4
Average		788,515	66,154	10.8	774,588	68,011	10.5
Infected, F11 day 7							
I7F0A	47	4,169	1,581	2.6	2,992	1,270	2.4
I7F1A	15	9,686	1,080	9.0	7,113	835	8.5
I7F2A	13	3,570	1,072	3.3	2,802	767	3.7
I7F3A	47	6,317	1743	3.6	5,571	1,288	4.3
I7F0B	13	3,962	777	5.1	2,814	540	5.2
I7F1B	47	2040	1,052	1.9	1,683	848	2.0
I7F2B	47	5,090	2090	2.4	4,173	1,568	2.7
I7F0C	22	4,930	562	8.8	4,312	430	10.0
I7F1C	126	2,220	573	3.9	1,607	343	4.7
I7F2C	139	2,477	926	2.7	1,659	671	2.5
I7F3C	139	6,307	917	6.9	4,546	690	6.6
Average		4,615	1,125	4.6	3,570	841	4.8

N/A, not applicable; ND, none detected.

(+), template cDNA synthesized from a specific primer annealing to N mRNAs and anti‐genomes.

(−), template cDNA synthesized from a specific primer annealing to viral genomes.

^a^ Viral RNAs are copies/μg of total RNA purified from a brain hemisphere at time of euthanasia.

Calculation of the ratio of positive‐sense *N* transcripts to negative sense genomic transcripts demonstrated that *N* transcripts were consistently present in excess over genomic transcripts in both untreated and F11‐treated animals. However, the average *N*/genomes ratio was higher in untreated animals (approximately 11‐fold) as compared to treated animals (approximately 5‐fold). Data were comparable between two independent qRT‐PCR measurements of transcript abundance (Table 2). Thus, the brains of animals infected with ABLV‐luc and treated with F11 at day 7 remain persistently infected with a low level of transcriptionally active virus through at least day 139 post‐infection, despite reversal of disease signs by day 20 in most animals, followed by long‐term disease‐free status (Fig 1).

To determine whether this long‐term low‐level infection with ABLV was accompanied by evidence of a persistent cellular immune response, we performed RT‐PCR to semi‐quantitatively detect the mRNA products of the genes encoding interferon‐γ (*Ifng*) and perforin (*Prf1*), two hallmark effector proteins of cellular immune responses. These RT‐PCR data demonstrated elevation of both immune response genes in all F11‐treated animals, with the fold elevation at later time points (day 47 and beyond) ranging from 8‐ to 51‐fold above uninfected controls (Table 3). Notably, however, expression of these genes was even higher in infected, untreated animals, with immune response gene levels generally correlating with ABLV RNA loads (Fig 6G, Tables 2 and 3). Thus, these data show that elevated transcription of cellular immune response genes occurs in animals infected with ABLV‐luc and persists in F11‐treated animals. Additionally, the intensity of *Ifng* and *Prf1* transcription correlates with the load of transcribed viral RNA.

**Table 3:** RT‐PCR analysis of cellular immune response gene expression.

Mouse ID	Days post‐infection	ABLV N RNA (+)^a^	*Ifng* Fold change^b^	*Prf1* Fold change^b^
Uninfected, Untreated				
UU2A	15	ND	N/A	N/A
UU3A	15	ND	N/A	N/A
UU0B	14	ND	N/A	N/A
Infected, Untreated				
IU0A	15	125,152	499	190
IU1A	15	1,067,634	1,119	535
IU2A	13	2,929,457	851	454
IU3A	13	1,109,930	854	383
IU4A	15	607,569	1,644	422
IU0B	12	678,057	351	130
IU1B	10	117,686	30	10
IU2B	10	176,588	65	18
IU3B	12	505,512	351	123
IU4B	14	567,562	596	400
Infected, F11 day 7				
I7F0A	47	4,169	32	22
I7F1A	15	9,686	222	87
I7F2A	13	3,570	24	8
I7F3A	47	6,317	32	16
I7F0B	13	3,962	23	9
I7F1B	47	2040	16	16
I7F2B	47	5,090	18	32
I7F0C	22	4,930	119	64
I7F1C	126	2,220	20	15
I7F2C	139	2,477	16	19
I7F3C	139	6,307	51	32

N/A, not applicable; ND, none detected.

^a^ Viral RNAs are copies/μg of total RNA purified from a brain hemisphere at time of euthanasia.

^b^ Fold change vs. uninfected, untreated group, calculated by ΔΔCT method.

### Fc‐FcRγ interactions contribute to the therapeutic efficacy of F11

**Figure 7:**
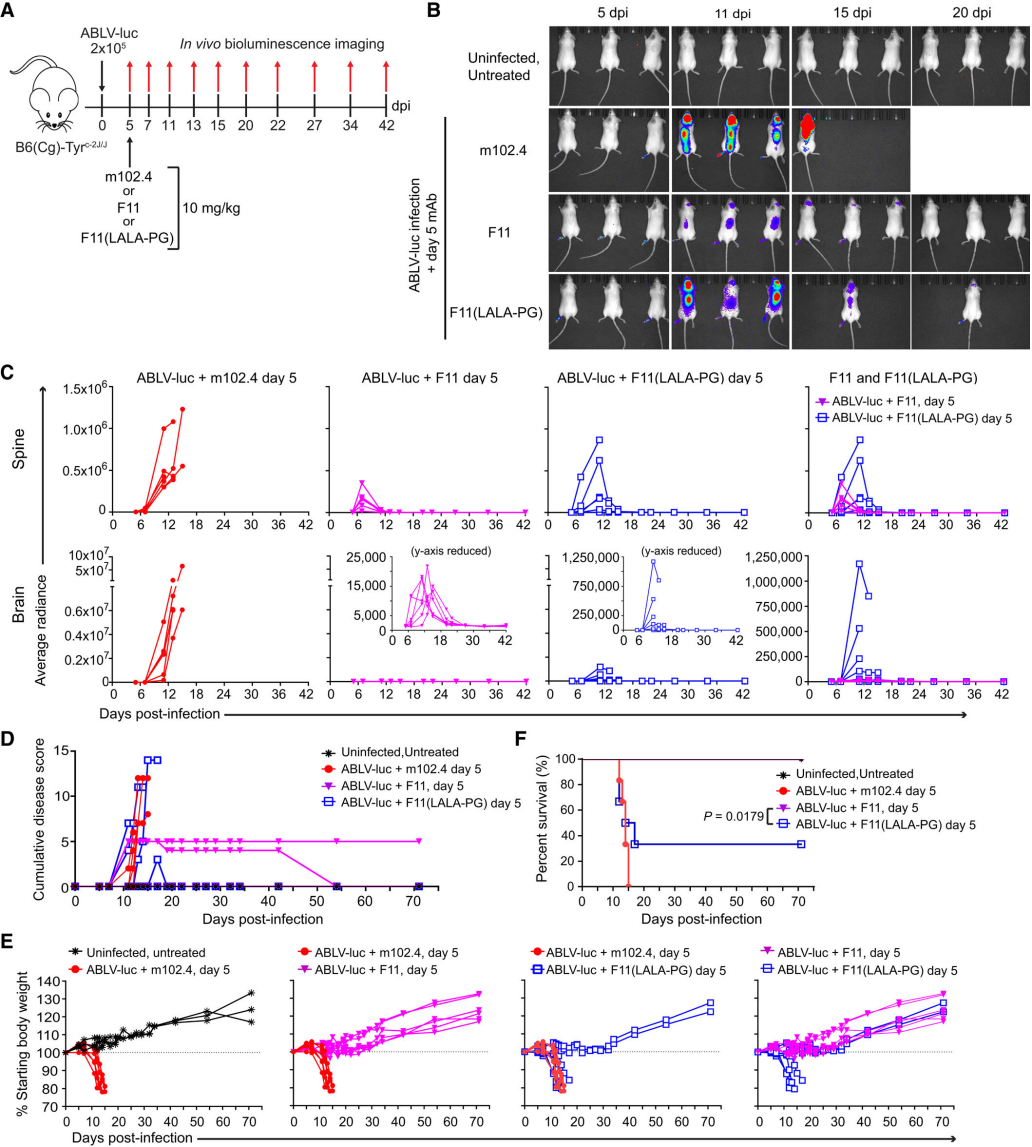
FcRγ binding activity of F11 contributes to *in vivo* therapeutic efficacy B6 albino mice were infected with 2 × 10^5^ FFU of ABLV‐luc on day 0. On day 5 post‐infection, mice received 10 mg/kg m102.4, F11, or F11(LALA‐PG) (*n* = 3 mice/group for uninfected; *n* = 6 mice/group for all others).Bioluminescence imaging of mice on days 5, 11, 15 and 20 post‐infection, using an IVIS Spectrum CT (Perkin‐Elmer). See Appendix Fig S3 for full series.Viral burden was quantified as average radiance in spines (top row) and brains (bottom row) of mice. Insets are same data with reduced y‐axis scale, to show detail.Cumulative disease scores were determined by clinical presentation following ABLV‐luc challenge. The one F11‐treated animal with a persistent disease score of 5 had partial paralysis in the inoculated limb.Percent starting body weight as an indicator of disease.Kaplan–Meier survival plot, Logrank test: ABLV‐luc, F11 vs. ABLV‐luc, F11(LALA‐PG), *P* = 0.0179. Source data are available online for this figure.

Collectively, the above data strongly suggest a functional interaction between F11 and the host immune response. One way in which IgG antibodies can influence immune responses is via interactions with FcRγ on host immune cells (Nimmerjahn & Ravetch, 2007; DiLillo & Ravetch, 2015), an interaction that remains functionally relevant when studying the biology of human mAbs in the mouse model (Overdijk *et al*, 2012). To ascertain the possible contributions of FcRγ binding to F11 therapeutic efficacy, we generated a version of F11 in which the LALA‐PG point mutations were introduced, in order to impair Fc/FcRγ interactions and interaction with complement protein C1q; importantly, these mutations do not impact binding to FcRn, an interaction that results in the long *in vivo* half‐life of IgG (Lo *et al*, 2017). Additionally, the LALA‐PG mutations have no effect on the capacity of F11 to neutralize ABLV *in vitro* (Appendix Fig S3A). We directly compared the ability of F11 and F11(LALA‐PG) to control *in vivo* infection with ABLV‐luc. Negative control groups were uninfected, untreated mice and ABLV‐luc infected mice injected with m102.4 (Fig 7A). All antibodies were injected at day 5 post‐infection, which is the time point at which F11 therapy has the optimal impact (Fig 1). Bioluminescence imaging demonstrated detectable viral replication in the spines and brains of infected animals beginning on day 7 (Fig 7B and C; Appendix Fig S3B). m102.4 treated animals exhibited luminescence levels in the spine generally higher than those treated with F11 and F11(LALA‐PG) during days 7–14 of infection. However, luminescence levels in the brain were clearly stratified based on the mAb administered, with m102.4‐treated animals reaching average radiance values in the range of ~0.5–5 × 10^7^; F11(LALA‐PG)‐treated animals at 0.12 × 10^7^ or less; and F11‐treated animals at 0.0025 × 10^7^ or less. Thus, ABLV‐luc replication was well controlled in the brains of F11‐treated animals, but only modestly controlled in the brains of animals treated with F11(LALA‐PG).

With regard to disease progression, mice treated with the irrelevant mAb m102.4 exhibited an increasing disease score and rapid weight loss (Fig 7D and E), with all animals reaching endpoint criteria by day 14 (Fig 7F). Importantly, while all animals treated with F11 experienced limited morbidity with 100% survival, 66% animals treated with F11(LALA‐PG) exhibited disease signs and mortality kinetics indistinguishable from m102.4‐treated animals. The difference in mortality between F11 and F11(LALA‐PG) was significant (*P* = 0.0179) (Fig 7D–F). These data demonstrate that impairment of Fc‐FcRγ interactions substantially reduces the efficacy of F11 in preventing morbidity and mortality.

### Little F11 enters the brain of ABLV‐infected animals

Finally, we assessed whether the therapeutic effects of F11 may reflect direct penetration of F11 into the brains of infected animals. For these experiments, we performed microscopy analyses in which we attempted to detect F11 using a fluorescently labeled anti‐human IgG. Although we did detect sporadic cell‐associated F11 on day 14 post‐infection (Appendix Fig S4), there was no evidence of presence of widespread F11 in the brains of treated animals, a result consistent with other studies of mAb CNS penetrance during rabies infections (Hooper *et al*, 2009). These data are thus consistent with our overall model that the protective effect of F11 in the brains of lyssavirus‐infected animals is most likely mediated by peripheral actions of F11 that change the functionality and CNS abundance of immune cell subsets, particularly CD4 T cells.

## Discussion

In this study, we have shown that a single dose of human mAb F11 can yield long‐term control of ABLV and RABV infection, even when administered after occurrence of robust virus replication in the CNS and appearance of disease signs. Importantly, F11 treatment on day 5 or day 7 post‐infection mediates resolution of clinical disease and long‐term survival, with no recurrence of disease signs or evidence of robust viral replication through at least 140 days post‐treatment. We administered F11 at a dose of 10 mg/kg, consistent with the generally recommended dosing of mAbs for human therapeutic efficacy (Samaranayake *et al*, 2009). With a half‐life of approximately 13 days, 140 days is more than ten half‐lives, corresponding to greater than a 1,000‐fold reduction from the starting *in vivo* concentration. We recently reported that the IC_50_ for mAb F11 is 0.6 ng/ml for ABLV and several other phylogroup I lyssaviruses (Weir *et al*, 2021). While we measured the initial *in vivo* concentration of F11 at 154 ng/ml, a value well above the IC_50_, the concentration is reduced to 0.15 ng/ml after 10 half‐lives, which is below the IC_50_, strongly suggesting that meaningful neutralization no longer occurs at such late post‐therapy time points.

Published data have shown that antibody‐mediated virus neutralization is essential for clearance of established infections with non‐lethal strains of rabies (Hooper *et al*, 1998). Although the simplest mechanism to explain F11 efficacy is virus neutralization, the *in vivo* decay of viral titer over the time span of the observed viral suppression suggests that mechanisms beyond neutralization contribute to long‐term control of lyssavirus infection. Indeed, the observation that F11 delays but does not prevent mortality in the NSG, NOD‐SCID, and RAG1‐KO strains demonstrates that the host adaptive immune response plays an essential role in control of viral infection and prevention of death. Several lines of evidence identify CD4 T cells as the adaptive immune population that is mechanistically required for F11 efficacy. In muMT^–^ mice, which lack mature B cells and the ability produce antibodies, the efficacy of F11 therapy was indistinguishable from the effect seen in WT animals. Also, while animals depleted of CD8 T cells exhibited an extended kinetic of a very low level of luciferase positivity in the brain following F11 therapy, all animals ultimately survived, exhibiting neither disease signs nor recurrence of detectable virus‐produced luciferase in the CNS through 82 days post‐infection. These same CD8‐depleted animals also had a protective immune response to a superinfection with ABLV‐luc. By contrast, mice lacking CD4 T cells (MHCII^−^) exhibited a persistent modest level of luciferase positivity (substantially higher than observed in CD8 T cell depleted animals) in the brains and spinal cords of infected, F11‐treated animals through the entire course of infection, analogous to our observations in NSG mice and the other strains completely lacking adaptive immune cells. All of the MHCII^–^ mice ultimately reached euthanasia criteria between days 40 and 90 post‐infection, coincident with an increase in disease signs and the intensity of ABLV‐luc bioluminescence in the CNS, indicative of increased viral burden (Mastraccio *et al*, 2020). Thus, while animals deficient in B cells and CD8 T cells are able to control ABLV‐luc infection and survive long‐term in response to F11 therapy, mice lacking a functional CD4 T cell compartment are not.

With regard to mechanistic consequences of F11 therapy on CNS immune responses, we demonstrated significant alterations in the leukocyte infiltrate in the brains of infected, F11‐treated animals. In particular, in comparison to animals given the irrelevant mAb m102.4, F11‐treated animals exhibit increased proportions of T cells, particularly CD4 T cells. Studies employing the non‐fatal rabies virus strain, CVS‐F3, have shown that clearance of viral infection is dependent on regulation of BBB permeability by effector CD4 T cells that migrate to the CNS (Phares *et al*, 2007; Fabis *et al*, 2008). In the context of these observations, one possibility is that F11 therapy augments the production of CD4 effector T cells that are able to enter the CNS and regulate BBB permeability, enabling further recruitment of cells able to mediate protection against lethal lyssavirus strains. Further studies will be required to more fully elucidate the role of CD4 T cells in protection of F11‐treated animals from lethality.

We also showed that F11‐enhanced recruitment of CD4 T cells directs or is accompanied by activation (MHCII expression) by CD11b^+^ Ly6C^−^ myeloid cells, a phenotype shared by activated microglia and activated macrophages and monocyte‐derived dendritic cells (moDCs) in the brain (Greter *et al*, 2015). In this regard, it is interesting that *in vivo* (Garcia *et al*, 2018) and *in vitro* (Feige *et al*, 2022) studies have identified resident immune cells in the brain, including microglia, as playing a key role in protection against pathogenic RABV. Myeloid cell activation is minimal in the brains of animals treated with an irrelevant mAb. We also demonstrated that the ability of F11 to interact with FcRγ contributes significantly to protection from mortality. This result also aligns with the study of de Melo *et al* (2020), in which the LALA mutants of the two therapeutic mAbs resulted in higher viral loads and greatly diminished protection from mortality.

Despite the apparent F11‐mediated elimination of lyssavirus infection suggested by our luciferase imaging data, qRT‐PCR analysis showed that rabies infection persists at a low level in the brains of F11‐treated animals for at least 139 days post‐infection. We also demonstrated that these chronically infected animals have persistently elevated expression of the cellular immune response genes, *Ifng* and *Prf1*, in the brain. Notably, IFNγ is a potent activator of myeloid cells (Schroder *et al*, 2004), and a known inducer of MHCII expression (Steimle *et al*, 1994). Thus, IFNγ may contribute to the robust MHCII expression on myeloid cells that occurs only in the brains of ABLV‐infected, F11‐treated animals. In this regard, it is notable that CD4 T cell production of IFNγ is critical for clearance of the attenuated RABV strain, CVS‐F3 (Lebrun *et al*, 2015; Bongiorno *et al*, 2017). However, it is important to note that untreated ABLV‐infected animals also show elevation of *Ifng* and *Prf1* transcription in a manner roughly proportional to viral load. Thus, it is unclear whether the persistent elevation in *Ifng* and *Prf1* transcription represents a mechanism that contributes to the control of lyssavirus infection in F11‐treated animals, or if it is a response that accompanies infection, while expression of a distinct immune mediator is the critical determinant of immunological control of lyssavirus infection. Considered collectively, the data in our study suggest that F11 therapy alters the cellular response to lyssavirus infection in the brain, such that a higher proportion of CD4 T cells and specific subsets of activated myeloid cells are present in higher proportions in comparison to untreated, infected animals. Protection from death requires CD4 T cells, Fc effector function, and is accompanied by persistent elevation of cytokine mRNAs that are characteristic of cellular immune responses.

Our observation of post‐therapy lyssavirus persistence is reminiscent of data from human survivors of other highly lethal RNA virus infections. For example, survivors of Ebola virus infection continue to shed virus in bodily fluids for weeks to months following resolution of clinical disease and absence of detectable virus in the blood (Chughtai *et al*, 2016; Caviness *et al*, 2017); data suggest these reservoirs are present at immune privileged sites (including the CNS), where the activity of the adaptive immune response is limited (Sagui *et al*, 2015; Billioux *et al*, 2017; Howlett *et al*, 2018; Iversen *et al*, 2020). Similarly, a subset of survivors of infection with Nipah virus exhibit subsequent encephalitis, years following apparent clearance of the initial infection. Postmortem data of patients who succumbed to this late‐onset encephalitis indicate Nipah virus involvement, attributable to long‐term persistence in the brain (Tan *et al*, 2002; Dawes & Freiberg, 2019). Notably, the CNS is an immune privileged site, and our observations of long‐term persistence of ABLV in the CNS of F11‐treated animals thus shares key features of Ebola virus and Nipah virus persistence. Indeed, emerging evidence suggests that many RNA viruses have evolved immune‐evasive strategies that enable long‐term persistence. Although individuals may appear free of disease for months or years, recurrence of symptomatic viral infection, sometimes fatal, is not uncommon (McCarthy & Morrison, 2017). Whether lyssavirus infection may recur in F11‐treated animals as a result of post‐therapy immunocompromise is an important question that should be addressed by future studies.

For human therapeutic applications, passive administration of anti‐viral mAbs has historically been used almost exclusively with the goal of neutralizing infectious virus prior to or shortly after initiation of infection, and mAb therapy was previously considered of no value for treatment of established infections (Walker & Burton, 2018). Consistent with this paradigm, the use of anti‐rabies mAbs has been explored primarily as a more easily obtained and cost‐effective alternative to HRIG for use in conjunction with the rabies vaccine for PEP (Schumacher *et al*, 1989; Dietzschold *et al*, 1990, 1992; Prosniak *et al*, 2003; de Kruif *et al*, 2007; De Benedictis *et al*, 2016; Rupprecht *et al*, 2016). In this work, through use of the LALA‐PG F11 mutant that impairs interaction with host FcRγ, we demonstrated that Fc function contributes significantly to protection of animals from mortality due to lyssavirus encephalitis, providing evidence for a mechanistically essential contribution of the mAb Fc region. Moreover, recent data from preclinical studies in other viral models strongly suggest that mAb therapy can also have high therapeutic efficacy against established viral infections (Keeler & Fox, 2021). For example, studies of HIV infections in primate models suggest that at least some mAbs can be used successfully to treat established SHIV infections (Barouch *et al*, 2013; Shingai *et al*, 2013). Additionally, a study of alphavirus infection in a mouse model demonstrated that antibody isotype is a critical determinant of mAb efficacy against established viral infection. Specifically, isotypes with high Fc receptor (FcRγ) avidity (murine IgG_2a_ and its functional equivalent, human IgG1) were much more efficacious as antiviral therapies *in vivo* than isotypes that are bound by FcRγs poorly. The implication of this study is that FcRγ‐dependent immune effector functions enhance the efficacy of mAbs that are protective against established viral infections (Earnest *et al*, 2019). Similarly, a study of mAb therapy for Chikungunya virus (CHIKV) infection showed that an interaction between mAb Fc and monocyte FcRγ is crucial for efficient CHIKV clearance (Fox *et al*, 2019). Also, Fc function was required for optimal mAb therapeutic efficacy in a mouse model of SARS‐CoV‐2 infection (Ullah *et al*, 2021). Collectively, these data are similar to our findings demonstrating contributions of FcRγ interaction to F11 *in vivo* efficacy.

Finally, with regard to a recently published study reporting mAb‐mediated clearance of RABV infection following appearance of disease signs (de Melo *et al*, 2020), we believe that, combined with the data in this manuscript, there is now compelling evidence to suggest that anti‐lyssavirus mAbs may have potential as a therapeutic approach for human rabies. Impressively, de Melo *et al* reported complete clearance of RABV. However, to achieve this impressive result, their study required two antibodies delivered via a combination of peripheral intramuscular (IM) administration plus continuous intracerebral infusion. Importantly, peripheral IM delivery by itself led to little benefit (de Melo *et al*, 2020). By contrast, although our systemic, single‐dose therapy left animals with a chronic lyssavirus infection in the CNS, replication was maintained at a low level for at least 139 days, with no recurrence of disease signs. Thus, F11 therapy deployed through the circulatory system appears to deliver a functional cure, despite viral persistence. Given that the vast majority of human rabies occurs in rural areas of the developing world with limited healthcare resources, we would argue that a single‐dose, easily administered therapy is a much more viable approach for human therapy than a treatment that requires highly invasive, continuous infusion to the CNS. Clearly, however, much future work would be required before either therapy could be considered for human use.

When considered broadly, our findings have important implications for the clinical development and use of mAb therapy for viral infections. Similar to other recent studies mentioned above, our data show that mAb therapy involves Fc‐FcRγ interactions that are ultimately essential for therapeutic efficacy. We thus suggest that candidate therapeutic antiviral mAbs should be evaluated both in preclinical studies and in clinical trials for provocation of a pathogen‐directed adaptive immune response, in addition to evaluation of simple virus neutralization. Of additional importance, with regard to the treatment of neurotropic infections, although intravenous antibody therapy is generally not considered as a viable option for pathogens in the CNS parenchyma because of BBB exclusion, our data strongly suggest that antiviral mAbs should not be dismissed as a therapeutic option for the treatment of such infections, due to the potential for provocation of a CNS‐directed adaptive immune response. Thus, it would be prudent to investigate whether mAb therapy may be effective against other neurotropic pathogens, even beyond the point of CNS infection.

## Materials and Methods

### Mice

Mice were 8(±1) week‐old female C57BL/6J (RRID: IMSR_JAX:000664), B6(cg)‐*Tyr* ^*c‐*2J^/J (B6 albino) (RRID: IMSR_JAX: 000058), B6.129S2‐*Ighm* ^*tm1Cgn*^/J (MuMt^−^) (RRID: IMSR_JAX: 002288), NOD.CB17‐*Prkdc* ^*scid*^/J (NOD scid) (RRID: IMSR_JAX:001303) or 8 (±1) week‐old male and female NOD.Cg‐*Prkdc* ^*scid*^ *Il2rg* ^*tm1Wjl*^/SzJ (NSG) (RRID: IMSR_JAX: 005557), B6.129S7‐*Rag1* ^*tm1Mom*^ (Rag1 KO) (RRID: IMSR_JAX: 002216) and B6.129S2‐H2^d/Ab1‐Ea^/J (MHCII^−^) (RRID: IMSR_JAX: 003584) obtained from Jackson Laboratories. All mice weighed 20 (±4) grams. For F11 treatment studies, mice were randomized into treated and control groups. Viral load, body weight, cumulative disease scores, and survival of F11‐treated mice were compared to untreated controls. For CD8 T cell depletion studies, mice were randomized into treated or control groups. Antibody blockade of CD8 T cells was compared to isotype control antibody‐treated mice. Sample sizes for experimental groups and their respective controls are indicated in the figure legends. For all mouse infection studies, endpoint criteria consisted of ≥20% weight loss. Isotype control C57BL/6J mice in Fig 4A–C and Appendix Fig S5 were repeated as the control group for the experiment in Fig 5 and Appendix Fig S6. All animal experiments were performed under ABSL‐2 conditions in the USU specific pathogen free animal facility and in accordance with and approval of Uniformed Services University Institutional Animal Care and Use Committee (IACUC) (protocol MIC‐20‐926) and Institutional Biosafety Committee guidelines.

### Cell lines and viruses

HEK293T cells (ATCC Cat#CRL‐3216, RRID: CVCL_0063, female) and Neuro‐2a (N2a) cells (ATCC Cat#CCL‐131, RRID: CVCL_0470, male) were grown in Dulbecco's Modified Eagle's Medium (DMEM) (GE Healthcare Life Sciences) supplemented with 10% fetal bovine serum (FBS) (Sigma Aldrich) and a 2% cocktail of penicillin (Sigma Aldrich), streptomycin (Sigma Aldrich), gentamycin (GoldBio), and L‐glutamine (Corning) at 37°C, 5% CO_2_. For *in vitro* and *in vivo* ABLV infection studies, a firefly luciferase (FLuc)‐expressing ABLV reporter virus (ABLV‐luc) was employed as described (Mastraccio *et al*, 2020). For rabies challenge studies, a rodent‐adapted RABV strain (CVS‐11) was used (ATCC Cat#VR‐959). The purchased CVS‐11 stock was amplified in BHK‐21 clone 13 cells (ATCC Cat#CCL‐10, RRID: CVCL_1915) as per the instructions provided by ATCC.

### Human mAbs F11, F11(LALA‐PG) and m102.4

Human anti‐ABLV G mAb F11 was isolated and characterized as described (Weir *et al*, 2021). PCR mutagenesis was used to introduce the LALA‐PG mutations (Lo *et al*, 2017) into the coding sequence of the F11 heavy chain, to generate F11(LALA‐PG). The anti‐henipavirus G mAb m102.4 was described previously (Playford *et al*, 2020). All mAb expression constructs were stably expressed in 293F cells, and mAb was purified from the supernatant by Protein G Sepharose affinity chromatography.

### *In vitro* neutralization assays

ABLV‐luc or CVS‐11 was added to 10‐fold serial dilutions of mAb F11 at a multiplicity of infection (MOI) of 0.1. Following a 1.5 h incubation at 37°C, 5% CO_2_, the virus‐antibody mixture was added to N2a cells (triplicate wells) in a volume of 2 ml supplemented DMEM and incubated for an additional 48 h at 37°C, 5% CO_2_. Cells were then stained with DAPI (Thermo Fisher), mouse anti‐rabies G (Novus Biologicals Cat#NBP2‐11630), and Alexa Fluor 647 goat anti‐mouse IgG2a (Thermo Fisher Cat#A‐21241, RRID: AB_2535810) and analyzed via confocal microscopy. Neutralization titer is expressed as the first dilution at which no anti‐rabies G fluorescence is observed in any of the triplicate wells.

### Determination of F11 *in vivo* half life

C57BL/6 mice (4 per group) were injected with 10 mg/kg F11. Sera were collected by terminal bleed at 1, 7, 14, 21, 28 and 56 days post‐injection. F11 concentrations were determined using a human IgG ELISA Pair Kit (STEMCELL Technologies, Cat# 01994). F11 was spiked into naïve C57BL/6 serum and serially diluted in incubation buffer (PBS containing 0.05% Tween20 and 0.1% bovine serum albumin) to generate a standard curve with a concentration range of 0.4 μg/μl to 0.78 ng/μl. The mean absorbance values of duplicate standard wells were processed by CurveExpert 1.4 to identify the best fit standard curve equation. The resulting equation was used to calculate the concentration of F11 present in sera from F11‐injected mice. F11 concentration was then plotted as a function of time post‐injection, using GraphPad Prism 7.05. Finally, half‐life was calculated using a non‐linear regression curve with a one‐phase exponential decay function and a least squares fit. All standard solutions and F11‐treated mouse sera samples were assayed in duplicate wells.

### Viral challenge studies in mice

Mice were inoculated in the right rear footpad with the indicated doses of ABLV‐luc in a volume of 20 μl DMEM. In a separate study, C57BL/6J mice were inoculated in the right rear footpad with 2 × 10^5^ FFU of CVS‐11. All mice were monitored twice daily and scored for disease progression with an internal scoring protocol approved by the USU IACUC. Cumulative disease scores were calculated as 0 = healthy, 1 = lethargy, 2 = weight loss, 3 = tremors, 4 = uncoordinated gait, and 5 = hind‐limb paralysis.

### mAb treatment

Mice were infected with 2 × 10^5^ FFU of ABLV‐luc as described above, followed by treatment with a single dose of mAb F11 (Weir *et al*, 2021) on day 3, 5, or 7 post‐infection. In the indicated experiments, F11 was compared to two other human IgG1 mAbs, F11(LALA‐PG) and/or m102.4 (Playford *et al*, 2020), with antibody administration on day 5 post‐infection. mAbs were administered intraperitoneally at a dose of 10 mg/kg. CVS‐11‐infected mice were administered F11 on day 5 or 7 post‐infection. For experiments with B6.129S2‐*Ighm* ^*tm1Cgn*^/J (MuMt^−^), NOD.Cg‐*Prkdc* ^*scid*^ *Il2rg* ^*tm1Wjl*^/SzJ (NSG), NOD.CB17‐*Prkdc* ^*scid*^/J (NOD‐scid), B6.129S7‐*Rag1* ^*tm1Mom*^ (Rag1 KO), B6.129S2‐H2^d/Ab1‐Ea^/J (MHCII^−^), and CD8 T cell‐depleted C57BL/6J mice, F11 was administered on day 5 post‐infection only.

### *In vivo* CD8^+^ T cell depletion

To optimize the dosing regimen for CD8 T cell depletion, C57BL/6J mice received 300 μg of anti‐CD8α mAb (Bio X Cell Cat#BE0061, RRID:AB_1125541) or isotype control (Bio X Cell Cat# BE0090) mAb via the intraperitoneal route on days −3, −1, 6, 13, and 20. Spleens were harvested on days 5, 10, 17, and 25 and single‐cell suspensions were stained with APC‐H7 rat anti‐mouse CD8α (BD Biosciences Cat#560182, RRID: AB_1645237), PerCP/Cy5.5 rat anti‐mouse CD45 (BD Biosciences Cat# 561869), and PE rat anti‐mouse CD4 (BD Biosciences Cat# 553049). To confirm CD8 T cell depletion following ABLV‐luc infection, spleens were harvested from an isotype control mouse and a CD8 T cell‐depleted mouse on day 5 post‐infection. Single‐cell suspensions were stained with Alexa Fluor 647 rat anti‐mouse CD8α (clone 53‐6.7, purified from hybridoma supernatants and labeled in‐house), PerCP/Cy5.5 rat anti‐mouse CD45 (BD Biosciences Cat# 561869), and PE rat anti‐mouse CD4 (BD Biosciences Cat# 553049). Flow cytometry was performed with a BD LSRII flow‐cytometer, and data were analyzed with FlowJo Software.

### *In vivo* bioluminescence imaging

Starting on day 3 or day 5 post‐infection, mice were imaged three times a week for 2 weeks then once a week until no above‐background luciferase signal was detected for at least two sequential imaging sessions. Ten minutes prior to imaging, mice were administered D‐luciferin, potassium salt (GoldBio Cat#LUCK1G) via the intraperitoneal route. Stock solutions of D‐luciferin were prepared in DPBS at 15 mg/ml and each mouse received 150 mg luciferin/kg body weight. During each imaging session, mice were anesthetized with isoflurane and imaged in groups of three for 1 min using the In‐Vivo Xtreme II system (Bruker) or the IVIS Spectrum CT (Perkin‐Elmer). Contrast levels of X‐ray images were adjusted using MI software (Bruker). Scaling of the luminescence overlays was standardized for all groups of mice and mean luminescence intensity (MLI) or average radiance values were calculated using MI software (Bruker) or Living Image software (Perkin‐Elmer). Images were exported to Adobe Photoshop for cropping.

### Purification of brain leukocytes

On day 14 or 15 post infection, mice received 2 μg APC anti‐CD45 (clone 30‐F11, BioLegend, cat #103112) i.v. 3 min before collecting brain tissue, in order to label blood leukocytes for exclusion from subsequent flow cytometry analysis (Urban *et al*, 2020). Brain mononuclear cells were isolated as previously described with minor modification (Guldner *et al*, 2021). Brain tissue from individual animals were minced gently in RPMI 1640 medium (Thermo Fisher, cat #11875093) with a disposable surgical scalpel and incubated with collagenase D (1 mg/ml; Roche, cat #11088858001) and DNase I (50 μg/ml; Roche, cat #10104159001) for 30 min in a shaking incubator at 37C. Digested tissues were passed twice through a 70 μm cell strainer (Falcon, cat #352350) and then through a 40 μm cell strainer (Falcon, cat #352340) to remove cell debris and obtain single‐cell suspensions. Cells were spun down at 300 *g* for 10 min at 4°C. The cell pellets were resuspended with myelin removal beads (Miltenyi Biotec, cat #130‐096‐433) and incubated on a tube rotator for 15 min in 4°C. Samples were passed through an LS column (Miltenyi Biotec, cat #130‐042‐401) according to the manufacturer's protocol to remove myelin debris. Effluent was collected and spun down at 500 *g* for 5 min at 4°C. The cell pellets were resuspended in ice‐cold 90% Percoll (Cytiva, cat #17‐0891‐02) and overlaid with 60% Percoll solution, followed by 40% Percoll solution and subsequently by 1 × Hanks balanced salt solution (HBSS, Thermo Fisher, cat #14185052). Cells were isolated by centrifugation at 600 *g* for 20 min at 4°C with the brake disengaged. The cell fraction located between the 60 and 40% interphase was carefully aspirated. Cell suspensions were washed with 1 × HBSS and centrifuged at 2,000 *g* for 10 min at 4°C. Cell pellets were resuspended in FACS buffer (PBS + 0.5% FBS), enumerated, and used for flow cytometry.

### Flow cytometry

Single‐cell suspensions were plated and stained with a single‐color LIVE/DEAD Blue fixable dead‐cell stain (1:400; Invitrogen) for 30 min. Cells were washed, Fc receptors were blocked with Mouse TruStain FcX (CD16/32; 1:100; BioLegend, cat #101320) and mouse IgG (1:50; Jackson Immunoresearch, cat #015‐000‐003) at 4°C for 15 min, and then cells were labeled with a combination of fluorescently labeled antibodies that are specific for immune cell surface markers at RT for 20 min, including CD8a (53‐6.7; 1:100; BD Bioscience, cat #612898), CD4 (RM4‐5; 1:100; BD Bioscience, cat #563151), CD3e (145‐2C11; 1:40; BD Bioscience, cat #562286), CD45R/B220 (RA3‐6B2; 1:66; BD Bioscience, cat #562922), CD11b (M1‐70; 1:100; BD Bioscience, cat #553310), I‐A/I‐E (M5/114.15.2; 1:33; BioLegend, cat #107643), Ly6C (HK1.4; 1:80; BioLegend, cat #128030), CD44 (IM7; 1:80; BioLegend, cat #103029), CD62L (MEL‐14; 1:40; BD Bioscience, cat #553151), CD25 (PC61; 1:40; BD Bioscience, cat #564023), NK1.1 (PK136; 1:66; BD Bioscience, cat #751111). After staining, cells were fixed with ice cold 4% paraformaldehyde for 10 min. All samples were acquired using the Cytek Aurora (Cytek Biosciences) and analyzed using FlowJo software (FlowJo LLC). Cells were gated on singlets, live cells, and CD45‐negative cells (to exclude labeled peripheral blood leukocytes; see above). Quantified populations were then gated as follows with quantification reflecting the % of live, CD45‐cells: NK cells, NK1.1^+^CD3^−^; B cells, B220^+^; T cells, CD3^+^; CD4 T cells, CD3^+^CD4^+^; CD8 T cells, CD3^+^CD8^+^; NK T cells, CD3^+^NK1.1^+^; γδ T cells, CD3^+^CD4^−^CD8^−^NK1.1^−^; microglia/differentiated monocytes, CD11b^+^Ly6C^−^; monocytes, CD11b^+^Ly6C^+^. Also, from gated CD3^+^CD4^+^ or CD3^+^CD8^+^: Tcm, CD44^+^CD62L^+^; and Tem, CD44^+^CD62L^−^.

### Immunohistochemistry

Brains from infected mice were harvested and fixed with 4% paraformaldehyde (PFA) for 48 h then soaked in 18% sucrose overnight at 4°C. Brains were embedded in OCT compound (Fisher Healthcare), flash frozen on dry ice, and 16‐μm sections were cut using a Leica CM1850 UC Cryostat (Leica Biosystems). Brain sections were treated with 2% PFA for 5 min at room temperature and blocked with 10% goat serum for 1 h at room temperature. To detect ABLV‐luc or CVS‐11, sections were stained with mouse anti‐rabies G (clone 1C5; 1:1,000; Novus Biologicals Cat#NBP2‐11630) overnight at 4°C, followed by washing and secondary staining with Alexa Fluor 647 goat anti‐mouse IgG2a (Thermo Fisher Cat#A‐21241; 1:1,000) for 1 h at room temperature. Sections were also co‐stained with DAPI (0.5 μg/ml; Thermo Fisher Cat#D1306) or Alexa Fluor 555 mouse anti‐neuronal nuclei (clone A60; 1:1,000; Millipore Sigma Cat#MAB377A5). To detect the presence of T cell lymphocytes within the CNS, sections were incubated with 2% PFA for 15 min at room temperature and blocked with 10% FBS for 1 h at room temperature. The sections were then stained with Alexa Fluor 594 anti‐mouse CD3 (clone 17A2; 1:100; BioLegend Cat #100240) and Alexa Fluor 647 anti‐mouse CD4 (clone RM4‐5; 1:100; BD Pharmingen Cat #557681) overnight at 4°C, followed by counterstaining with DAPI for 5 min at room temperature. Images were obtained on a Zeiss Axio Scan.Z1 slide scanner and Zen software. For counting T cells in brain sections, three sections per animal of 170 mm^2^ were selected, and counted in a blinded manner, with brain sections from each group randomized to prevent bias. T cells were scored as DAPI‐stained nuclei surrounded by a complete or nearly complete circle of CD3 or CD3 and CD4 staining. The number of CD4‐pos (CD3^+^CD4^+^) and CD4‐neg (CD3^+^CD4^−^) T cells were totaled from the three sections counted for each animal. For detection of F11 in brain tissue, sections were prepared as described above and stained with Alexa Fluor 647 anti‐human IgG (clone QA19A42; 1:50; BioLegend, cat #366913) overnight at 4°C followed by counterstaining with DAPI for 5 min at room temperature.

### Confocal microscopy

Confocal images were obtained with a 40× 1.4NA oil objective, a Zeiss 710 confocal microscope, and Zen software. Contrast levels were adjusted in Zen and images were exported to Adobe Photoshop for cropping.

### qRT‐PCR and RT‐PCR

C57BL/6J mice were infected with ABLV‐luc and treated with F11 as described above. Animals in the control, infected/untreated and infected/F11‐treated groups were euthanized at various times post‐infection. The majority of these mice were analyzed by bioluminescence imaging immediately prior to euthanasia. Brains were collected post‐euthanasia, and one hemisphere was processed for total RNA using Trizol (Invitrogen, Cat# 15596026) and a bead homogenizer (Bullet Blender 5 Gold, Next Advance Laboratory Instruments).

To generate a set of standards for qRT‐PCR, we added defined quantities of linear ABLV‐*N* or ABLV‐*M‐G* DNA molecules to 1 μg of total RNA purified from brains harvested from uninfected animals. Numbers of copies of ABLV plasmid DNA fragments were determined using the formula: (X ng × 6.0221 × 10^23^ molecules/mole)/((Y bp × 660 g/mole) × 1 × 10^9^ ng/g). Analysis of these standards by RT‐PCR demonstrated that there is a linear relationship between log copy number and cycle number from 10^2^ to 10^6^ copies of ABLV‐*N* and ABLV‐*M‐G*, meaning that we could reliably detect as few as 100 copies of positive‐sense transcripts incorporating ABLV‐*N* sequences and negative‐sense ABLV genome sequences per μg of total brain RNA. To quantitatively measure these transcripts in brains of ABLV‐infected animals, we employed a strategy analogous to that recently described to discriminate positive‐strand transcripts from negative‐strand genomes in a Nipah virus model (Jensen *et al*, 2018). Specifically, we prepared cDNA using specific primers to reverse‐transcribe positive‐sense *N* transcripts (5′‐GCCTCCCAAAACAGACATCT‐3′) and negative sense genomes (5′‐TGCCATACAATCGCTGCATTT‐3′). TaqMan real‐time PCR primer sets were designed using the IDT RealTime qPCR Assay design tool. For *N*, primers were sense, 5′‐TCAGATCTCTAGGACTTAGCGG‐3′; antisense, 5′‐CACAGGTGGATATGACAGTCG‐3′; probe, 5′‐TCGTGGGATGTTACATGGGCCAG‐3′. For the *M‐G* region of genomes, primers were sense, 5′‐TGTGAAACCTCCCAGATGTG‐3′; antisense, 5′‐CAATTTGAAGGAGCATACGCG‐3′; probe, 5′‐ACACCCCTCGAACATTCTGAGAAAGAC‐3′.

For measurement of the *Ifng* and *Prf1* mRNAs by RT‐PCR, cDNA was primed with random hexamers. RT‐PCR was performed as described above, except that the ΔΔCT method (Livak & Schmittgen, 2001) was employed to determine fold‐increase in expression relative to uninfected control animals. For the *Ifng* mRNA, primers were sense, 5′‐GAGTATTGCCAAGTTTGAGGTCAA‐3′; antisense, 5′‐TGGTGGACCACTCGGATGA‐3′; probe, 5′‐AACCCACAGGTCCAGCGCCAAG‐3′. For the *Pfr1* mRNA, primers were sense, 5′‐CAGTAGAGTGTCGCATGTACAG‐3′; antisense, 5′‐GAGATGAGCCTGTGGTAAGC‐3′; probe, 5′‐TCTGTGGAGCTGTTAAAGTTGCGGG‐3′. The *Gapdh* mRNA was used as housekeeping gene control, using the following primers: sense, 5′‐TGTGTCCGTCGTGGATCTGA‐3′; antisense, 5′‐CCTGCTTCACCACCTTCTTGA‐3′; probe, 5′‐CCGCCTGGAGAAACCTGCCAAGTATG‐3′. All primers and probes were purchased from IDT. Probes were labeled with FAM and the ZEN/Iowa Black FQ double‐quencher. Assays were run on a BioRad CFX Connect instrument, and data were collected and quantified using Bio‐Rad CFX Maestro software.

### Quantification and statistical analysis

Graphical data presented throughout are expressed as means ± SEMs. For Appendix Fig S2, *P* values for the difference in mean between isotype and CD8 T cell‐depleted mice were calculated by two‐way ANOVA with Bonferroni's multiple comparison post‐test. Kaplan–Meier survival curves presented throughout were calculated using Graphpad Prism v.7, v.9 or v.10 software, and differences in survival were assessed using the logrank test (with the Tukey–Kramer correction for multiple comparisons, as required). For Figs 6 and EV5D and E, *P* values were calculated using a Student's *t* test or, in cases in which one or both populations had a non‐normal distribution, using a Mann–Whitney test. All graphs were generated using Graphpad Prism v.7, v.9 or v.10.

## Author contributions

**Kate E Mastraccio:** Resources; formal analysis; investigation; visualization; methodology; writing – original draft. **Celeste Huaman:** Resources; formal analysis; investigation; visualization; methodology; writing – review and editing. **Si'Ana A Coggins:** Formal analysis; investigation. **Caitlyn Clouse:** Investigation. **Madeline Rader:** Investigation. **Lianying Yan:** Resources. **Pratyusha Mandal:** Investigation. **Imran Hussain:** Resources. **Anwar E Ahmed:** Formal analysis. **Trung Ho:** Resources. **Austin Feasley:** Investigation. **Bang K Vu:** Resources. **Ina L Smith:** Resources. **Wanda Markotter:** Writing – review and editing. **Dawn L Weir:** Resources. **Eric D Laing:** Resources. **Christopher C Broder:** Conceptualization; funding acquisition; writing – review and editing. **Brian C Schaefer:** Conceptualization; formal analysis; funding acquisition; visualization; methodology; writing – original draft.

## Disclosure competing interests statement

The authors declare that they have no conflict of interest.

## For more information

Relevant databases:

- https://ictv.global/report/chapter/rhabdoviridae/rhabdoviridae/lyssavirus
- https://www.who.int/news‐room/fact‐sheets/detail/rabies
- https://www.woah.org/app/uploads/2018/06/zero‐by‐30‐final‐online‐version.pdf

Author websites:

- https://www.researchgate.net/profile/Brian‐Schaefer‐5
- https://www.researchgate.net/profile/Christopher‐Broder

## Supporting information



Appendix

Expanded View Figures PDF

PDF+

Source Data for Expanded View

Source Data for Figure 2

Source Data for Figure 7

## Data Availability

This study includes no data deposited in external repositories.

## References

[emmm202216394-bib-0001] Afrough B , Dowall S , Hewson R (2019) Emerging viruses and current strategies for vaccine intervention. Clin Exp Immunol 196: 157–166 30993690 10.1111/cei.13295 PMC6468171

[emmm202216394-bib-0002] Akilesh S , Christianson GJ , Roopenian DC , Shaw AS (2007) Neonatal FcR expression in bone marrow‐derived cells functions to protect serum IgG from catabolism. J Immunol 179: 4580–4588 17878355 10.4049/jimmunol.179.7.4580

[emmm202216394-bib-0003] Albertini AA , Ruigrok RW , Blondel D (2011) Rabies virus transcription and replication. Adv Virus Res 79: 1–22 21601039 10.1016/B978-0-12-387040-7.00001-9

[emmm202216394-bib-0004] Allworth A , Murray K , Morgan J (1996) A human case of encephalitis due to a lyssavirus recently identified in fruit bats. Commun Dis Intell 20: 504

[emmm202216394-bib-0005] Andrews NP , Pack CD , Lukacher AE (2008) Generation of antiviral major histocompatibility complex class I‐restricted T cells in the absence of CD8 coreceptors. J Virol 82: 4697–4705 18337581 10.1128/JVI.02698-07 PMC2346753

[emmm202216394-bib-0006] Arima Y , Kamimura D , Sabharwal L , Yamada M , Bando H , Ogura H , Atsumi T , Murakami M (2013) Regulation of immune cell infiltration into the CNS by regional neural inputs explained by the gate theory. Mediators Inflamm 2013: 898165 23990699 10.1155/2013/898165 PMC3748732

[emmm202216394-bib-0007] Barouch DH , Whitney JB , Moldt B , Klein F , Oliveira TY , Liu J , Stephenson KE , Chang HW , Shekhar K , Gupta S *et al* (2013) Therapeutic efficacy of potent neutralizing HIV‐1‐specific monoclonal antibodies in SHIV‐infected rhesus monkeys. Nature 503: 224–228 24172905 10.1038/nature12744 PMC4017780

[emmm202216394-bib-0008] Billioux BJ , Nath A , Stavale EJ , Dorbor J , Fallah MP , Sneller MC , Smith BR , Partnership for Research on Ebola Virus in Liberia IIISG (2017) Cerebrospinal fluid examination in survivors of Ebola virus disease. JAMA Neurol 74: 1141–1143 28715541 10.1001/jamaneurol.2017.1460 PMC5822214

[emmm202216394-bib-0009] Bongiorno EK , Garcia SA , Sauma S , Hooper DC (2017) Type 1 immune mechanisms driven by the response to infection with attenuated rabies virus result in changes in the immune bias of the tumor microenvironment and necrosis of mouse GL261 brain tumors. J Immunol 198: 4513–4523 28461570 10.4049/jimmunol.1601444 PMC5467701

[emmm202216394-bib-0010] Both L , Banyard AC , van Dolleweerd C , Horton DL , Ma JK , Fooks AR (2012) Passive immunity in the prevention of rabies. Lancet Infect Dis 12: 397–407 22541629 10.1016/S1473-3099(11)70340-1

[emmm202216394-bib-0011] Caviness K , Kuhn JH , Palacios G (2017) Ebola virus persistence as a new focus in clinical research. Curr Opin Virol 23: 43–48 28340374 10.1016/j.coviro.2017.02.006

[emmm202216394-bib-0012] Charlton KM , Nadin‐Davis S , Casey GA , Wandeler AI (1997) The long incubation period in rabies: delayed progression of infection in muscle at the site of exposure. Acta Neuropathol 94: 73–77 9224533 10.1007/s004010050674

[emmm202216394-bib-0013] Chughtai AA , Barnes M , Macintyre CR (2016) Persistence of Ebola virus in various body fluids during convalescence: evidence and implications for disease transmission and control. Epidemiol Infect 144: 1652–1660 26808232 10.1017/S0950268816000054 PMC4855994

[emmm202216394-bib-0014] Dalloul AH , Ngo K , Fung‐Leung WP (1996) CD4‐negative cytotoxic T cells with a T cell receptor alpha/beta intermediate expression in CD8‐deficient mice. Eur J Immunol 26: 213–218 8566069 10.1002/eji.1830260133

[emmm202216394-bib-0015] Davison AJ , Siddell S , Simmonds P (2017–2020) Online report of the International Committee on Taxonomy of Viruses. In Virus taxonomy: classification and nomenclature of viruses, Lefkowitz EJ (ed). Amsterdam: Elsevier

[emmm202216394-bib-0016] Dawes BE , Freiberg AN (2019) Henipavirus infection of the central nervous system. Pathog Dis 77: ftz023 30985897 10.1093/femspd/ftz023 PMC6974701

[emmm202216394-bib-0017] De Benedictis P , Minola A , Rota Nodari E , Aiello R , Zecchin B , Salomoni A , Foglierini M , Agatic G , Vanzetta F , Lavenir R *et al* (2016) Development of broad‐spectrum human monoclonal antibodies for rabies post‐exposure prophylaxis. EMBO Mol Med 8: 407–421 26992832 10.15252/emmm.201505986 PMC4818751

[emmm202216394-bib-0018] Dietzschold B , Gore M , Casali P , Ueki Y , Rupprecht CE , Notkins AL , Koprowski H (1990) Biological characterization of human monoclonal antibodies to rabies virus. J Virol 64: 3087–3090 2335829 10.1128/jvi.64.6.3087-3090.1990 PMC249498

[emmm202216394-bib-0019] Dietzschold B , Kao M , Zheng YM , Chen ZY , Maul G , Fu ZF , Rupprecht CE , Koprowski H (1992) Delineation of putative mechanisms involved in antibody‐mediated clearance of rabies virus from the central nervous system. Proc Natl Acad Sci U S A 89: 7252–7256 1496020 10.1073/pnas.89.15.7252 PMC49684

[emmm202216394-bib-0020] Dietzschold B , Li J , Faber M , Schnell M (2008) Concepts in the pathogenesis of rabies. Future Virol 3: 481–490 19578477 10.2217/17460794.3.5.481 PMC2600441

[emmm202216394-bib-0021] DiLillo DJ , Ravetch JV (2015) Fc‐receptor interactions regulate both cytotoxic and immunomodulatory therapeutic antibody effector functions. Cancer Immunol Res 3: 704–713 26138698 10.1158/2326-6066.CIR-15-0120

[emmm202216394-bib-0022] Earnest JT , Basore K , Roy V , Bailey AL , Wang D , Alter G , Fremont DH , Diamond MS (2019) Neutralizing antibodies against Mayaro virus require Fc effector functions for protective activity. J Exp Med 216: 2282–2301 31337735 10.1084/jem.20190736 PMC6781005

[emmm202216394-bib-0023] Engelhardt B (2006) Regulation of immune cell entry into the central nervous system. Results Probl Cell Differ 43: 259–280 17068976 10.1007/400_020

[emmm202216394-bib-0024] Fabis MJ , Phares TW , Kean RB , Koprowski H , Hooper DC (2008) Blood‐brain barrier changes and cell invasion differ between therapeutic immune clearance of neurotrophic virus and CNS autoimmunity. Proc Natl Acad Sci U S A 105: 15511–15516 18829442 10.1073/pnas.0807656105 PMC2563072

[emmm202216394-bib-0025] Feige L , Kozaki T , Dias de Melo G , Guillemot V , Larrous F , Ginhoux F , Bourhy H (2022) Susceptibilities of CNS cells towards rabies virus infection is linked to cellular innate immune responses. Viruses 15: 88 36680128 10.3390/v15010088 PMC9860954

[emmm202216394-bib-0026] Finke S , Conzelmann KK (1997) Ambisense gene expression from recombinant rabies virus: random packaging of positive‐ and negative‐strand ribonucleoprotein complexes into rabies virions. J Virol 71: 7281–7288 9311803 10.1128/jvi.71.10.7281-7288.1997 PMC192070

[emmm202216394-bib-0027] Fox JM , Roy V , Gunn BM , Huang L , Edeling MA , Mack M , Fremont DH , Doranz BJ , Johnson S , Alter G *et al* (2019) Optimal therapeutic activity of monoclonal antibodies against chikungunya virus requires Fc‐FcgammaR interaction on monocytes. Sci Immunol 4: eaav5062 30796092 10.1126/sciimmunol.aav5062 PMC6698136

[emmm202216394-bib-0028] Francis JR , Nourse C , Vaska VL , Calvert S , Northill JA , McCall B , Mattke AC (2014) Australian Bat Lyssavirus in a child: the first reported case. Pediatrics 133: e1063–e1067 24590754 10.1542/peds.2013-1782

[emmm202216394-bib-0029] Franka R , Wu X , Jackson FR , Velasco‐Villa A , Palmer DP , Henderson H , Hayat W , Green DB , Blanton JD , Greenberg L *et al* (2009) Rabies virus pathogenesis in relationship to intervention with inactivated and attenuated rabies vaccines. Vaccine 27: 7149–7155 19925945 10.1016/j.vaccine.2009.09.034

[emmm202216394-bib-0030] Garcia SA , Lebrun A , Kean RB , Craig Hooper D (2018) Clearance of attenuated rabies virus from brain tissues is required for long‐term protection against CNS challenge with a pathogenic variant. J Neurovirol 24: 606–615 29987584 10.1007/s13365-018-0655-z PMC6246785

[emmm202216394-bib-0031] Ghetie V , Hubbard JG , Kim JK , Tsen MF , Lee Y , Ward ES (1996) Abnormally short serum half‐lives of IgG in beta 2‐microglobulin‐deficient mice. Eur J Immunol 26: 690–696 8605939 10.1002/eji.1830260327

[emmm202216394-bib-0032] Gillet JP , Derer P , Tsiang H (1986) Axonal transport of rabies virus in the central nervous system of the rat. J Neuropathol Exp Neurol 45: 619–634 2430067 10.1097/00005072-198611000-00002

[emmm202216394-bib-0033] Gould AR , Hyatt AD , Lunt R , Kattenbelt JA , Hengstberger S , Blacksell SD (1998) Characterisation of a novel lyssavirus isolated from Pteropid bats in Australia. Virus Res 54: 165–187 9696125 10.1016/s0168-1702(98)00025-2

[emmm202216394-bib-0034] Greter M , Lelios I , Croxford AL (2015) Microglia versus myeloid cell nomenclature during brain inflammation. Front Immunol 6: 249 26074918 10.3389/fimmu.2015.00249 PMC4443742

[emmm202216394-bib-0035] Guldner IH , Golomb SM , Wang Q , Wang E , Zhang S (2021) Isolation of mouse brain‐infiltrating leukocytes for single cell profiling of epitopes and transcriptomes. STAR Protoc 2: 100537 34036283 10.1016/j.xpro.2021.100537 PMC8138863

[emmm202216394-bib-0036] Hanlon CA , Smith JS , Anderson GR (1999) Recommendations of a national working group on prevention and control of rabies in the United States. Article II: Laboratory diagnosis of rabies. The National Working Group on Rabies Prevention and Control. J Am Vet Med Assoc 215: 1444–1446 10579039

[emmm202216394-bib-0037] Hanna JN , Carney IK , Smith GA , Tannenberg AE , Deverill JE , Botha JA , Serafin IL , Harrower BJ , Fitzpatrick PF , Searle JW (2000) Australian bat lyssavirus infection: a second human case, with a long incubation period. Med J Aust 172: 597–599 10914106 10.5694/j.1326-5377.2000.tb124126.x

[emmm202216394-bib-0038] Hooper DC , Morimoto K , Bette M , Weihe E , Koprowski H , Dietzschold B (1998) Collaboration of antibody and inflammation in clearance of rabies virus from the central nervous system. J Virol 72: 3711–3719 9557653 10.1128/jvi.72.5.3711-3719.1998 PMC109593

[emmm202216394-bib-0039] Hooper DC , Phares TW , Fabis MJ , Roy A (2009) The production of antibody by invading B cells is required for the clearance of rabies virus from the central nervous system. PLoS Negl Trop Dis 3: e535 19806203 10.1371/journal.pntd.0000535 PMC2754506

[emmm202216394-bib-0040] Horton DL , McElhinney LM , Marston DA , Wood JL , Russell CA , Lewis N , Kuzmin IV , Fouchier RA , Osterhaus AD , Fooks AR *et al* (2010) Quantifying antigenic relationships among the lyssaviruses. J Virol 84: 11841–11848 20826698 10.1128/JVI.01153-10 PMC2977894

[emmm202216394-bib-0041] Howlett PJ , Walder AR , Lisk DR , Fitzgerald F , Sevalie S , Lado M , N'Jai A , Brown CS , Sahr F , Sesay F *et al* (2018) Case series of severe neurologic sequelae of Ebola virus disease during epidemic, Sierra Leone. Emerg Infect Dis 24: 1412–1421 30014839 10.3201/eid2408.171367 PMC6056101

[emmm202216394-bib-0042] Iversen PL , Kane CD , Zeng X , Panchal RG , Warren TK , Radoshitzky SR , Kuhn JH , Mudhasani RR , Cooper CL , Shurtleff AC *et al* (2020) Recent successes in therapeutics for Ebola virus disease: no time for complacency. Lancet Infect Dis 20: e231–e237 32563280 10.1016/S1473-3099(20)30282-6 PMC7302789

[emmm202216394-bib-0043] Jensen KS , Adams R , Bennett RS , Bernbaum J , Jahrling PB , Holbrook MR (2018) Development of a novel real‐time polymerase chain reaction assay for the quantitative detection of Nipah virus replicative viral RNA. PloS One 13: e0199534 29920552 10.1371/journal.pone.0199534 PMC6007899

[emmm202216394-bib-0044] Junghans RP , Anderson CL (1996) The protection receptor for IgG catabolism is the beta2‐microglobulin‐containing neonatal intestinal transport receptor. Proc Natl Acad Sci U S A 93: 5512–5516 8643606 10.1073/pnas.93.11.5512 PMC39277

[emmm202216394-bib-0045] Keeler SP , Fox JM (2021) Requirement of Fc‐Fc gamma receptor interaction for antibody‐based protection against emerging virus infections. Viruses 13: 1037 34072720 10.3390/v13061037 PMC8226613

[emmm202216394-bib-0046] de Kruif J , Bakker AB , Marissen WE , Kramer RA , Throsby M , Rupprecht CE , Goudsmit J (2007) A human monoclonal antibody cocktail as a novel component of rabies postexposure prophylaxis. Annu Rev Med 58: 359–368 16886905 10.1146/annurev.med.58.061705.145053

[emmm202216394-bib-0047] Lafon M (2011) Evasive strategies in rabies virus infection. Adv Virus Res 79: 33–53 21601041 10.1016/B978-0-12-387040-7.00003-2

[emmm202216394-bib-0048] Lebrun A , Portocarrero C , Kean RB , Barkhouse DA , Faber M , Hooper DC (2015) T‐bet is required for the rapid clearance of attenuated rabies virus from central nervous system tissue. J Immunol 195: 4358–4368 26408670 10.4049/jimmunol.1501274 PMC4610868

[emmm202216394-bib-0049] Livak KJ , Schmittgen TD (2001) Analysis of relative gene expression data using real‐time quantitative PCR and the 2(‐Delta Delta C(T)) Method. Methods 25: 402–408 11846609 10.1006/meth.2001.1262

[emmm202216394-bib-0050] Lo M , Kim HS , Tong RK , Bainbridge TW , Vernes JM , Zhang Y , Lin YL , Chung S , Dennis MS , Zuchero YJ *et al* (2017) Effector‐attenuating substitutions that maintain antibody stability and reduce toxicity in mice. J Biol Chem 292: 3900–3908 28077575 10.1074/jbc.M116.767749 PMC5339770

[emmm202216394-bib-0051] Ludlow M , Kortekaas J , Herden C , Hoffmann B , Tappe D , Trebst C , Griffin DE , Brindle HE , Solomon T , Brown AS *et al* (2016) Neurotropic virus infections as the cause of immediate and delayed neuropathology. Acta Neuropathol 131: 159–184 26659576 10.1007/s00401-015-1511-3 PMC4713712

[emmm202216394-bib-0052] Manning SE , Rupprecht CE , Fishbein D , Hanlon CA , Lumlertdacha B , Guerra M , Meltzer MI , Dhankhar P , Vaidya SA , Jenkins SR *et al* (2008) Human rabies prevention–United States, 2008: recommendations of the Advisory Committee on Immunization Practices. MMWR Recomm Rep 57: 1–28 18496505

[emmm202216394-bib-0053] Markotter W , Coertse J (2018) Bat lyssaviruses. Rev Sci Tech 37: 385–400 30747140 10.20506/rst.37.2.2809

[emmm202216394-bib-0054] Mastraccio KE , Huaman C , Warrilow D , Smith GA , Craig SB , Weir DL , Laing ED , Smith IL , Broder CC , Schaefer BC (2020) Establishment of a longitudinal pre‐clinical model of lyssavirus infection. J Virol Methods 281: 113882 32407866 10.1016/j.jviromet.2020.113882 PMC8056983

[emmm202216394-bib-0055] McCarthy MK , Morrison TE (2017) Persistent RNA virus infections: do PAMPS drive chronic disease? Curr Opin Virol 23: 8–15 28214732 10.1016/j.coviro.2017.01.003 PMC5474173

[emmm202216394-bib-0056] de Melo GD , Sonthonnax F , Lepousez G , Jouvion G , Minola A , Zatta F , Larrous F , Kergoat L , Mazo C , Moigneu C *et al* (2020) A combination of two human monoclonal antibodies cures symptomatic rabies. EMBO Mol Med 12: e12628 32945125 10.15252/emmm.202012628 PMC7645379

[emmm202216394-bib-0057] Nimmerjahn F , Ravetch JV (2007) Fc‐receptors as regulators of immunity. Adv Immunol 96: 179–204 17981207 10.1016/S0065-2776(07)96005-8

[emmm202216394-bib-0058] Olival KJ , Daszak P (2005) The ecology of emerging neurotropic viruses. J Neurovirol 11: 441–446 16287685 10.1080/13550280591002450

[emmm202216394-bib-0059] Overdijk MB , Verploegen S , Ortiz Buijsse A , Vink T , Leusen JH , Bleeker WK , Parren PW (2012) Crosstalk between human IgG isotypes and murine effector cells. J Immunol 189: 3430–3438 22956577 10.4049/jimmunol.1200356

[emmm202216394-bib-0060] Phares TW , Fabis MJ , Brimer CM , Kean RB , Hooper DC (2007) A peroxynitrite‐dependent pathway is responsible for blood‐brain barrier permeability changes during a central nervous system inflammatory response: TNF‐alpha is neither necessary nor sufficient. J Immunol 178: 7334–7343 17513784 10.4049/jimmunol.178.11.7334

[emmm202216394-bib-0061] Playford EG , Munro T , Mahler SM , Elliott S , Gerometta M , Hoger KL , Jones ML , Griffin P , Lynch KD , Carroll H *et al* (2020) Safety, tolerability, pharmacokinetics, and immunogenicity of a human monoclonal antibody targeting the G glycoprotein of henipaviruses in healthy adults: a first‐in‐human, randomised, controlled, phase 1 study. Lancet Infect Dis 20: 445–454 32027842 10.1016/S1473-3099(19)30634-6

[emmm202216394-bib-0062] Prosniak M , Faber M , Hanlon CA , Rupprecht CE , Hooper DC , Dietzschold B (2003) Development of a cocktail of recombinant‐expressed human rabies virus‐neutralizing monoclonal antibodies for postexposure prophylaxis of rabies. J Infect Dis 188: 53–56 12825170 10.1086/375247

[emmm202216394-bib-0063] Roopenian DC , Akilesh S (2007) FcRn: the neonatal Fc receptor comes of age. Nat Rev Immunol 7: 715–725 17703228 10.1038/nri2155

[emmm202216394-bib-0064] Rupprecht CE , Nagarajan T , Ertl H (2016) Current status and development of vaccines and other biologics for human rabies prevention. Expert Rev Vaccines 15: 731–749 26796599 10.1586/14760584.2016.1140040

[emmm202216394-bib-0065] Sagui E , Janvier F , Baize S , Foissaud V , Koulibaly F , Savini H , Maugey N , Aletti M , Granier H , Carmoi T (2015) Severe Ebola virus infection with encephalopathy: evidence for direct virus involvement. Clin Infect Dis 61: 1627–1628 26197842 10.1093/cid/civ606

[emmm202216394-bib-0066] Samaranayake H , Wirth T , Schenkwein D , Raty JK , Yla‐Herttuala S (2009) Challenges in monoclonal antibody‐based therapies. Ann Med 41: 322–331 19234897 10.1080/07853890802698842

[emmm202216394-bib-0067] Schroder K , Hertzog PJ , Ravasi T , Hume DA (2004) Interferon‐gamma: an overview of signals, mechanisms and functions. J Leukoc Biol 75: 163–189 14525967 10.1189/jlb.0603252

[emmm202216394-bib-0068] Schumacher CL , Dietzschold B , Ertl HC , Niu HS , Rupprecht CE , Koprowski H (1989) Use of mouse anti‐rabies monoclonal antibodies in postexposure treatment of rabies. J Clin Invest 84: 971–975 2760222 10.1172/JCI114260 PMC329743

[emmm202216394-bib-0069] Shingai M , Nishimura Y , Klein F , Mouquet H , Donau OK , Plishka R , Buckler‐White A , Seaman M , Piatak M Jr , Lifson JD *et al* (2013) Antibody‐mediated immunotherapy of macaques chronically infected with SHIV suppresses viraemia. Nature 503: 277–280 24172896 10.1038/nature12746 PMC4133787

[emmm202216394-bib-0070] Smith SP , Wu G , Fooks AR , Ma J , Banyard AC (2019) Trying to treat the untreatable: experimental approaches to clear rabies virus infection from the CNS. J Gen Virol 100: 1171–1186 31237530 10.1099/jgv.0.001269

[emmm202216394-bib-0071] Steimle V , Siegrist CA , Mottet A , Lisowska‐Grospierre B , Mach B (1994) Regulation of MHC class II expression by interferon‐gamma mediated by the transactivator gene CIITA. Science 265: 106–109 8016643 10.1126/science.8016643

[emmm202216394-bib-0072] Studahl M , Lindquist L , Eriksson BM , Gunther G , Bengner M , Franzen‐Rohl E , Fohlman J , Bergstrom T , Aurelius E (2013) Acute viral infections of the central nervous system in immunocompetent adults: diagnosis and management. Drugs 73: 131–158 23377760 10.1007/s40265-013-0007-5

[emmm202216394-bib-0073] Tan CT , Goh KJ , Wong KT , Sarji SA , Chua KB , Chew NK , Murugasu P , Loh YL , Chong HT , Tan KS *et al* (2002) Relapsed and late‐onset Nipah encephalitis. Ann Neurol 51: 703–708 12112075 10.1002/ana.10212

[emmm202216394-bib-0074] Ullah I , Prevost J , Ladinsky MS , Stone H , Lu M , Anand SP , Beaudoin‐Bussieres G , Symmes K , Benlarbi M , Ding S *et al* (2021) Live imaging of SARS‐CoV‐2 infection in mice reveals that neutralizing antibodies require Fc function for optimal efficacy. Immunity 54: 2143–2158 34453881 10.1016/j.immuni.2021.08.015 PMC8372518

[emmm202216394-bib-0075] Urban SL , Jensen IJ , Shan Q , Pewe LL , Xue HH , Badovinac VP , Harty JT (2020) Peripherally induced brain tissue‐resident memory CD8(+) T cells mediate protection against CNS infection. Nat Immunol 21: 938–949 32572242 10.1038/s41590-020-0711-8 PMC7381383

[emmm202216394-bib-0076] Velandia‐Romero ML , Castellanos JE , Martinez‐Gutierrez M (2013) In vivo differential susceptibility of sensory neurons to rabies virus infection. J Neurovirol 10.1007/s13365-013-0179-5 23959650

[emmm202216394-bib-0077] Walker LM , Burton DR (2018) Passive immunotherapy of viral infections: ‘super‐antibodies’ enter the fray. Nat Rev Immunol 18: 297–308 29379211 10.1038/nri.2017.148 PMC5918154

[emmm202216394-bib-0078] Walker KW , Salimi‐Moosavi H , Arnold GE , Chen Q , Soto M , Jacobsen FW , Hui J (2019) Pharmacokinetic comparison of a diverse panel of non‐targeting human antibodies as matched IgG1 and IgG2 isotypes in rodents and non‐human primates. PloS One 14: e0217061 31120944 10.1371/journal.pone.0217061 PMC6533040

[emmm202216394-bib-0079] Weir DL , Coggins SA , Vu BK , Coertese J , Yan L , Smith IL , Laing ED , Markotter W , Broder CC , Schaefer BC (2021) Isolation and characterization of cross‐reactive human mono‐clonal antibodies that potently neutralize Australian bat lyssa‐virus variants and other phylogroup 1 lyssaviruses. Viruses 13: 391 33804519 10.3390/v13030391 PMC8001737

[emmm202216394-bib-0080] World Health Organization (2018) Rabies vaccines: WHO position paper, April 2018 – Recommendations. Vaccine 36: 5500–5503 30107991 10.1016/j.vaccine.2018.06.061

[emmm202216394-bib-0081] Wu X , Franka R , Henderson H , Rupprecht CE (2011) Live attenuated rabies virus co‐infected with street rabies virus protects animals against rabies. Vaccine 29: 4195–4201 21514343 10.1016/j.vaccine.2011.03.104

[emmm202216394-bib-0082] Zhu Z , Bossart KN , Bishop KA , Crameri G , Dimitrov AS , McEachern JA , Feng Y , Middleton D , Wang LF , Broder CC *et al* (2008) Exceptionally potent cross-reactive neutralization of Nipah and Hendra viruses by a human monoclonal antibody. J Infect Dis 197: 846–853 18271743 10.1086/528801 PMC7199872

