# Supplementary material for: mAb therapy controls CNS‐resident lyssavirus infection via a CD4 T cell‐dependent mechanism
Source: EMBO Mol Med. 2023 Sep 28;15(10):e16394. doi: 10.15252/emmm.202216394 (PMC10565638; doi:10.15252/emmm.202216394)
Supplement: Supplementary file 1 — Appendix [file EMMM-15-e16394-s003.pdf]

Appendix Table of Contents

Appendix Figure S1. B cells do not play an essential role in F11-dependent control of ABLV infection.

Appendix Figure S2. CD8 T cell depletion efficiency in C57BL/6J mice.

Appendix Figure S3. Bioluminescence imaging of ABLV-luc infection following treatment with mAbs m102.4, F11 and F11(LALA-PG).

Appendix Figure S4. Detection of cell-associated F11 in brains of ABLV-infected, F11-treated animals.

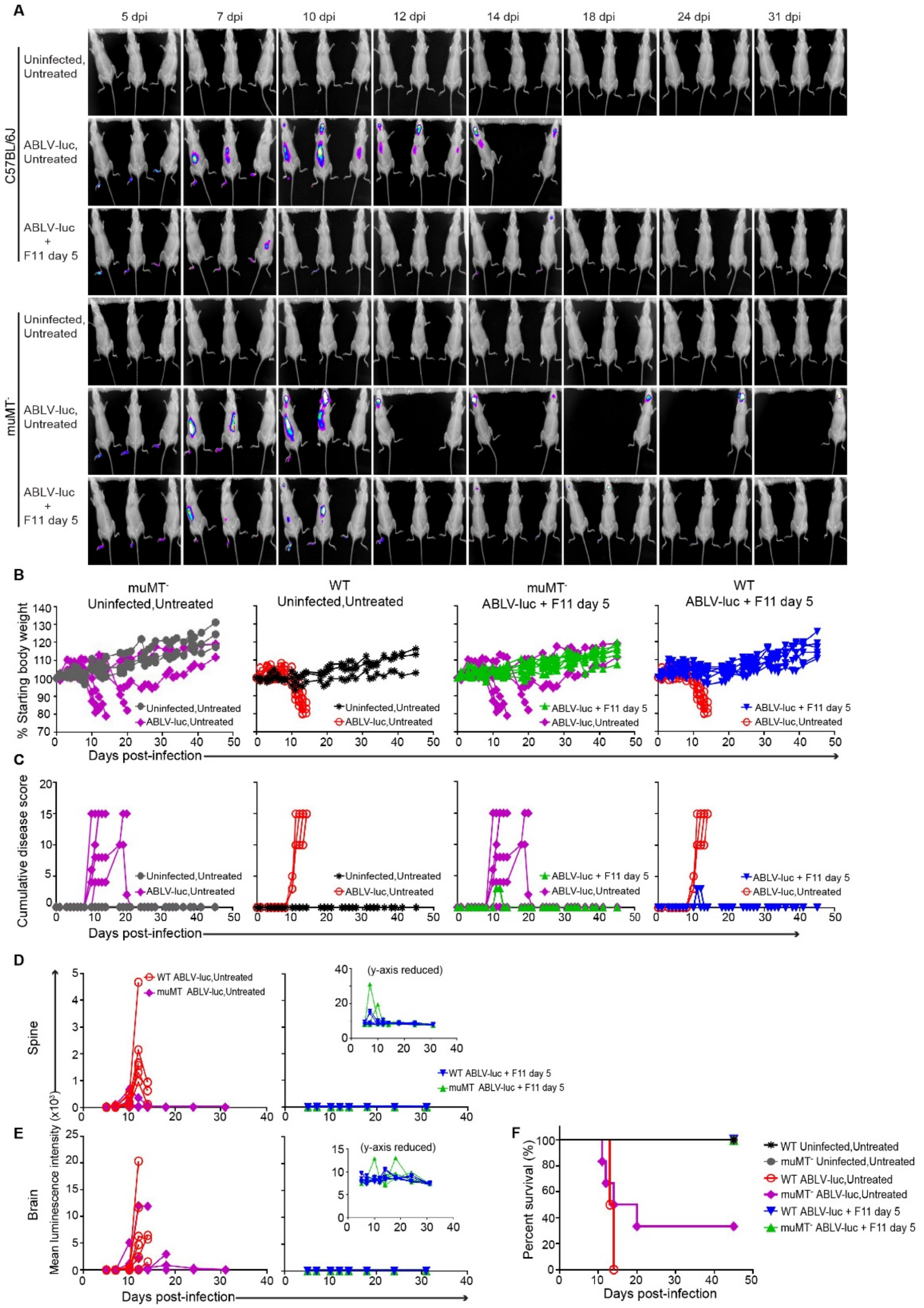

Appendix Figure S1. B cells do not play an essential role in F11-dependent control of ABLV infection. (**A**) Bioluminescence imaging of C57BL/6J and muMT$^-$ mice infected with $2{\times}10^5$ FFU of ABLV-luc on day 0 and treated with mAb F11 on day 5 (n=6 mice/group, n=3 Uninfected, Untreated mice/group). Percent starting body weight (**B**) and cumulative disease scores (**C**) following ABLV-luc challenge and F11 treatment. Viral burden was quantified as mean luminescence intensity (MLI) in the spines (**D**) and brains (**E**) of infected mice. Insets are same data with reduced y-axis scale; note that inset y-axis values are not multiplied by 10^3. (**F**) Kaplan-Meier survival plot. WT, ABLV-luc Untreated vs. WT, ABLV-luc F11, $p = 0.0006$ (raw), $p = 0.0036$ (corrected); muMT-, ABLV-luc Untreated vs. muMT-, ABLV-luc F11, $p = 0.0195$ (raw); $p = 0.09$ (NS) (corrected); WT, ABLV-luc, Untreated vs. muMT-, ABLV-luc Untreated, $p = 0.267$ (NS) (raw), $p = 0.684$ (NS) (corrected). Logrank test with Tukey-Kramer correction for multiple comparisons.

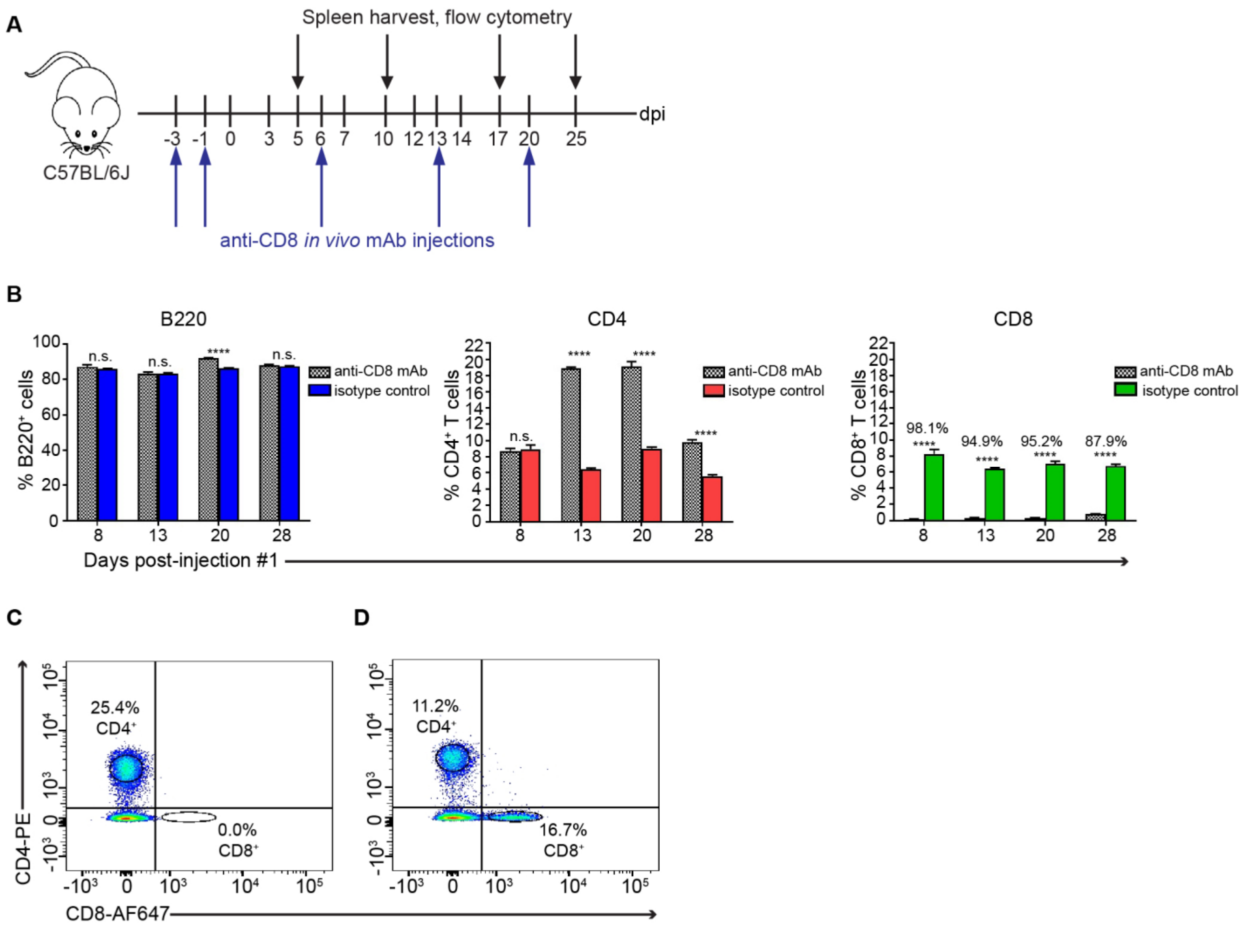

Appendix Figure S2. CD8 T cell depletion efficiency in C57BL/6J mice. (**A**) Anti-CD8α mAb (300 μg) was administered to mice on days -3, -1, 6, 13, and 20 (n=3 mice/group). Spleens were harvested on days 5, 10, 17, and 25 to assess CD8 T cell depletion. (**B**) Values are mean percentage of B220^{+} cells, CD4^{+} T cells, and CD8^{+} T cells on days 8, 13, 20, and 28 following the first dose of anti-CD8α mAb. ****$p < 0.0001$, CD4:****$p < 0.0001$, CD8:****$p < 0.0001$, n.s., not significant, Two-way ANOVA, Bonferroni's multiple comparisons test. Error bars are SEM. Percent CD8 T cell depletion shown above each set of bars in CD8 panel. Representative flow plots from mice that received anti-CD8α mAb (**C**) or isotype control (**D**) on day 5.

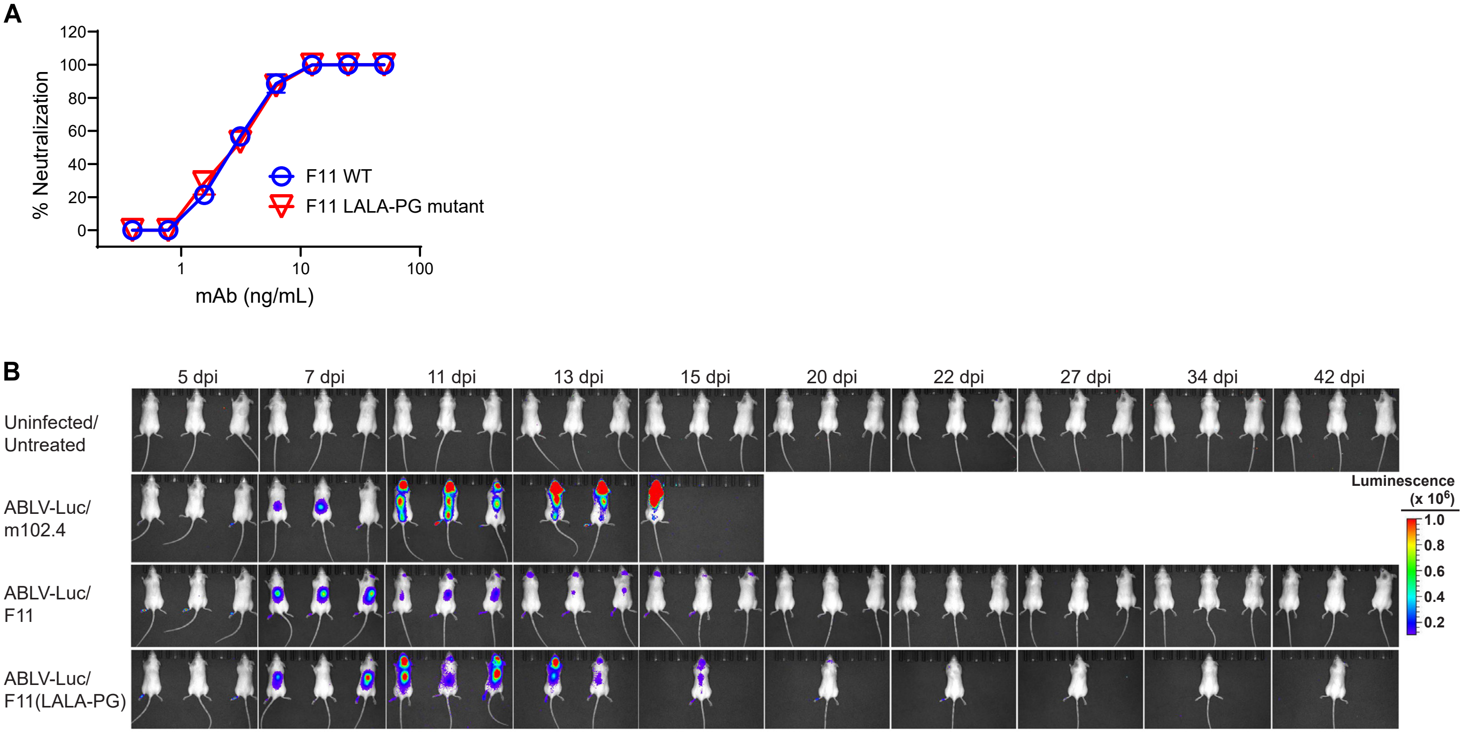

Appendix Figure S3. Bioluminescence imaging of ABLV-luc infection following treatment with mAbs m102.4, F11 and F11(LALA-PG). (**A**) Neutralization assay demonstrating equivalent *in vitro* neutralization of ABLV-luc by F11 and F11(LALA-PG) (n=3 wells/condition). (**B**) Bioluminescence imaging of B6-Albino mice infected with 2×10^5 FFU of ABLV-luc and treated with mAb F11 or F11 (LALA-PG) on day 5. Images of time points shown in Fig 7B are repeated here (n=3 mice/group for uninfected; n=6 mice/group for all others).

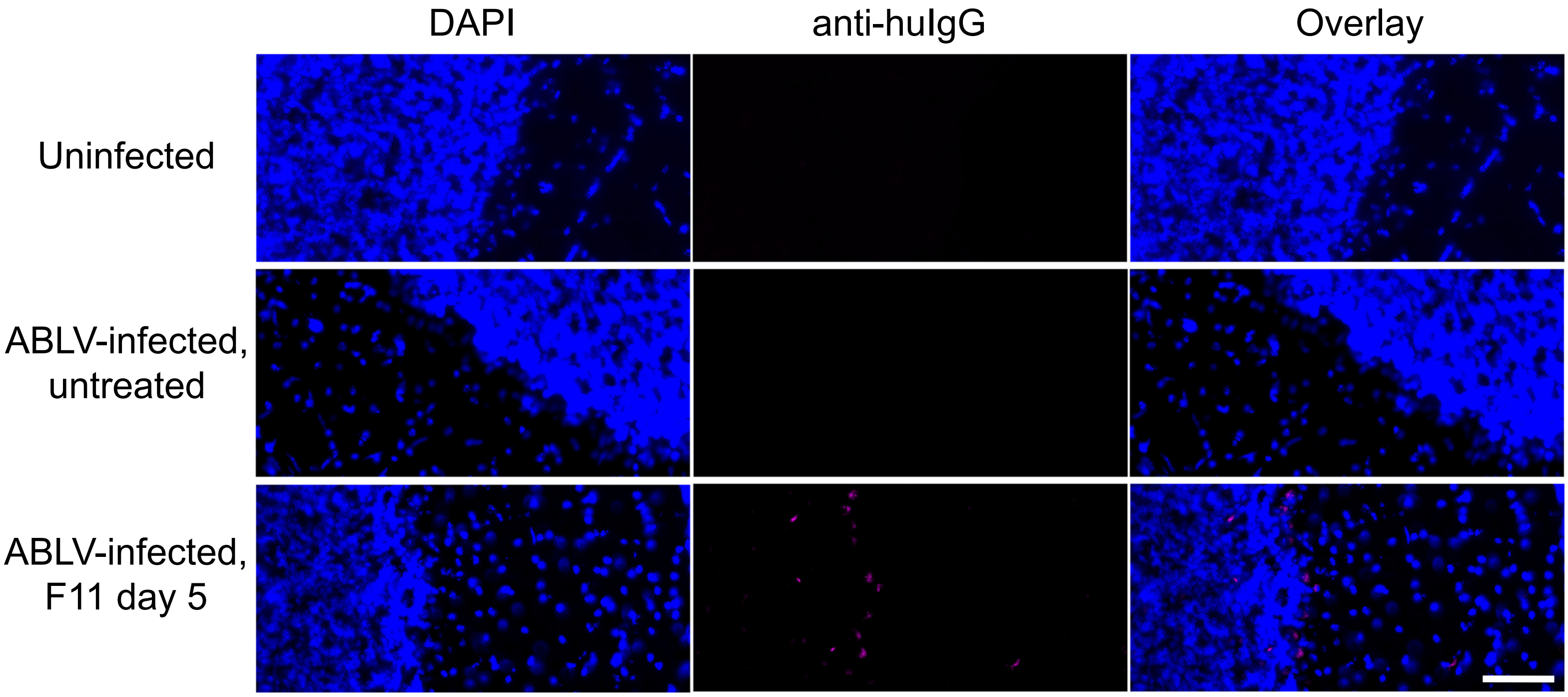

Appendix Figure S4. Detection of cell-associated F11 in brains of ABLV-infected, F11-treated animals. Day 14 post-infection coronal sections of cerebellum from ABLV-infected, F11-treated (day 5) mice were stained with DAPI to detect nuclear DNA and with AlexaFluor 647 anti-human IgG to detect F11. Bar, 50 µm.
