# Supplementary material for: mAb therapy controls CNS‐resident lyssavirus infection via a CD4 T cell‐dependent mechanism
Source: EMBO Mol Med. 2023 Sep 28;15(10):e16394. doi: 10.15252/emmm.202216394 (PMC10565638; doi:10.15252/emmm.202216394)
Supplement: Supplementary file 2 — Expanded View Figures PDF [file EMMM-15-e16394-s001.pdf]

Expanded View Figures

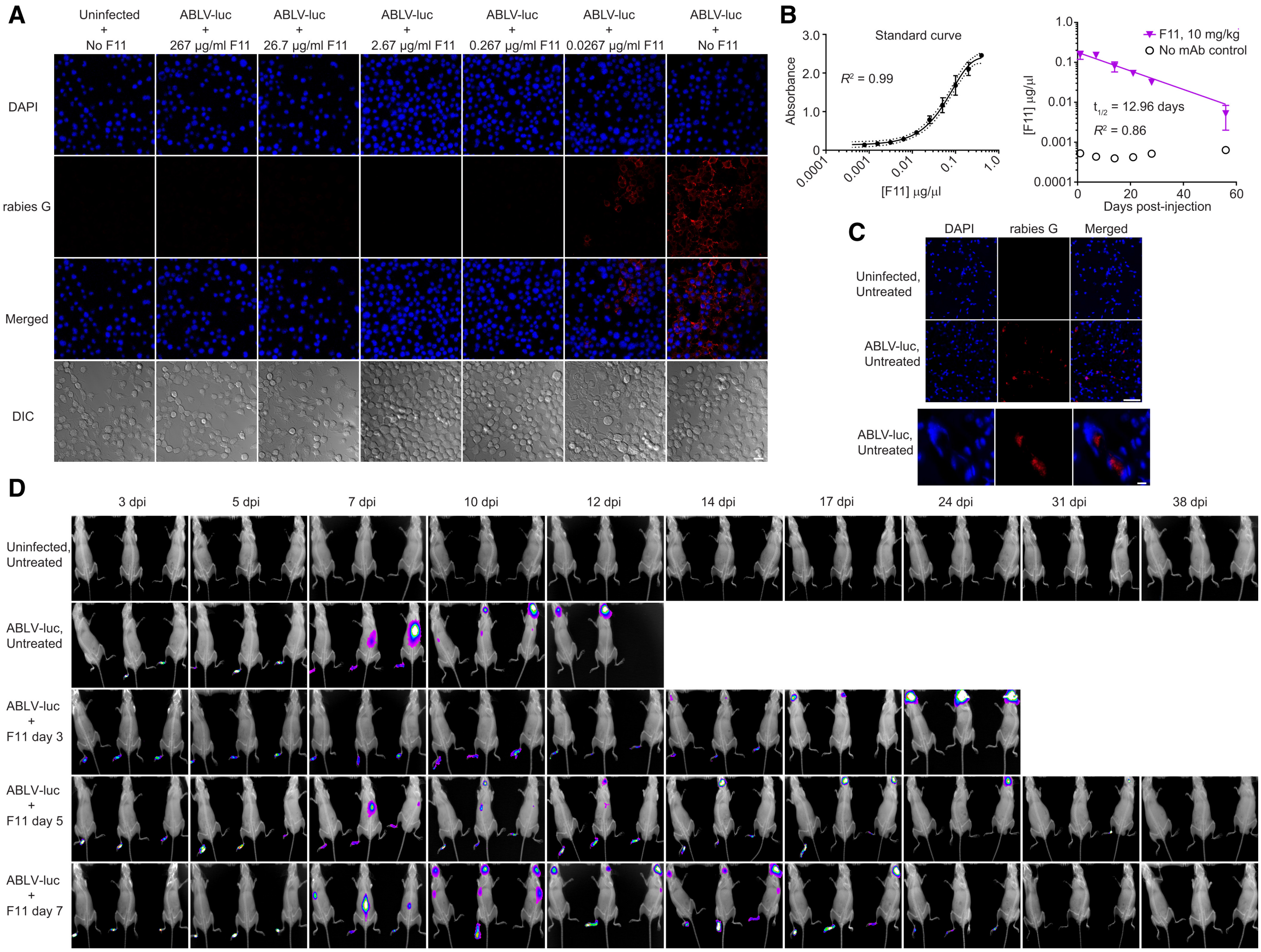

Figure EV1. Therapy with F11 mAb controls ABLV-luc infection in B6 Albino mice.

A Ten-fold serial dilutions of F11 were incubated with 8×10^4 FFU of ABLV-luc, added to mouse N2a cells, and cells were stained with anti-rabies G to detect virions. Bar, 20 µm.

B Left graph is standard curve of absorbance vs. concentration of F11 diluted in normal mouse serum, measured by ELISA. Right graph is average concentration of F11 in serum of mice over time. Mice were injected on day 0 with 10 mg/kg F11. Sera were collected from 4 mice per time point at 1, 7, 14, 21, 28 and 56 days post injection. Concentration was calculated based on standard curve, and F11 *in vivo* half-life was calculated via non-linear regression using one-phase exponential decay with a least squares fit. Error bars are SD.

C Coronal sections of brain stem from ABLV-luc-infected mice (day 11 post-infection) were stained to confirm the presence of virus. Bar, top set 50 µm, bottom set 10 µm.

D Bioluminescence imaging of mice infected with 2×10^5 FFU of ABLV-luc and treated with F11 on day 3, 5, or 7 post-infection.

Source data are available online for this figure.

Figure EV2. Failure of F11 therapy to promote survival in two additional strains of mice lacking adaptive immune cells.

A NOD-SCID and Rag 1KO mice were infected with 2×10^5 FFU of ABLV-luc on day 0 and mAb F11 (10 mg/kg) was administered on day 5 (n = 6 mice/group).

B Bioluminescence imaging of mice was conducted on the indicated days post-infection.

C Viral burden was quantified as mean luminescence intensity in spines and brains of mice.

D Cumulative disease scores were determined by clinical presentation following. ABLV-luc challenge.

E Percent starting body weight over time as an indicator of disease.

F Kaplan–Meier survival plot. NOD-SCID ABLV-luc, Untreated vs. NOD-SCID ABLV-luc, F11, P = 0.025. Rag1 KO ABLV-luc, Untreated vs. Rag1 KO ABLV-luc, F11, P = 0.022. Logrank test.

 © 2023 Commonwealth of Australia and The Authors

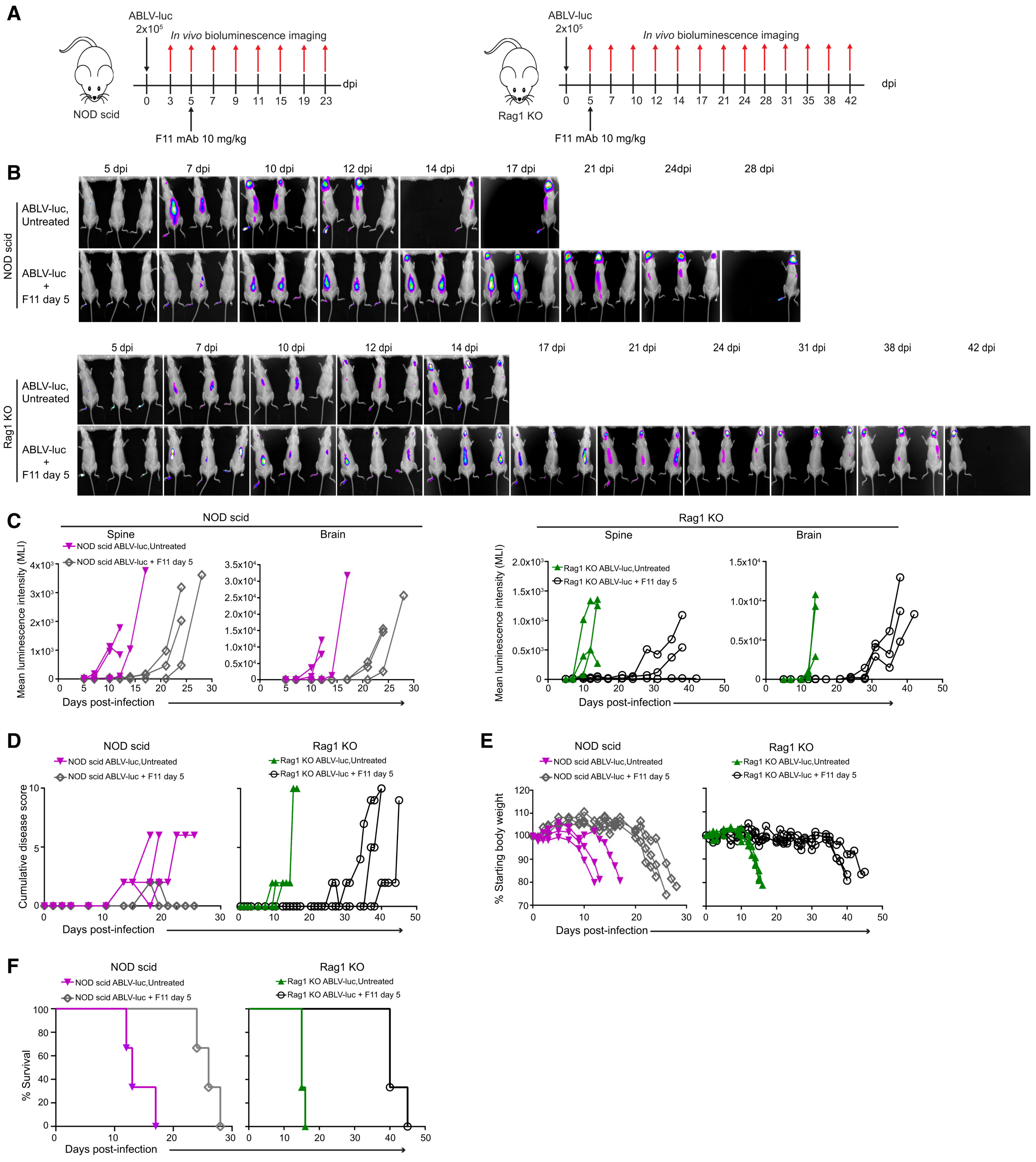

Figure EV2.

© 2023 Commonwealth of Australia and The Authors

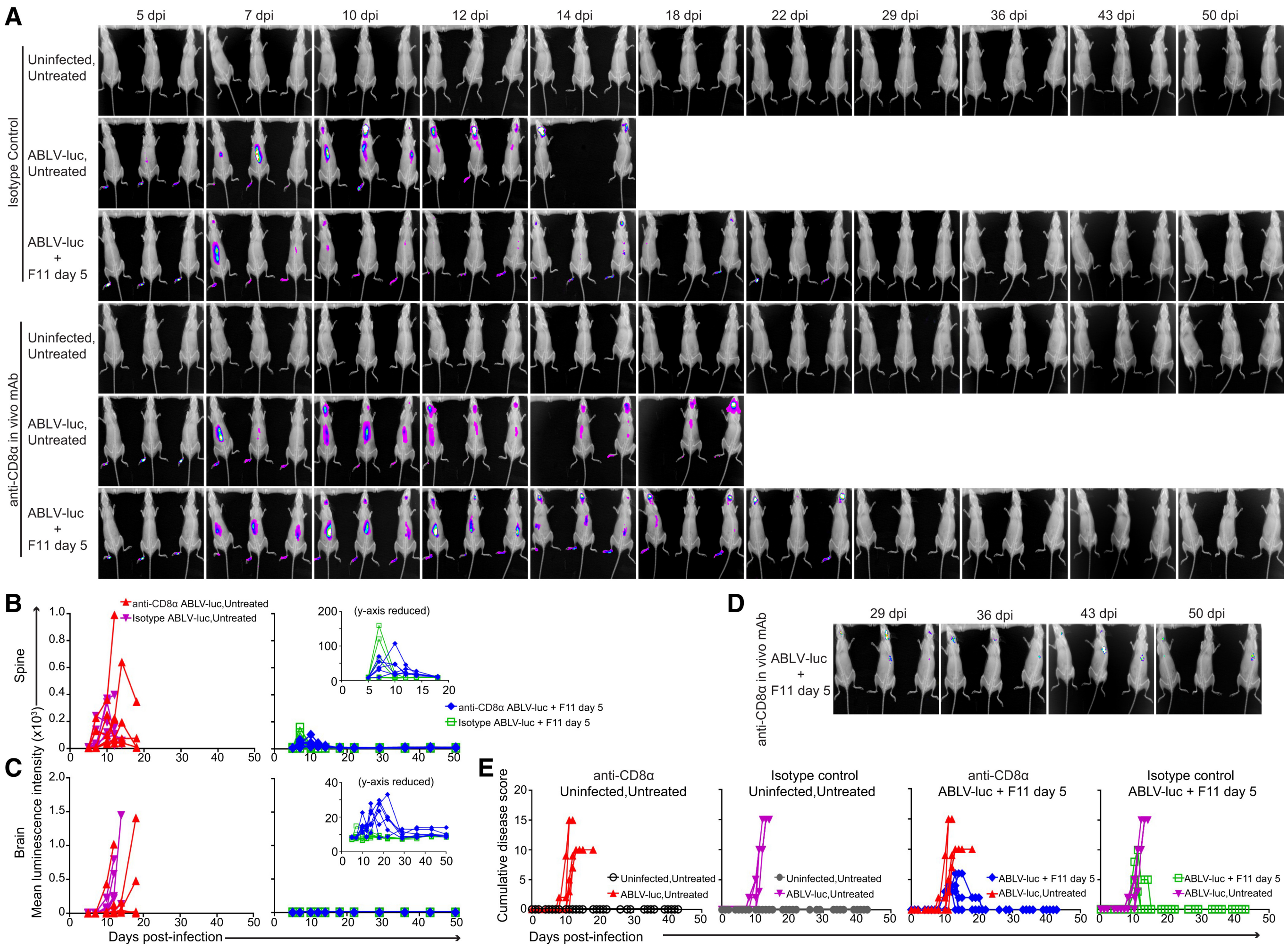

Figure EV3. CD8 T cells do not play an essential role in F11-dependent control of viral infection.

A Bioluminescence imaging of isotype control and CD8 T cell-depleted mice infected with 2 × 10^5 FFU of ABLV-luc and treated with mAb F11 on day 5 (n = 6 mice/group, n = 3 Uninfected, Untreated mice/group).

B, C Viral burden was quantified as mean luminescence intensity (MLI) in the spines (B) and brains (C) of infected mice. Insets are same data with a reduced y-axis scale; note that inset y-axis values are not multiplied by 10^3.

D Bioluminescence imaging of CD8 T cell-depleted mice maintaining low levels of virus within the brain (same data as (A), but with reduced intensity scale to enable visualization of low intensity luminescence).

E Cumulative disease scores were determined by clinical presentation following ABLV-luc challenge.

 © 2023 Commonwealth of Australia and The Authors

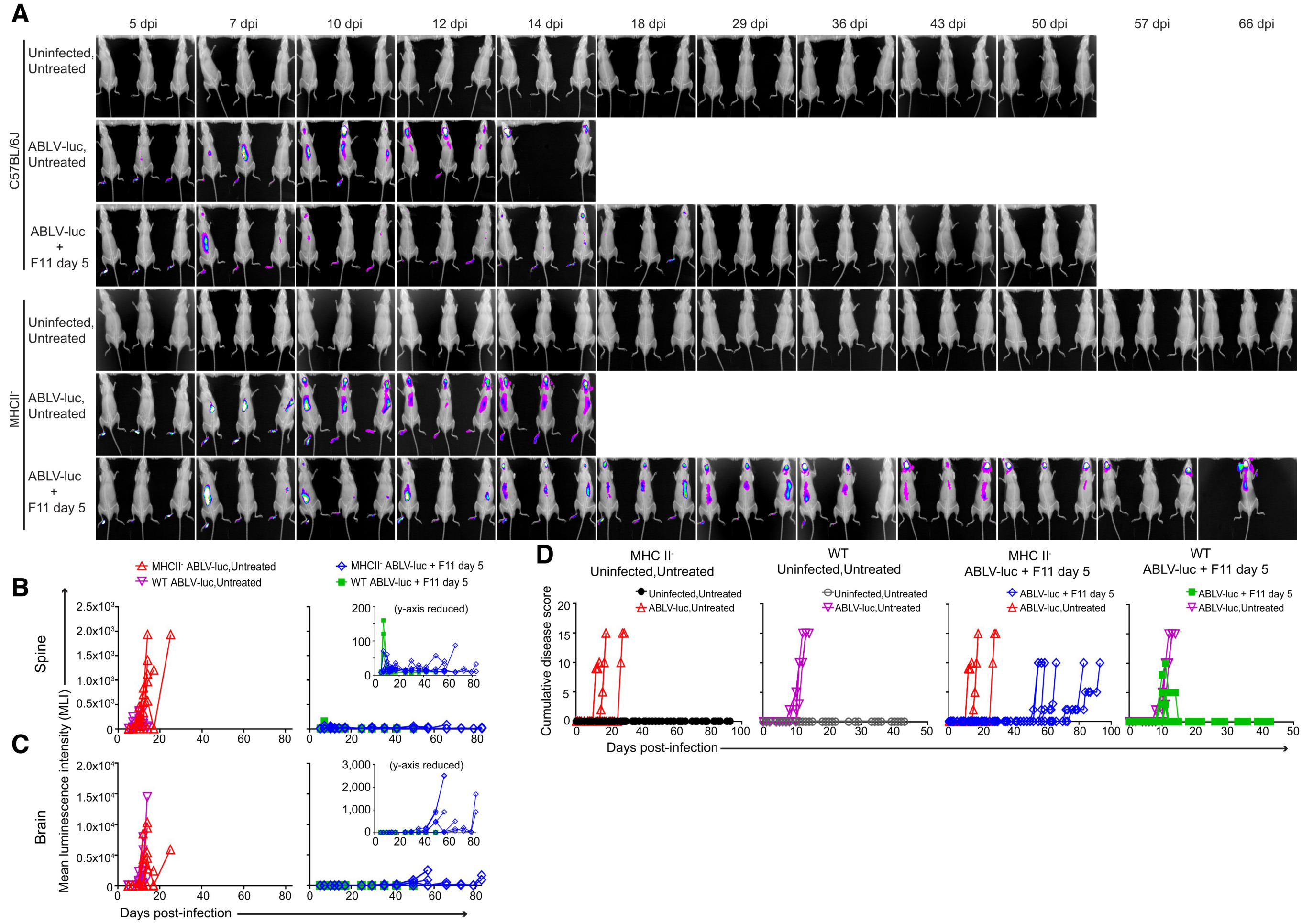

Figure EV4. CD4 T cells are essential for F11-dependent control of viral infection within the CNS.

A Bioluminescence imaging of C57BL/6J and MHCII-mice infected with 2×10^5 FFU of ABLV-luc and treated with mAb F11 on day 5 (n = 6 mice/group, except n = 3 Uninfected, Untreated mice).

B, C Viral burden was quantified as mean luminescence intensity (MLI) in the spines (B) and brains (C) of infected mice. Insets are same data with reduced y-axis scale.

D Cumulative disease scores were determined by clinical presentation following ABLV-luc challenge.

Figure EV5. Flow cytometry and microscopy analyses of leukocyte populations in brains of ABLV-luc-infected mice.

A–C Flow cytometry gating strategy for identification of brain leukocyte populations in mice that were (A) uninfected, (B) ABLV-luc infected, treated with m102.4 on day 5 and euthanized on day 14 post-infection, or (C) ABLV-luc infected, treated with F11 on day 5 and euthanized on day 14 post-infection.

D Animals were treated with m102.4 (n = 5) or F11 (n = 6), and cells were prepared from brains as described in Fig 6A and Materials and Methods. Mean percentages of NK T cells (left) and $\gamma\delta$ T cells (right), P = NS for each population by unpaired t-test and Mann–Whitney test, respectively. Error bars are SEM.

E Animals were treated with m102.4 (n = 5) or F11 (n = 6), and cells were prepared from brains as described in Fig 6A and Materials and Methods. Mean percentages of $MHCII^+$ monocytes, P = NS by unpaired t-test. Error bars are SEM.

F Hindbrains from mice of the indicated treatment groups were fixed and sectioned for histology, followed by staining with DAPI, anti-CD3 and anti-CD4. Examples of typical staining patterns are shown. *indicates cells positive for CD3 and CD4 (CD4-pos), ^indicated cells positive for CD3 only (CD4-neg). Bar, 50 μm.

Source data are available online for this figure.

© 2023 Commonwealth of Australia and The Authors

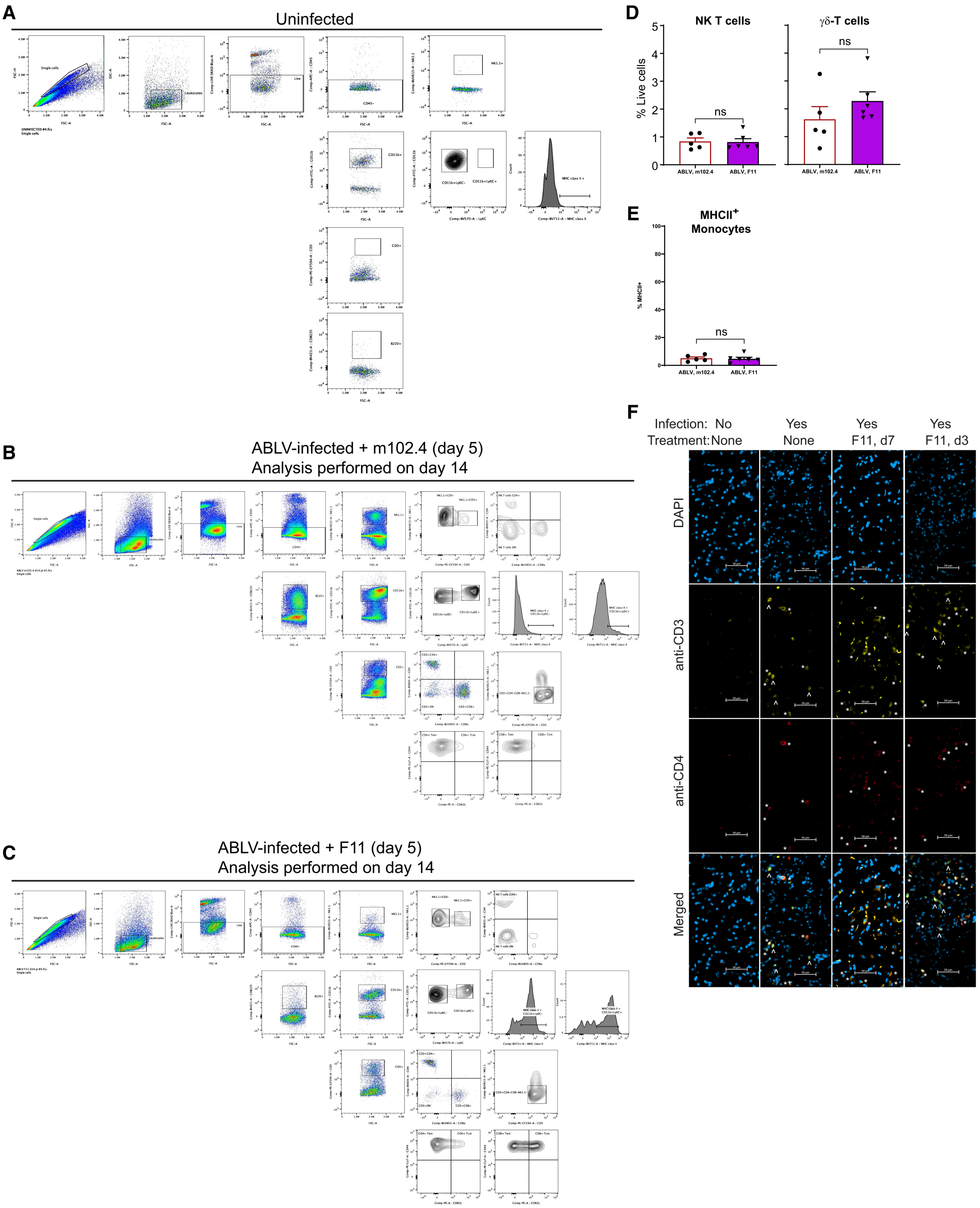

Figure EV5.

 © 2023 Commonwealth of Australia and The Authors
