# Supplementary figures and images for: mAb therapy controls CNS‐resident lyssavirus infection via a CD4 T cell‐dependent mechanism
Source: EMBO Mol Med. 2023 Sep 28;15(10):e16394. doi: 10.15252/emmm.202216394 (PMC10565638; doi:10.15252/emmm.202216394)

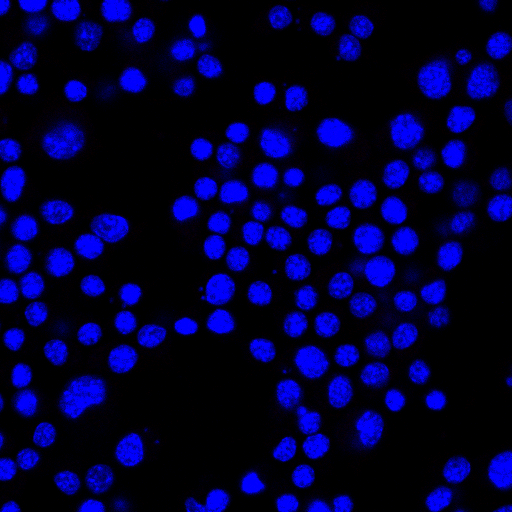

Supplement: Supplementary file 5 — Source Data for Figure 2 [file EMMM-15-e16394-s006.zip › Figure 2A tif files/2A CVS-11 + 6ug_ml F11.tiff]

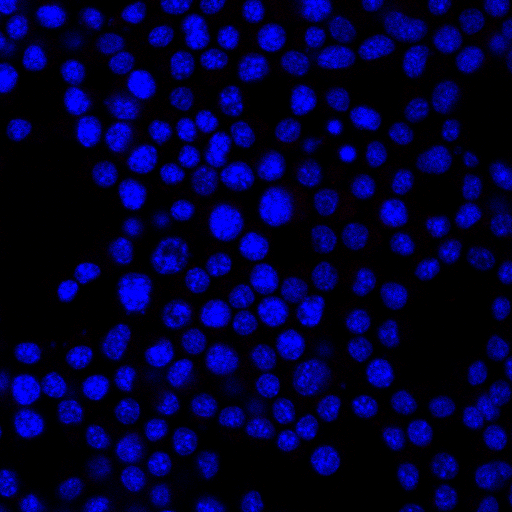

Supplement: Supplementary file 5 — Source Data for Figure 2 [file EMMM-15-e16394-s006.zip › Figure 2A tif files/2A CVS-11 + 60ug_ml F11.tiff]

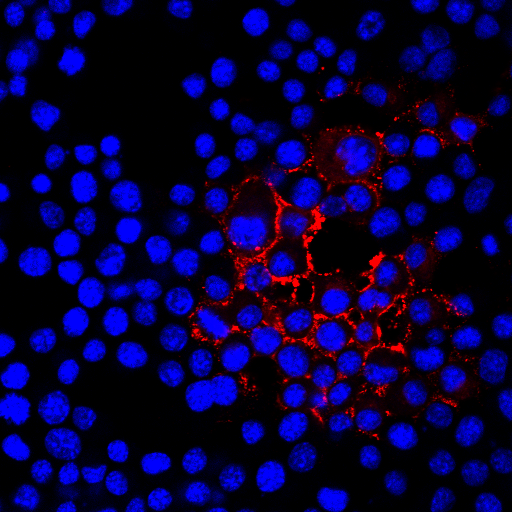

Supplement: Supplementary file 5 — Source Data for Figure 2 [file EMMM-15-e16394-s006.zip › Figure 2A tif files/2A CVS-11 + 0.06ug_ml F11.tiff]

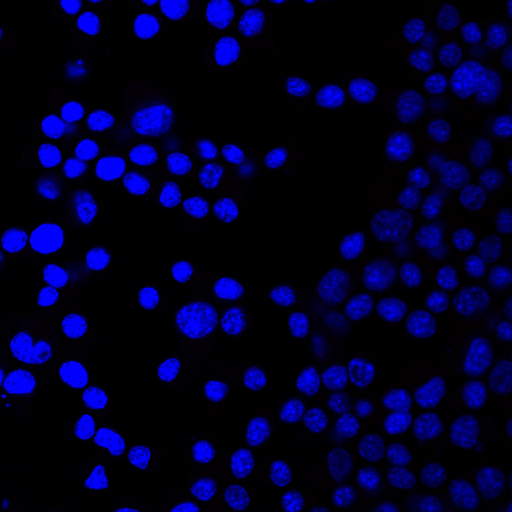

Supplement: Supplementary file 5 — Source Data for Figure 2 [file EMMM-15-e16394-s006.zip › Figure 2A tif files/2A Uninfected + No F11.tiff]

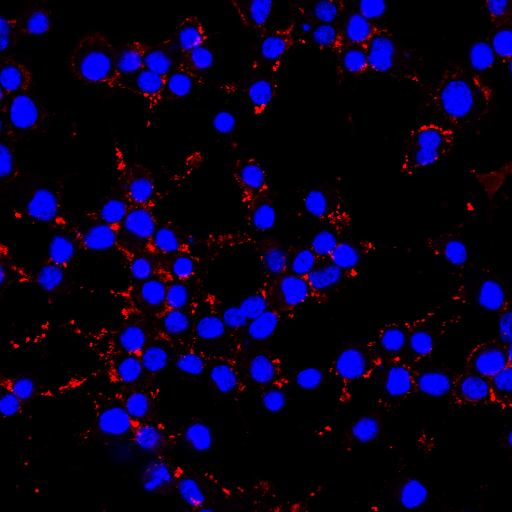

Supplement: Supplementary file 5 — Source Data for Figure 2 [file EMMM-15-e16394-s006.zip › Figure 2A tif files/2A CVS-11 + No F11.tiff]

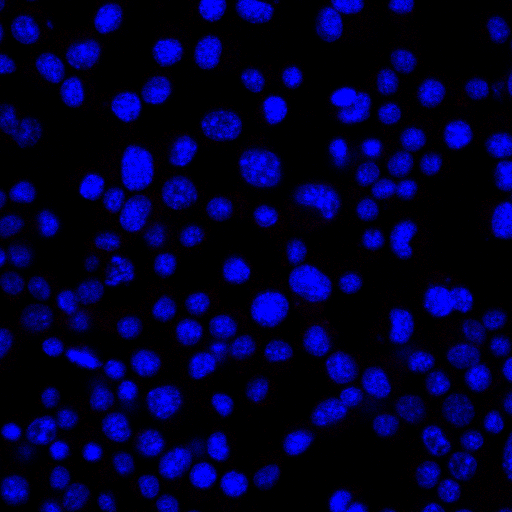

Supplement: Supplementary file 5 — Source Data for Figure 2 [file EMMM-15-e16394-s006.zip › Figure 2A tif files/2A CVS-11 + 600ug_ml F11.tiff]

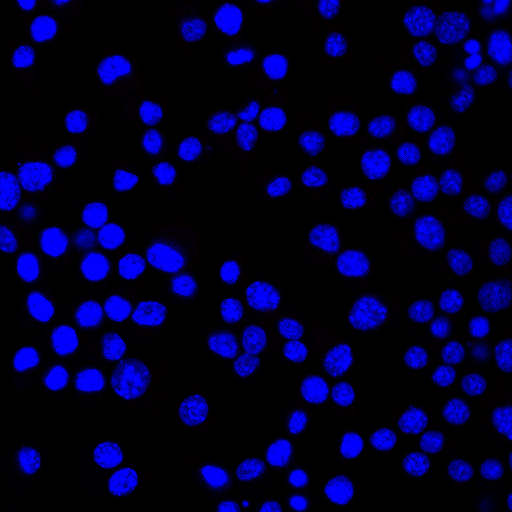

Supplement: Supplementary file 5 — Source Data for Figure 2 [file EMMM-15-e16394-s006.zip › Figure 2A tif files/2A CVS-11 + 0.6ug_ml F11.tiff]

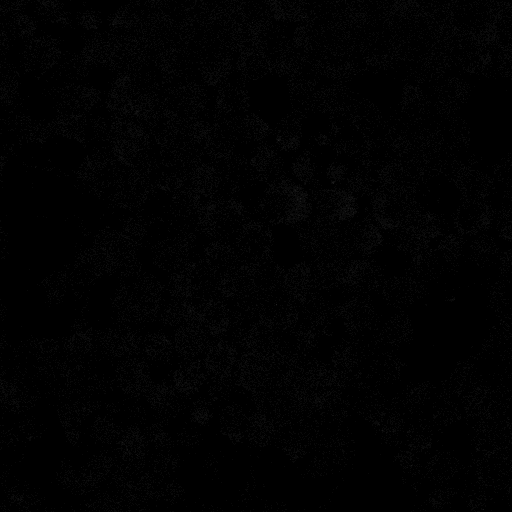

Supplement: Supplementary file 5 — Source Data for Figure 2 [file EMMM-15-e16394-s006.zip › Figure 2A tif files/2A CVS-11 + 60ug_ml F11.tiff_files/2A CVS-11 + 60ug_ml F11_b0t0z0c0x0-512y0-512.tiff]

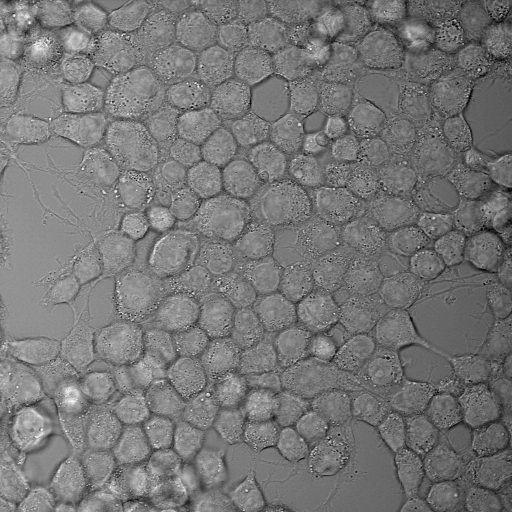

Supplement: Supplementary file 5 — Source Data for Figure 2 [file EMMM-15-e16394-s006.zip › Figure 2A tif files/2A CVS-11 + 60ug_ml F11.tiff_files/2A CVS-11 + 60ug_ml F11_b0t0z0c1x0-512y0-512.tiff]

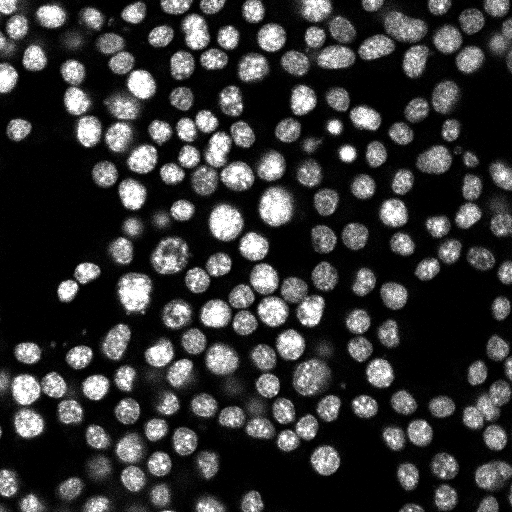

Supplement: Supplementary file 5 — Source Data for Figure 2 [file EMMM-15-e16394-s006.zip › Figure 2A tif files/2A CVS-11 + 60ug_ml F11.tiff_files/2A CVS-11 + 60ug_ml F11_b0t0z0c2x0-512y0-512.tiff]

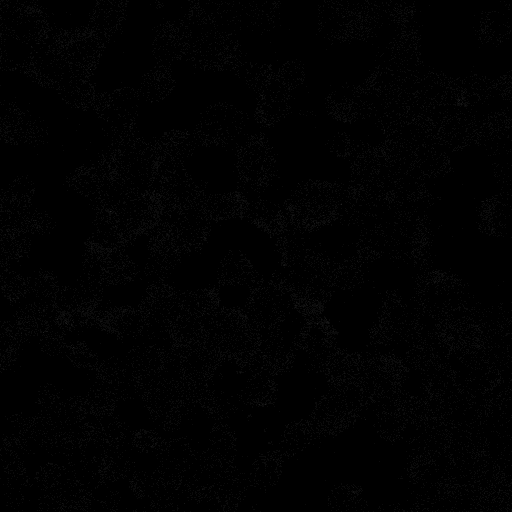

Supplement: Supplementary file 5 — Source Data for Figure 2 [file EMMM-15-e16394-s006.zip › Figure 2A tif files/2A CVS-11 + 600ug_ml F11.tiff_files/2A CVS-11 + 600ug_ml F11_b0t0z0c0x0-512y0-512.tiff]

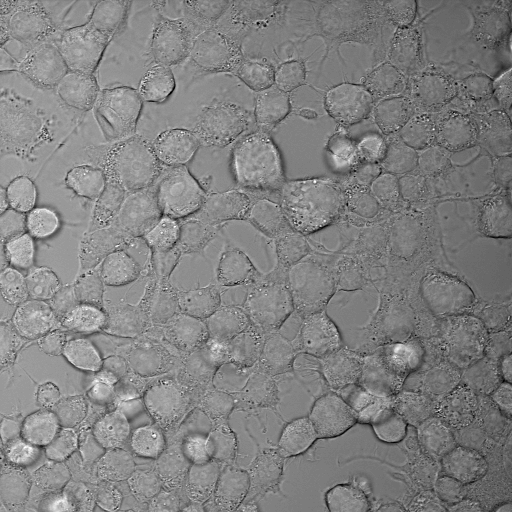

Supplement: Supplementary file 5 — Source Data for Figure 2 [file EMMM-15-e16394-s006.zip › Figure 2A tif files/2A CVS-11 + 600ug_ml F11.tiff_files/2A CVS-11 + 600ug_ml F11_b0t0z0c1x0-512y0-512.tiff]

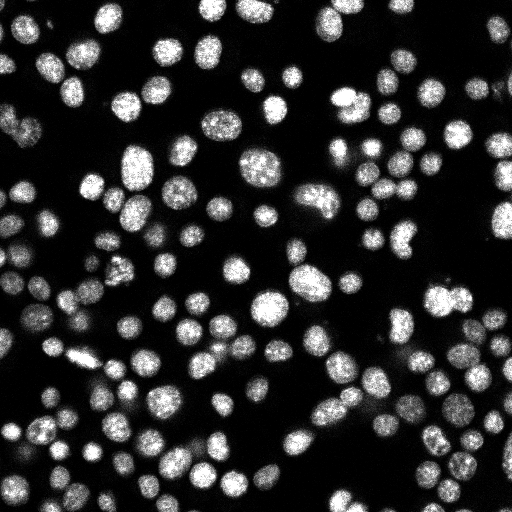

Supplement: Supplementary file 5 — Source Data for Figure 2 [file EMMM-15-e16394-s006.zip › Figure 2A tif files/2A CVS-11 + 600ug_ml F11.tiff_files/2A CVS-11 + 600ug_ml F11_b0t0z0c2x0-512y0-512.tiff]

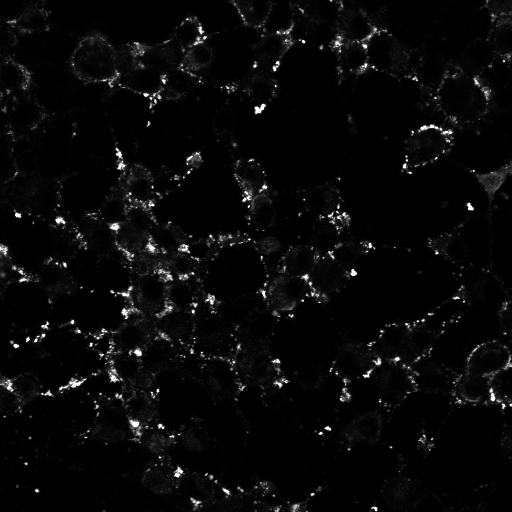

Supplement: Supplementary file 5 — Source Data for Figure 2 [file EMMM-15-e16394-s006.zip › Figure 2A tif files/2A CVS-11 + No F11.tiff_files/2A CVS-11 + No F11_b0t0z0c0x0-512y0-512.tiff]

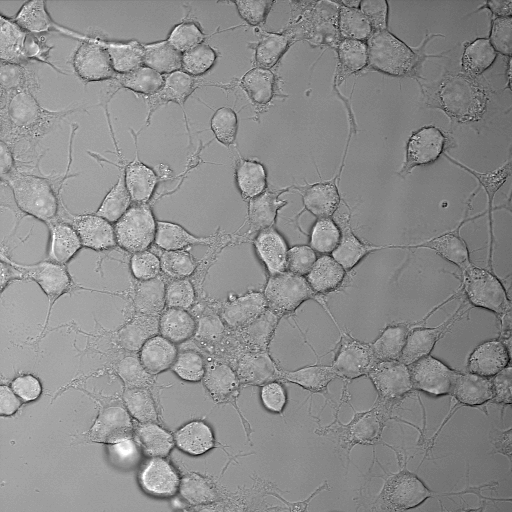

Supplement: Supplementary file 5 — Source Data for Figure 2 [file EMMM-15-e16394-s006.zip › Figure 2A tif files/2A CVS-11 + No F11.tiff_files/2A CVS-11 + No F11_b0t0z0c1x0-512y0-512.tiff]

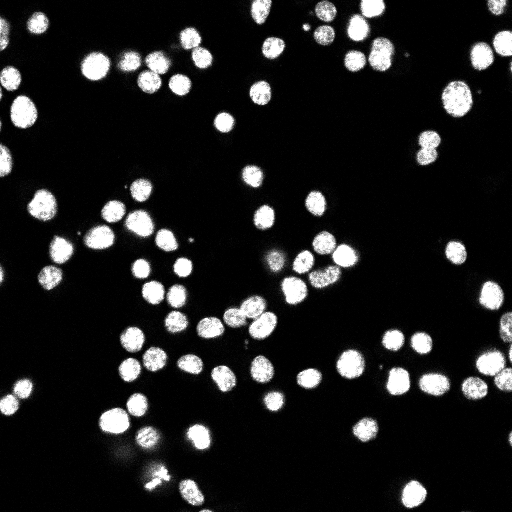

Supplement: Supplementary file 5 — Source Data for Figure 2 [file EMMM-15-e16394-s006.zip › Figure 2A tif files/2A CVS-11 + No F11.tiff_files/2A CVS-11 + No F11_b0t0z0c2x0-512y0-512.tiff]

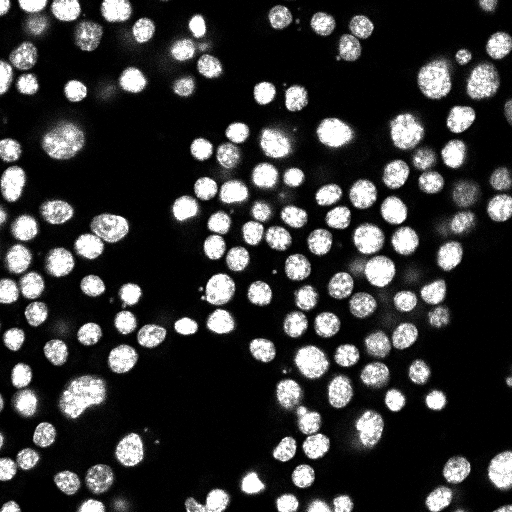

Supplement: Supplementary file 5 — Source Data for Figure 2 [file EMMM-15-e16394-s006.zip › Figure 2A tif files/2A CVS-11 + 6ug_ml F11.tiff_files/2A CVS-11 + 6ug_ml F11_b0t0z0c2x0-512y0-512.tiff]

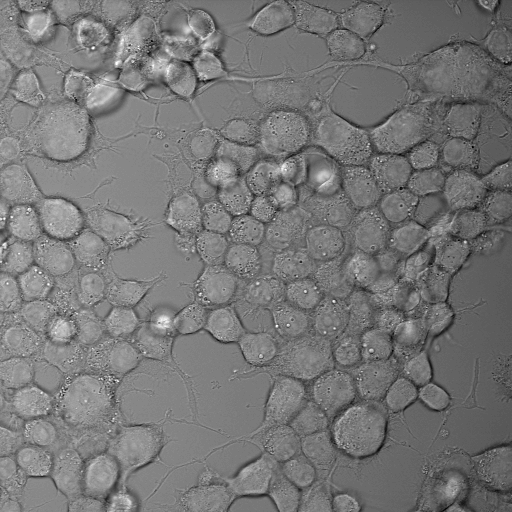

Supplement: Supplementary file 5 — Source Data for Figure 2 [file EMMM-15-e16394-s006.zip › Figure 2A tif files/2A CVS-11 + 6ug_ml F11.tiff_files/2A CVS-11 + 6ug_ml F11_b0t0z0c1x0-512y0-512.tiff]

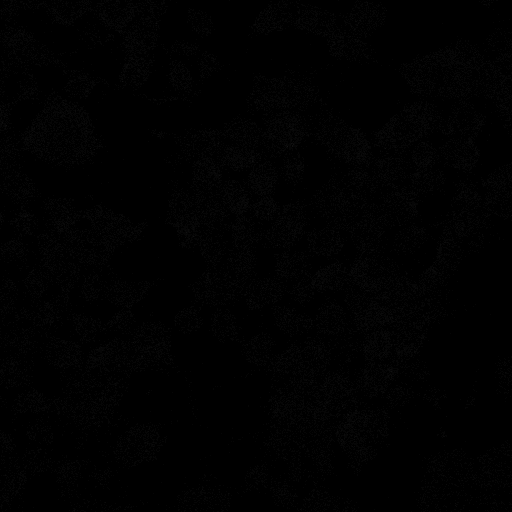

Supplement: Supplementary file 5 — Source Data for Figure 2 [file EMMM-15-e16394-s006.zip › Figure 2A tif files/2A CVS-11 + 6ug_ml F11.tiff_files/2A CVS-11 + 6ug_ml F11_b0t0z0c0x0-512y0-512.tiff]

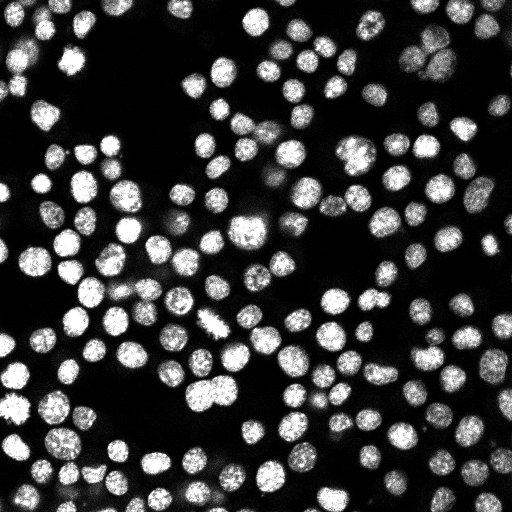

Supplement: Supplementary file 5 — Source Data for Figure 2 [file EMMM-15-e16394-s006.zip › Figure 2A tif files/2A CVS-11 + 0.06ug_ml F11.tiff_files/2A CVS-11 + 0.06ug_ml F11_b0t0z0c2x0-512y0-512.tiff]

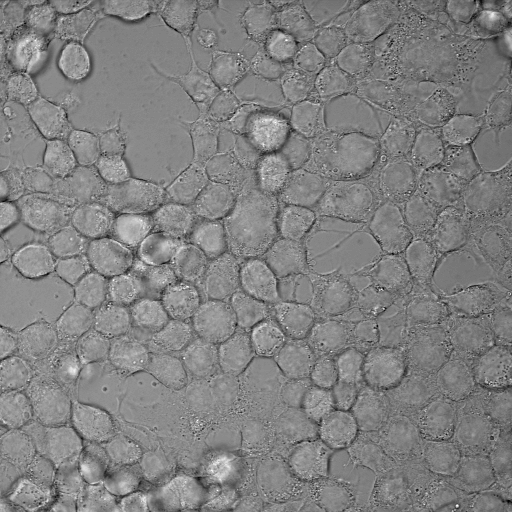

Supplement: Supplementary file 5 — Source Data for Figure 2 [file EMMM-15-e16394-s006.zip › Figure 2A tif files/2A CVS-11 + 0.06ug_ml F11.tiff_files/2A CVS-11 + 0.06ug_ml F11_b0t0z0c1x0-512y0-512.tiff]

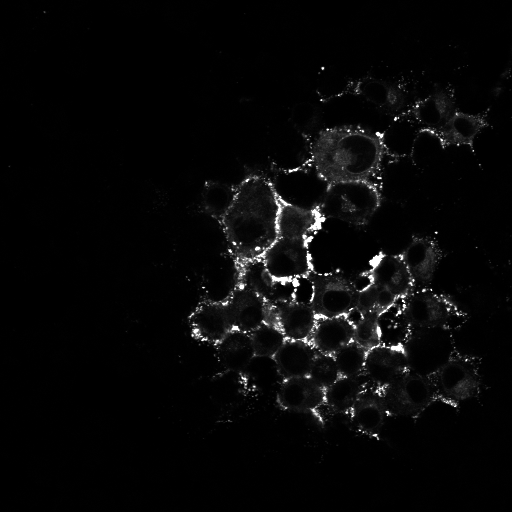

Supplement: Supplementary file 5 — Source Data for Figure 2 [file EMMM-15-e16394-s006.zip › Figure 2A tif files/2A CVS-11 + 0.06ug_ml F11.tiff_files/2A CVS-11 + 0.06ug_ml F11_b0t0z0c0x0-512y0-512.tiff]

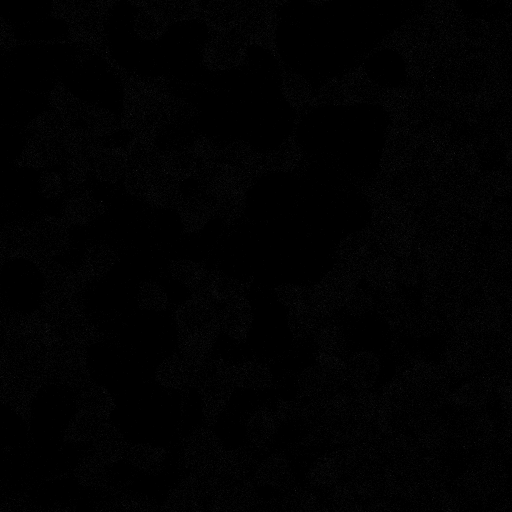

Supplement: Supplementary file 5 — Source Data for Figure 2 [file EMMM-15-e16394-s006.zip › Figure 2A tif files/2A Uninfected + No F11.tiff_files/2A Uninfected + No F11_b0t0z0c0x0-512y0-512.tiff]

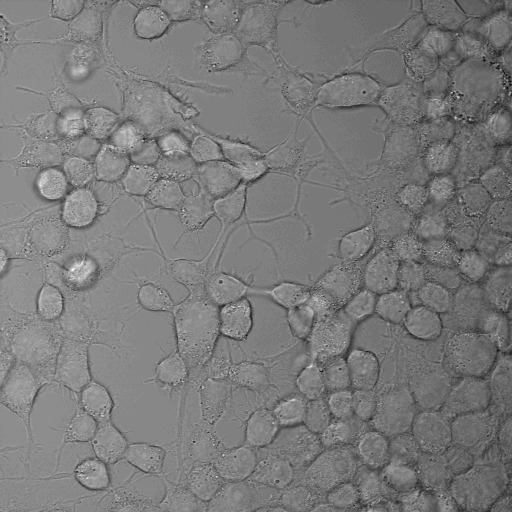

Supplement: Supplementary file 5 — Source Data for Figure 2 [file EMMM-15-e16394-s006.zip › Figure 2A tif files/2A Uninfected + No F11.tiff_files/2A Uninfected + No F11_b0t0z0c1x0-512y0-512.tiff]

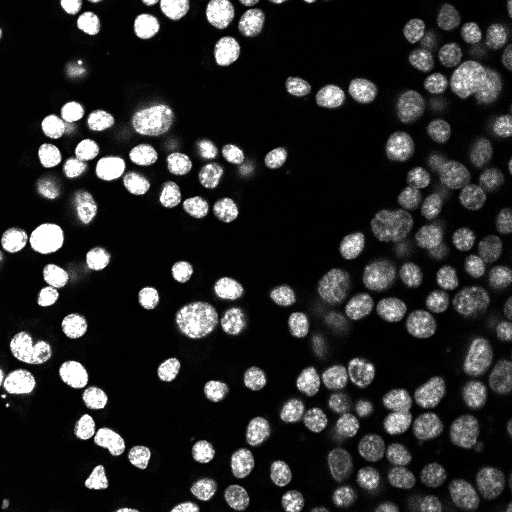

Supplement: Supplementary file 5 — Source Data for Figure 2 [file EMMM-15-e16394-s006.zip › Figure 2A tif files/2A Uninfected + No F11.tiff_files/2A Uninfected + No F11_b0t0z0c2x0-512y0-512.tiff]

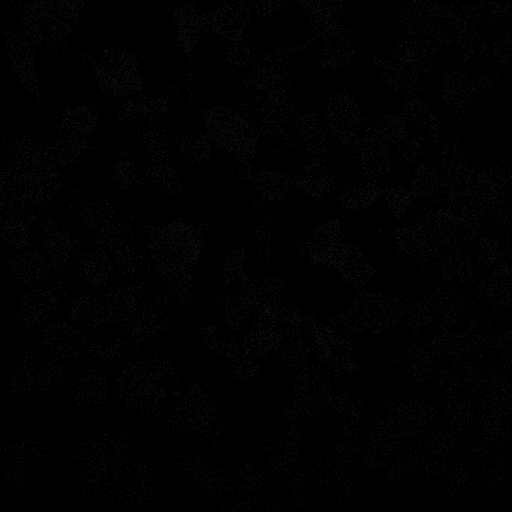

Supplement: Supplementary file 5 — Source Data for Figure 2 [file EMMM-15-e16394-s006.zip › Figure 2A tif files/2A CVS-11 + 0.6ug_ml F11.tiff_files/2A CVS-11 + 0.6ug_ml F11_b0t0z0c0x0-512y0-512.tiff]

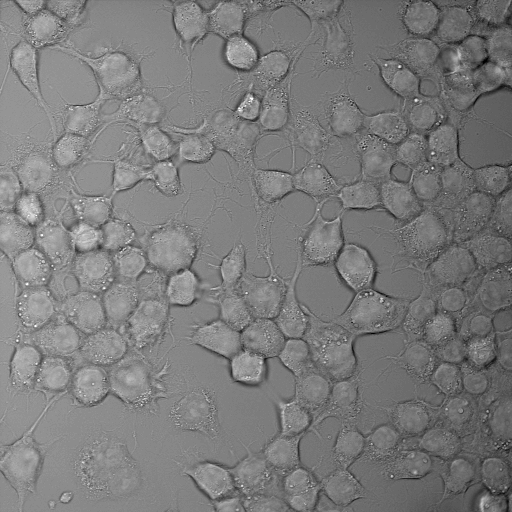

Supplement: Supplementary file 5 — Source Data for Figure 2 [file EMMM-15-e16394-s006.zip › Figure 2A tif files/2A CVS-11 + 0.6ug_ml F11.tiff_files/2A CVS-11 + 0.6ug_ml F11_b0t0z0c1x0-512y0-512.tiff]

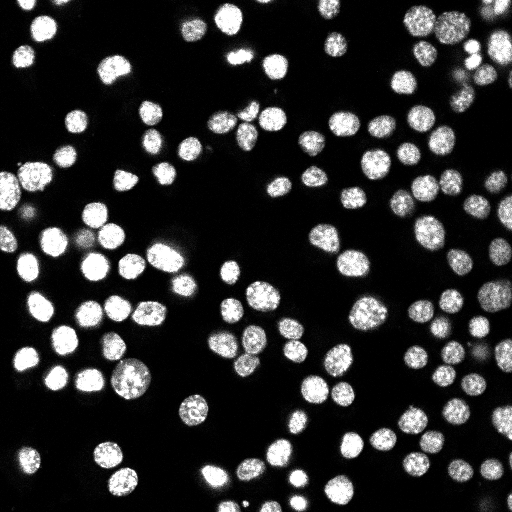

Supplement: Supplementary file 5 — Source Data for Figure 2 [file EMMM-15-e16394-s006.zip › Figure 2A tif files/2A CVS-11 + 0.6ug_ml F11.tiff_files/2A CVS-11 + 0.6ug_ml F11_b0t0z0c2x0-512y0-512.tiff]
