# Supplementary material for: mAb therapy controls CNS‐resident lyssavirus infection via a CD4 T cell‐dependent mechanism
Source: EMBO Mol Med. 2023 Sep 28;15(10):e16394. doi: 10.15252/emmm.202216394 (PMC10565638; doi:10.15252/emmm.202216394)
Supplement: Supplementary file 7 [file EMMM-15-e16394-s005.pdf]

EMBO Molecular Medicine

mAb therapy controls CNS-resident lyssavirus infection via a CD4 T cell-dependent mechanism

Kate Mastraccio, Celeste Huaman, Si'Ana Coggins, Caitlyn Clouse, Madeline Rader, Lianying Yan, Pratyusha Mandal, Imran Hussain, Anwar Ahmed, Trung Ho, Austin Feasley, Bang Vu, Ina Smith, Wanda Markotter, Dawn Weir, Eric Laing, Christopher Broder, and Brian Schaefer
DOI: 10.15252/emmm.202216394

Corresponding author(s): Brian Schaefer (Brian.Schaefer@usuhs.edu) , Christopher Broder (christopher.broder@usuhs.edu)

Review Timeline:

Submission Date:	12th Jun 22
Editorial Decision:	14th Jul 22
Revision Received:	16th Jul 23
Editorial Decision:	11th Aug 23
Revision Received:	17th Aug 23
Accepted:	25th Aug 23

Editor: Zeljko Durdevic

Transaction Report:

(Note: With the exception of the correction of typographical or spelling errors that could be a source of ambiguity, letters and reports are not edited. Depending on transfer agreements, referee reports obtained elsewhere may or may not be included in this compilation. Referee reports are anonymous unless the Referee chooses to sign their reports.)

14th Jul 2022

Dear Dr. Schaefer,

Thank you for the submission of your manuscript to EMBO Molecular Medicine. We have now received feedback from the three reviewers who agreed to evaluate your manuscript. As you will see from the reports below, the referees acknowledge the interest of the study but also raise important and partially overlapping concerns that should be addressed in a major revision. During our cross-commenting session it became clear that the rear footpad as a challenge method is not appropriate and limits the translational potential and clinical relevance of the study. Therefore, focus of the revision should be in performing experiments using intracranial inoculation as a challenge method.

Further consideration of a revision that addresses reviewers' concerns in full will entail a second round of review. EMBO Molecular Medicine encourages a single round of revision only and therefore, acceptance or rejection of the manuscript will depend on the completeness of your responses included in the next, final version of the manuscript. For this reason, and to save you from any frustrations in the end, I would strongly advise against returning an incomplete revision.

We would welcome the submission of a revised version within six months for further consideration. Please let us know if you require longer to complete the revision.

I look forward to receiving your revised manuscript.

Yours sincerely,

Zeljko Durdevic

Zeljko Durdevic
Editor
EMBO Molecular Medicine

*****

When submitting your revised manuscript, please carefully review the instructions that follow below. We perform an initial quality control of all revised manuscripts before re-review; failure to include requested items will delay the evaluation of your revision.

We require:

1) A .docx formatted version of the manuscript text (including legends for main figures, EV figures and tables). Please make sure that the changes are highlighted to be clearly visible.

2) Individual production quality figure files as .eps, .tif, .jpg (one file per figure). For guidance, download the 'Figure Guide PDF': (https://www.embopress.org/page/journal/17574684/authorguide#figureformat).

3) A .docx formatted letter INCLUDING the reviewers' reports and your detailed point-by-point responses to their comments. As part of the EMBO Press transparent editorial process, the point-by-point response is part of the Review Process File (RPF), which will be published alongside your paper.

4) A complete author checklist, which you can download from our author guidelines (https://www.embopress.org/page/journal/17574684/authorguide#submissionofrevisions). Please insert information in the checklist that is also reflected in the manuscript. The completed author checklist will also be part of the RPF.

5) Please note that all corresponding authors are required to supply an ORCID ID for their name upon submission of a revised manuscript.

6) It is mandatory to include a 'Data Availability' section after the Materials and Methods. Before submitting your revision, primary datasets produced in this study need to be deposited in an appropriate public database, and the accession numbers and database listed under 'Data Availability'. Please remember to provide a reviewer password if the datasets are not yet public (see https://www.embopress.org/page/journal/17574684/authorguide#dataavailability).

In case you have no data that requires deposition in a public database, please state so in this section. Note that the Data Availability Section is restricted to new primary data that are part of this study.

7) For data quantification: please specify the name of the statistical test used to generate error bars and P values, the number (n) of independent experiments (specify technical or biological replicates) underlying each data point and the test used to calculate p-values in each figure legend. The figure legends should contain a basic description of n, P and the test applied. Graphs must include a description of the bars and the error bars (s.d., s.e.m.). See also 'Figure Legend' guidelines: https://www.embopress.org/page/journal/17574684/authorguide#figureformat

8) We would also encourage you to include the source data for figure panels that show essential data. Numerical data should be provided as individual .xls or .csv files (including a tab describing the data). For blots or microscopy, uncropped images should be submitted (using a zip archive if multiple images need to be supplied for one panel). Additional information on source data and instruction on how to label the files are available at

.

9) Our journal encourages inclusion of *data citations in the reference list* to directly cite datasets that were re-used and obtained from public databases. Data citations in the article text are distinct from normal bibliographical citations and should directly link to the database records from which the data can be accessed. In the main text, data citations are formatted as follows: "Data ref: Smith et al, 2001" or "Data ref: NCBI Sequence Read Archive PRJNA342805, 2017". In the Reference list, data citations must be labeled with "[DATASET]". A data reference must provide the database name, accession number/identifiers and a resolvable link to the landing page from which the data can be accessed at the end of the reference. Further instructions are available at .

10) We replaced Supplementary Information with Expanded View (EV) Figures and Tables that are collapsible/expandable online. A maximum of 5 EV Figures can be typeset. EV Figures should be cited as 'Figure EV1, Figure EV2" etc... in the text and their respective legends should be included in the main text after the legends of regular figures.

- For the figures that you do NOT wish to display as Expanded View figures, they should be bundled together with their legends in a single PDF file called *Appendix*, which should start with a short Table of Content. Appendix figures should be referred to in the main text as: "Appendix Figure S1, Appendix Figure S2" etc.

- Additional Tables/Datasets should be labeled and referred to as Table EV1, Dataset EV1, etc. Legends have to be provided in a separate tab in case of .xls files. Alternatively, the legend can be supplied as a separate text file (README) and zipped together with the Table/Dataset file.

See detailed instructions here:

.

11) The paper explained: EMBO Molecular Medicine articles are accompanied by a summary of the articles to emphasize the major findings in the paper and their medical implications for the non-specialist reader. Please provide a draft summary of your article highlighting

- the medical issue you are addressing,

- the results obtained and

- their clinical impact.

This may be edited to ensure that readers understand the significance and context of the research. Please refer to any of our published articles for an example.

12) For more information: There is space at the end of each article to list relevant web links for further consultation by our readers. Could you identify some relevant ones and provide such information as well? Some examples are patient associations, relevant databases, OMIM/proteins/genes links, author's websites, etc...

13) Author contributions: the contribution of every author must be detailed in a separate section (before the acknowledgments).

14) A Conflict of Interest statement should be provided in the main text.

15) Every published paper now includes a 'Synopsis' to further enhance discoverability. Synopses are displayed on the journal webpage and are freely accessible to all readers. They include a short stand first (maximum of 300 characters, including space) as well as 2-5 one-sentences bullet points that summarizes the paper. Please write the bullet points to summarize the key NEW findings. They should be designed to be complementary to the abstract - i.e. not repeat the same text. We encourage inclusion of key acronyms and quantitative information (maximum of 30 words / bullet point). Please use the passive voice. Please attach these in a separate file or send them by email, we will incorporate them accordingly.

Please also suggest a striking image or visual abstract to illustrate your article as a PNG file 550 px wide x 300-600 px high.

EMBO Molecular Medicine has a "scooping protection" policy, whereby similar findings that are published by others during review or revision are not a criterion for rejection. Should you decide to submit a revised version, I do ask that you get in touch after three months if you have not completed it, to update us on the status.

***** Reviewer's comments *****

Referee #1 (Comments on Novelty/Model System for Author):

The mouse model is good choice for lyssavirus infection and antibody therapy.

Referee #1 (Remarks for Author):

In this present study, the authors evaluated the therapeutic efficacy of the anti-lyssavirus human monoclonal antibody (mAb) F11 on established Australian bat lyssavirus (ABLV) infections in a mouse model. Specially, luminescence-based longitudinal tracing of virus infection was widely used to evaluate the therapeutic efficacy of F11. It was found that F11-dependent neutralization is not sufficient to protect animals from mortality. Consistent with the previous studies working on the pathogenesis of rabies virus, a CD4 T cell, but not CD8 T cell-dependent adaptive immune response is necessary for successful control of lyssavirus infection although the detailed mechanism is not totally understood yet. Besides, the authors reveal that the FcRγ binding function of F11 contributes to therapeutic efficacy, although the detailed mechanism is not investigated. Together, this study demonstrates that mAb therapy is a promising strategy against an established CNS infection by a lethal neurotropic virus. However, the mechanisms behind the therapy need more investigation in the future. There are some concerns for this study need to be addressed.
Major ones:
1. A recent study has described the therapeutic efficacy of one pair of human monoclonal antibodies against rabies virus by intracerebroventricular infusion to directly deliver antibodies into the CNS (EMM, 2020 10.15252/emmm.202012628). This important study should to be cited and discussed.
2. According to the previous studies, the researchers generally inoculated the antibodies into the brain. In this study, the authors injected only one shot of F11 into the periphery. However, it is difficult for F11 to pass through the BBB (no data in the manuscript showed about this concern). After ABLV entered into the brain in the later days post infection, what the mechanism for F11 to clear ABLV? Is there any F11 presenting in the mouse brain?
3. These data shown in Fig. 4 indicate that CD8 T cells are required for F11-mediated survival following ABLV infection, which is consistent with the previous findings (Hooper DC et al. JVI, 1998). These negative data should be put into the supplemental data.
4. In Fig. 6, why not use FACS to quantified the infiltrated T cells in the mouse brains? Only IHC staining and quantification is not convincing. Besides T cells, the B cells in the brain also should be investigated by FACS since the production of antibody by invading B cells is required for the clearance of rabies virus from the CNS (PLoS neglected tropical diseases, 2009, 10.1371/journal.pntd.0000535).
5. CD4 T cells have been found to play a critical role against lyssavirus infection (Aurore Lebrun et al. Journal of Immunology,2015; Emily K Bongiorno, et al. Journal of Immunology,2017). According to these studies, IFN-r produced by CD4 T cells is critical for BBB opening and clearance of rabies virus invaded into the CNS. Thus, in this study the exact mechanism for CD4 T cells to clear ABLV in the brain need to be extended or at least discussed carefully.
6. In Fig. 7, what is the mechanism for FcRγ to contribute to the therapeutic efficacy of F11? At least it should be discussed.
7. It is weird to present the qPCR and IHC data by using tables (table 1-3). Using columns may be the better choice.

Minor ones:
1. The line numbers and page numbers are missing. It is painful to point out the mistakes in the manuscript.
2. The IACUC number in the Materials and Methods is missing.
3. In the results part, the injection routes for antibody injection or virus challenge need to be described.

Referee #2 (Comments on Novelty/Model System for Author):

The topic adressed is interesting and could have a medical impact. However, the infection model used, ABLV, is useful but the study of host immune responses in a rabies infection model, would have had a higher medical impact.

The assesment of host-immune responses deserve further investigation.

The authors state that mAb therapy has an "immune provocking" effect. However, the data presented show a role for host immune responses in mAb-mediated protection but do not allow concluding an "immune provocking" effect of mAb therapy. Such "immune provocking" or "vaccinal effects" with enhanced cellular and humoral host immune responses have been more precisely described/demonstrated in other experimental settings.

Referee #2 (Remarks for Author):

Mastraccio et al, reports an antibody-based therapeutic approach to lyssavirus infection. Using mouse models of lyssavirus infection, the authors show that administration of a neutralizing mAb after established infection protects infected mice. These results could have a potential medical impact, as lyssavirus is almost 100% fatal in symptomatically infected patients. The novelty of this work is that neutralization is not sufficient to protect animals from mortality, but that host immune responses are required to successfully control infection. However, the immune mechanisms involved may require further characterization.

Major points

1. The authors conclude that "CD8 T cells may contribute to control of viral infection, but this population is not essential for F11-dependent survival after lyssavirus challenge." Challenge experiments were performed at 82 dpi. However, CD8 T cell depletion was performed at early post-infection time points. What about CD8 T-cell frequency before viral challenge 82 dpi? If CD8 T cell frequency was restored, the authors cannot rule out a potential role of these cells in the observed protective effect.

2. What about the humoral immune response upon viral challenge?

3. While CD4 T-cell deficient mice show no long-term protection upon mAb treatment, the role of CD4 T cells in mAb-mediated protection is not well identified. Indeed, the authors show in figure 6 similar number of CD4 T cells in protected and unprotected animals. Further characterization of CD4 T cells (i.e. subsets, phenotypic and/or functional activation) might shed light on their role in protection observed after mAb treatment).

4. The "CD4-neg" population is poorly defined. CD3+ cells may also include other cell subsets (i.e. NKT cells). As the authors suggest a potential role or this population in virus-induced immunopathology, a better characterization of this population might be required using experimental approaches other than IHC. In keeping with this, further characterization of cytokines and innate immune mediators'profile in the brains of mice from different experimental groups would be informative.

5. FcRγ binding function of mAb contribution to therapeutic efficacy is mild and has also been described in other pathological contexts of rabies infection:

de Melo GD, et al, A combination of two human monoclonal antibodies cures symptomatic rabies. EMBO Mol Med. 2020 Nov 6;12(11):e12628. doi: 10.15252/emmm.202012628.

6. A few passages in the discussion are speculative:

- "Moreover, we demonstrated that surviving, F11-treated animals exhibit sterilizing anti-lyssavirus peripheral immunity, as well as a long-lasting increase in expression of signature cellular immune response genes in the brain ». No sterilizing immunity is observed as residual viral load is detected by PCR approaches. There is not long-lasting increase in expression of signature cellular immune response genes in the brain, it is similar to that observed in early stages as well as similar to, or even lower than that observed in untreated mice.

-"Therefore, the administration of F11 on day 5 or day 7 post-infection may result in delivery of viral antigen-loaded APCs in increased numbers or with higher stimulatory capacity to differentiating CD4 T cells, yielding higher numbers or altered functionality of anti- lyssavirus effector CD4 T cells ". See comment n{degree sign}3.

-" By contrast to enhanced virus control following day 5 and day 7 therapy, administration of F11 on day 3 may deliver viral antigen to DCs too early to have a positive effect on priming, or it may deplete the infectious virus and viral antigen too efficiently to result in effective priming of the anti-lyssavirus CD4 T cell response ».

-" our data show an immune provoking component of mAb therapy which is ultimately essential for therapeutic efficacy". The data presented show a role for host immune responses in mAb-mediated protection but do not allow concluding an "immune provocking" effect of mAb therapy.

Minor comments

1.The route of administration of the mAb is not described.

2. In figure 3, panel F, the time point of coronal sections is not indicated. Same for Fig S1C.

3. The reason why albino mice are used in some experiments and not in others is not explained.

4. Correct in the introduction "To date, there has been no small molecule- or biologic-based therapeutic approach demonstrated to reliably treat CNS-resident lyssavirus infection, preventing mortality" (see reference De Melo, 2020 above mentioned).

5. Define PEP, in the introduction

Referee #3 (Comments on Novelty/Model System for Author):

please see my comments to the authors below

Referee #3 (Remarks for Author):

Major point of critiques:
1. "Infections with rabies virus (RABV) and related lyssaviruses are uniformly fatal" - are you talking about people here? Because infections with rabies virus (RABV) and related lyssaviruses in wild animals are NOT uniformly fatal.
2. "...are uniformly fatal, once virus accesses the central nervous system (CNS)" - totally incorrect! Only after clinical symptoms developed. Fix it over the entire text.
3. "...and reverses disease signs" - be clear which signs of rabies have been reversed.
4. "...cause encephalitic clinical symptoms (Olival & Daszak, 2005)." - or neurological clinical symptoms?
5. It is not clear why the authors emphasized bat-borne rabies in the second paragraph of the introduction. The main source of human rabies in the world is rabid dogs (most importantly!) and other carnivores (much less!), not bats.
6. Please indicate all abbreviations in the text the first time they are used. Fix it over the entire text.
7. "...and exposure site injection of polyspecific human or equine rabies immunoglobulin (HRIG or ERIG, respectively)" - incorrect! HRIG or ERIG are not always indicated!
8. "mAbs may have therapeutic value, administration of mAbs was performed prior to or within 2 hours of RABV infection" - I think you are talking about mouse model here. Please indicate it in the text. It is not a human or NHP data.
9. The Introduction is too long and can be cut in half. The last paragraph of the Introduction should be moved to Discussion.
10. Why outbred mice, commonly used in the rabies NIH test, not used here? Why special inbred strains were used?
11. "For rabies challenge studies, a rodent-adapted RABV strain (CVS-11) was used (ATCC Cat#VR-959)." - how it was grown and prepared for the study?
12. It is not clear how many TCID50 of ABLV-luc or CVS-11 were used in In vitro neutralization assays? Normally, it should be between 50-100 TCID50 per the tested antibody dilution. The potency of your mAb F11 should be assessed in the conventional RFFIT test.
13. Very unusual challenge method is used - in rear footpad! I don't think that it is equivalent to commonly used challenge methods for laboratory rodents - IC and IN. Second, there is no data provided on validity of this challenge method and challenge model. I'm not sure if the same results with your F11 mAb will be shown in outbred mice infected with rabies using the conventional IC and IN infection methods. In my opinion, an incorrect, mild model was selected.
14. What was the sensitivity (in rabies virus copies in the CNS tissues) of your In vivo bioluminescence imaging? I'm not sure that this bioluminescence imaging is very sensitive for detection of rabies virus replication in the CNS!
15. Why brain sections were stained with mouse anti-rabies G (clone 1C5, Novus Biologicals Cat#NBP2-11630) overnight at 4{degree sign}C? It is very unusual protocol!
16. The "Results" section is too wordy, and many paragraphs looks like the "Discussion" section.
17. The Discussion is too long and can be cut in half.
18. The originality of this study in respect that T cell-dependent immune response is required for successful control of rabies infection in the CNS is questionable, because it was previously demonstrated in several publications (as examples: J Immunol. 2015 Nov 1; 195(9): 4358-4368; J Virol . 2018 Mar 28;92(8):e02029-17; PLoS Negl Trop Dis. 2015 Aug 20;9(8):e0004023; J Virol. 2001 Apr;75(7):3427-34.)

EMM-2022-16394-V2 Responses to Reviewers

We very much appreciate the detailed and thoughtful critiques from the reviewers regarding our manuscript. We have endeavored to address reviewer critiques as completely and thoroughly as possible. Our specific responses are **in bold type**, after each reviewer statement (indicated by *italics*):

******* Reviewer's comments and Author's responses *******

Referee #1 *(Comments on Novelty/Model System for Author):*

The mouse model is good choice for lyssavirus infection and antibody therapy.

Referee #1 *(Remarks for Author):*

In this present study, the authors evaluated the therapeutic efficacy of the anti-lyssavirus human monoclonal antibody (mAb) F11 on established Australian bat lyssavirus (ABLV) infections in a mouse model. Specially, luminescence-based longitudinal tracing of virus infection was widely used to evaluate the therapeutic efficacy of F11. It was found that F11-dependent neutralization is not sufficient to protect animals from mortality. Consistent with the previous studies working on the pathogenesis of rabies virus, a CD4 T cell, but not CD8 T cell-dependent adaptive immune response is necessary for successful control of lyssavirus infection although the detailed mechanism is not totally understood yet. Besides, the authors reveal that the FcRγ binding function of F11 contributes to therapeutic efficacy, although the detailed mechanism is not investigated. Together, this study demonstrates that mAb therapy is a promising strategy against an established CNS infection by a lethal neurotropic virus. However, the mechanisms behind the therapy need more investigation in the future. There are some concerns for this study need to be addressed.

Major ones:
1. *A recent study has described the therapeutic efficacy of one pair of human monoclonal antibodies against rabies virus by intracerebroventricular infusion to directly deliver antibodies into the CNS (EMM, 2020 10.15252/emmm.202012628). This important study should to be cited and discussed.*

We apologize for this omission, and now prominently mention this very important study both in the introduction and discussion.

2. *According to the previous studies, the researchers generally inoculated the antibodies into the brain. In this study, the authors injected only one shot of F11 into the periphery. However, it is difficult for F11 to pass through the BBB (no data in the manuscript showed about this concern). After ABLV entered into the brain in the later days post infection, what the mechanism for F11 to clear ABLV? Is there any F11 presenting in the mouse brain?*

To address the issue of F11 penetrance into the CNS, we have stained brain sections of F11-treated animals with anti-human IgG. This is now included as Appendix Fig S4. While we detect sporadic cell-associated F11 (which, we believe most likely reflects leukocytes that transcytosed from the blood), we failed to detect widespread F11,

suggesting that there is not a meaningful breach of the BBB that occurs in F11-treated animals (the lack of peripherally administered mAb reaching the brains of lyssavirus-infected mice is consistent with published data from other groups, e.g., Hooper DC, et al (2009). PLoS Negl Trop Dis 3: e535). Also, because F11 delivered at day 3 is poorly effective while F11 delivered 2 days later is 100% effective, the data suggest that the effect of F11 is not simple (i.e., not simply crossing the BBB and neutralizing virus in the CNS). Moreover, the results with many strains of immunodeficient animals in this manuscript, showing NO protection from mortality by F11, strongly suggests that, even if F11 can access the CNS, the amounts that enter the brain are insufficient to mediate protection from mortality.

The collective data in our manuscript support a model in which CD4 T cells cross the BBB and are responsible for directing a CNS immune response that controls lyssavirus infection by an uncertain mechanism. Notably, we have included new flow cytometry data showing a major and highly significant shift in diverse leukocyte populations in the brain, resulting from F11 therapy (Fig 6A-D, Fig EV5A-E). Also, we made a more highly impaired Fc mutant of F11, called LALA-PG, and we show that this mutant is significantly less active in protection of animals from mortality (Fig 7). Thus, the data are consistent with F11 mediating dramatic changes to the cellular immune response which require *both* CD4 T cells and F11 Fc function.

3. *These data shown in Fig. 4 indicate that CD8 T cells are required for F11-mediated survival following ABLV infection, which is consistent with the previous findings (Hooper DC et al. JVI, 1998). These negative data should be put into the supplemental data.*

The cited data are in the context of host-mediated clearance of an attenuated strain of rabies, in which no therapy was administered. By contrast, the purpose of our study is to mechanistically define the basis of a novel rabies therapy. Thus, we believe the CD8 T cell data are of high enough relevance to be maintained as a main figure. However, we are happy to follow the guidance of the editors on this subject.

4. *In Fig. 6, why not use FACS to quantified the infiltrated T cells in the mouse brains? Only IHC staining and quantification is not convincing. Besides T cells, the B cells in the brain also should be investigated by FACS since the production of antibody by invading B cells is required for the clearance of rabies virus from the CNS (PLoS neglected tropical diseases, 2009, 10.1371/journal.pntd.0000535).*

To address this critique, we have now added flow cytometry data to support and considerably expand upon our microscopy data. Notably, even though our microscopy studies focused on the hindbrain, where the lyssavirus infection is most concentrated, FACS analysis of the entire brain demonstrated the same CD4-biased T cell infiltrate in F11-treated animals vs a group treated with an irrelevant control mAb. There is also a significant increase in T cells overall, as well as a dramatic increase in MHCII+CD11b+Ly6C- myeloid cells. By contrast, B cells, NK cells and Ly6C+ monocytes are all found at <u>lower</u> proportions in F11 treated animals (Fig 6A-D, Fig EV5A-E)

The data in Appendix Fig S1 of our study shows that F11 therapy is still fully effective in muMT- mice that lack B cells. Also, the proportion of B cells in the brain is lower in F11-

treated animals (Fig 6A). The PLOS-NTD publication cited by the reviewer is in the context of infection with an attenuated RABV strain. Although the authors did provide data arguing that B cells in the brain rather than peripherally administered Ab is important for therapeutic effect, there were many differences in their experimental design vs. ours, and it is quite difficult to compare these studies. However, we can say, based on our data mentioned above, that B cells are not important <u>for the therapeutic effect of F11</u>.

5. *CD4 T cells have been found to play a critical role against lyssavirus infection (Aurore Lebrun et al. Journal of Immunology,2015; Emily K Bongiorno, et al. Journal of Immunology,2017). According to these studies, IFN-r produced by CD4 T cells is critical for BBB opening and clearance of rabies virus invaded into the CNS. Thus, in this study the exact mechanism for CD4 T cells to clear ABLV in the brain need to be extended or at least discussed carefully.*

As requested by the reviewer, we have added these references and discussion of CD4 T cells/IFNg to the discussion.

6. *In Fig. 7, what is the mechanism for FcRγ to contribute to the therapeutic efficacy of F11? At least it should be discussed.*

Notably, because Rev 2 was concerned that the effect of the N297G mutation was rather weak, we introduced the more potent Fc mutation, LALA-PG, to the F11 mAb and found a much more striking effect (see Fig 7 – data are all new), demonstrating that Fc effector function accounts for the majority of the therapeutic effect. We have now added more discussion of potential mechanisms for FcRg involvement. However, determining the detailed mechanism of FcRg action would require many more experiments - indeed, this would represent a stand-alone study, as there are several FcRg, and we would first have to determine which one(s) participate in the therapeutic effect of F11, what cell type(s) is(are) involved, etc.

7. *It is weird to present the qPCR and IHC data by using tables (table 1-3). Using columns may be the better choice.*

We would like to address this critique. However, we are unsure of what is meant by using columns instead of tables, as our tables are simply a series of values in columns. If the editors would like this change, we would appreciate further guidance on how to comply with this request.

Minor ones:
1. *The line numbers and page numbers are missing. It is painful to point out the mistakes in the manuscript.*

Our apologies for this oversight - both sets of numbers have been added to the revision.

2. *The IACUC number in the Materials and Methods is missing.*

We have added this number. Our apologies for the oversight.

3. *In the results part, the injection routes for antibody injection or virus challenge need to be described*.

All virus injections were footpad; all antibody injections were IP. These details are stated in Methods. We can also add elsewhere in the manuscript (Results and/or Figure Legends), if the Editors support this suggested change.

Referee #2 (Comments on Novelty/Model System for Author):

N1. *The topic adressed is interesting and could have a medical impact. However, the infection model used, ABLV, is useful but the study of host immune responses in a rabies infection model, would have had a higher medical impact.*

Note that Fig 2 data are all findings with the RABV strain, CVS-11. These data show that our results are generalized to this lethal RABV strain. We used ABLV for the majority of this work, simply because this is the virus in which we incorporated the luciferase gene, enabling longitudinal tracing of progression of the viral infection - a strength of this work.

N2. *The assessment of host-immune responses deserve further investigation*.

To address this critique, we have added flow cytometry data – please see response to Rev 1 major critique 4 for details.

N*3.* *The authors state that mAb therapy has an "immune provocking" effect. However, the data presented show a role for host immune responses in mAb-mediated protection but do not allow concluding an "immune provocking" effect of mAb therapy. Such "immune provocking" or "vaccinal effects" with enhanced cellular and humoral host immune responses have been more precisely described/demonstrated in other experimental settings.*

We are unsure of the distinction being made by the reviewer. Specifically, what is the distinction between "immune provoking" and "a role for host immune responses" in the context of experiments that show (1) the adaptive immune response, particularly CD4 T cells, play an essential role in efficacy (2) simple mAb neutralization is clearly NOT the mechanism and (3) the mAb Fc region plays a major mechanistic role? From our perspective, the only conclusion that fits with these data is that F11-FcRg interactions are stimulating an immune response that results in control of infection. Thus, F11 (but not the irrelevant antibody m102.4) is provoking an immune response that does not occur when F11 is not present. Finally, we did not (and would not) use the phrase "vaccinal effects," as we agree that such an effect is not consistent with our data.

Referee #2 (Remarks for Author):

Mastraccio et al, reports an antibody-based therapeutic approach to lyssavirus infection. Using mouse models of lyssavirus infection, the authors show that administration of a neutralizing mAb after established infection protects infected mice. These results could have a potential medical impact, as lyssavirus is almost 100% fatal in symptomatically infected patients. The novelty of this work is that neutralization is not sufficient to protect animals from mortality, but that host immune responses are required to successfully control infection. However, the immune mechanisms involved may require further characterization.

Major points

1. *The authors conclude that "CD8 T cells may contribute to control of viral infection, but this population is not essential for F11-dependent survival after lyssavirus challenge." Challenge experiments were performed at 82 dpi. However, CD8 T cell depletion was performed at early post-infection time points. What about CD8 T-cell frequency before viral challenge 82 dpi? If CD8 T cell frequency was restored, the authors cannot rule out a potential role of these cells in the observed protective effect.*

We believe the reviewer is confused about the details of this particular experiment (perhaps due to our wording – our apologies if this has caused ambiguity), which is really two experiments in one. The first experiment tested the role of CD8 T cells in the protective response to F11. In these experiments, CD8 T cells were CD8-depleted (via antibody) starting 3 days prior to viral infection, resulting in a highly effective depletion through 28 days (see Appendix Fig 2). In this experiment, F11 therapy was delivered at day 5 post infection. Whereas all untreated, CD8-depleted animals succumbed to infection by day 14, all of the F11-treated CD8-depleted animals survived. This part of the experiment demonstrates that CD8 T cells are not essential for the protective effect of F11.

The *second* part of the experiment was a rechallenge of the surviving animals at day 82 post-infection. The purpose of this experiment was to evaluate whether or not the surviving animals had developed a sterilizing immune response *in the periphery*. As these animals never showed evidence of viral replication in the footpad (or anywhere else, based on bioluminescence imaging), we concluded that F11-treated animals most likely develop sterilizing immunity. Certainly, the restored CD8 T cells likely play a role in this sterilizing immunity, as may the host CD4 T cells and the B cells. But this second portion of the experiment was a minor conclusion, in comparison to the initial portion that showed that CD8 T cells are not required for F11 to protect animals from mortality (i.e., during the first 82 days, prior to rechallenge). If the reviewer still finds the second portion of this experiment (i.e., the rechallenge, only) problematic in its interpretation or execution, we are happy to remove it. The findings are not particularly important to the overall conclusions of this study.

2. *What about the humoral immune response upon viral challenge?*

Please see response to Rev 1 major critique 4 (second part) for details.

3. *While CD4 T-cell deficient mice show no long-term protection upon mAb treatment, the role of CD4 T cells in mAb-mediated protection is not well identified. Indeed, the authors show in figure 6 similar number of CD4 T cells in protected and unprotected animals. Further characterization*

of CD4 T cells (i.e. subsets, phenotypic and/or functional activation) might shed light on their role in protection observed after mAb treatment).

A similar critique was raised by Rev 1, and we added substantial flow cytometry data to address these critiques. Please see response to Rev 1 major critique 4. Also, the analysis of Tcm/Tem subsets was not particularly informative. In our opinion, the examination of T cell subsets will only be relevant if we are able to specifically identify antigen-specific cells (we do not have the required reagents at this time, and their development would represent a considerable undertaking and a stand-alone study).

4. *The "CD4-neg" population is poorly defined. CD3+ cells may also include other cell subsets (i.e. NKT cells). As the authors suggest a potential role or this population in virus-induced immunopathology, a better characterization of this population might be required using experimental approaches other than IHC. In keeping with this, further characterization of cytokines and innate immune mediators'profile in the brains of mice from different experimental groups would be informative.*

To address this critique, we included in our flow cytometry analysis strategies to identify γδ T cells and NK T cells (see Fig EV5D). While both populations are present, they do not differ in proportion between F11-treated animals and animals treated with an irrelevant mAb. Thus, CD4 T cells stand out as the primary T cell subset that differs between treated and untreated animals. With regard to cytokines/immune mediators, this is a very complex topic that we believe should be addressed in a stand-alone study, probably in the context of antigen-specific T cells, as discussed in the previous point.

5. FcRγ binding function of mAb contribution to therapeutic efficacy is mild and has also been described in other pathological contexts of rabies infection: de Melo GD, et al, A combination of two human monoclonal antibodies cures symptomatic rabies. EMBO Mol Med. 2020 Nov 6;12(11):e12628. doi: 10.15252/emmm.202012628.

We have added the de Melo citation and much discussion of their data. We were also not impressed with the effect of the N297G mutation in our study. Discussion with colleagues who study antibody effector function led to use of the more functionally crippled F11(LALA-PG) mutation, which, despite equivalent in vitro neutralization, yielded a profound reduction in the therapeutic efficacy of F11 (Fig 7, Appendix Fig S3). This aligns with the de Melo study in which they used a similar mutation (LALA) with a similar result.

6. *A few passages in the discussion are speculative:*

- "Moreover, we demonstrated that surviving, F11-treated animals exhibit sterilizing anti-lyssavirus peripheral immunity, as well as a long-lasting increase in expression of signature cellular immune response genes in the brain ». No sterilizing immunity is observed as residual viral load is detected by PCR approaches. There is not long-lasting increase in expression of signature cellular immune response genes in the brain, it is similar to that observed in early stages as well as similar to, or even lower than that observed in untreated mice.

The statement regarding sterilizing immunity refers specifically to the rechallenge of the CD8-depleted mice. In that rechallenge experiment, there was no viral replication, even at the inoculation site, hence sterilizing peripheral immunity to rechallenge of these animals. We stand by the validity of this interpretation, but we are happy to change the wording with guidance of the Editors, or remove this statement if the consensus is that the rechallenge data should be removed.

-"Therefore, the administration of F11 on day 5 or day 7 post-infection may result in delivery of viral antigen-loaded APCs in increased numbers or with higher stimulatory capacity to differentiating CD4 T cells, yielding higher numbers or altered functionality of anti- lyssavirus effector CD4 T cells ". See comment n{degree sign}3.

We agree that this section was highly speculative and of little value. It has been deleted.

-" By contrast to enhanced virus control following day 5 and day 7 therapy, administration of F11 on day 3 may deliver viral antigen to DCs too early to have a positive effect on priming, or it may deplete the infectious virus and viral antigen too efficiently to result in effective priming of the anti-lyssavirus CD4 T cell response ».

Although we find these observations worthy of discussion, we agree the statements are speculative, and they have thus been deleted.

-" our data show an immune provoking component of mAb therapy which is ultimately essential for therapeutic efficacy". The data presented show a role for host immune responses in mAb-mediated protection but do not allow concluding an "immune provoking" effect of mAb therapy.

As stated above in our response to this reviewer (Rev 2), point N3, we are confused by this critique and believe that "immune provoking" is consistent with the data. If the Editors prefer a different wording, we are happy to discuss.

Minor comments

1.*The route of administration of the mAb is not described.*

In Materials and Methods, in mAb treatment, we state: mAbs were administered intraperitoneally at a dose of 10 mg/kg.

2*.* *In figure 3, panel F, the time point of coronal sections is not indicated. Same for Fig S1C.*

The requested information has been added to the legends for Figs 2F and EV1C.

3*.* *The reason why albino mice are used in some experiments and not in others is not explained.*

This is explained in Mastraccio et al, 2020, which is cited throughout the manuscript. Albino mice can be used directly for luciferase imaging. Black mice must be shaved or have hair removed in some other manner. Even post-hair removal, the luciferase signal is attenuated in black-pigmented mice.

4. *Correct in the introduction "To date, there has been no small molecule- or biologic-based therapeutic approach demonstrated to reliably treat CNS-resident lyssavirus infection,*

preventing mortality" (see reference De Melo, 2020 above mentioned).
This statement has been corrected and this important reference has been added.

5. *Define PEP, in the introduction*
Correction made.

Referee #3 (Comments on Novelty/Model System for Author):

please see my comments to the authors below

Referee #3 (Remarks for Author):

Major point of critiques:

1. *"Infections with rabies virus (RABV) and related lyssaviruses are uniformly fatal" - are you talking about people here? Because infections with rabies virus (RABV) and related lyssaviruses in wild animals are NOT uniformly fatal.*

This statement is in the Abstract which is limited to 175 words. If the Editors can allow more words, we can make this statement more precise, by adding "of humans."

2. *"...are uniformly fatal, once virus accesses the central nervous system (CNS)" - totally incorrect! Only after clinical symptoms developed. Fix it over the entire text.*

We believe we have made the requested clarifications across the entire text.

3. *"...and reverses disease signs" - be clear which signs of rabies have been reversed.*

The disease signs that are measured are clearly indicated in the Methods. The results clearly state that all disease signs are reversed in the majority of animals.

4. *"...cause encephalitic clinical symptoms (Olival & Daszak, 2005)." - or neurological clinical symptoms?*

The reviewer appears to prefer "neurological," so we have made this change.

5. *It is not clear why the authors emphasized bat-borne rabies in the second paragraph of the introduction. The main source of human rabies in the world is rabid dogs (most importantly!) and other carnivores (much less!), not bats.*

We describe bat-borne lyssavirus because we use ABLV (a bat-borne lyssavirus) for the majority of this study. Hence, this section of introduction is essential.

6. *Please indicate all abbreviations in the text the first time they are used. Fix it over the entire text.*

We believe we have fixed all such omissions, but would ask that the Editors inform us of any that we missed.

7. *"...and exposure site injection of polyspecific human or equine rabies immunoglobulin (HRIG or ERIG, respectively)" - incorrect! HRIG or ERIG are not always indicated!*

We have added "in the case of bite wounds" for accuracy.

8. *"mAbs may have therapeutic value, administration of mAbs was performed prior to or within 2 hours of RABV infection" - I think you are talking about mouse model here. Please indicate it in the text. It is not a human or NHP data.*

We have inserted "animal" studies for clarity. Both mice and hamsters were used in the cited studies.

9. The Introduction is too long and can be cut in half. The last paragraph of the Introduction should be moved to Discussion.

We will follow the guidance of the editors regarding any required changes to our revised manuscript.

10. Why outbred mice, commonly used in the rabies NIH test, not used here? Why special inbred strains were used?

The choice of inbred mice for this model is due to our longer-term plans to define the immunological mechanism of therapeutic efficacy in this model - hence, as strain with a single MHC haplotype is needed. The use of C57BL/6-based strains (such as ours) are common in the literature. The de Melo study mentioned by both Rev 1 and Rev 2 employed a different inbred strain, BALB/c.

11. *"For rabies challenge studies, a rodent-adapted RABV strain (CVS-11) was used (ATCC Cat#VR-959)." - how it was grown and prepared for the study?*

As per the instructions supplied by ATCC, CVS-11 was grown in BHK21, clone 13. This information is now added to Methods.

12. It is not clear how many TCID50 of ABLV-luc or CVS-11 were used in In vitro neutralization assays? Normally, it should be between 50-100 TCID50 per the tested antibody dilution. The potency of your mAb F11 should be assessed in the conventional RFFIT test.

The potency of F11 is thoroughly addressed in our previous publication, Weir et al, (2021). Viruses 13: 391, cited in the text. The neutralization assays employed in that publication are commonly used.

13. *Very unusual challenge method is used - in rear footpad! I don't think that it is equivalent to commonly used challenge methods for laboratory rodents - IC and IN. Second, there is no data provided on validity of this challenge method and challenge model. I'm not sure if the same results with your F11 mAb will be shown in outbred mice infected with rabies using the conventional IC and IN infection methods. In my opinion, an incorrect, mild model was selected*.

The statement that hind limb inoculation is "unusual" is simply untrue for the lyssavirus mouse model, in which inoculation of the rear gastrocnemius muscle or rear footpad are both common, because they mimic the major natural route of infection of humans (dog bite of a peripheral limb, in which replication occurs first in muscle tissue, followed by progression to peripheral nerves, then the spinal cord and finally to the brain). The footpad inoculation challenge model has been used extensively in the literature since the 1970s, with some of the best characterization of this model coming from the work of Dr. Alan C. Jackson (e.g., https://journals.asm.org/doi/full/10.1128/JVI.01677-07; his contributions to the rabies field have been extensive: https://umanitoba.ca/faculties/health_sciences/medicine/units/intmed/about/jackson.html). Thus, the footpad inoculation model is validated by 50 years of published peer-reviewed literature.

By contrast, IC inoculation is not a proxy for any natural exposure route; nor would it be for a therapeutic approach using a monoclonal antibody delivered systemically. The NIH IC inoculation protocol has historically been used in many vaccine studies, as it provides the most rapid possible infection of the CNS, and a rapid assessment regarding whether a vaccine is capable of protecting against CNS infection. However, we are *not* describing a vaccine - we are describing a *therapy for established infection*. The use of IC inoculation is not appropriate for a therapy study, particularly given the mechanism of protection suggested by our data, in which exposure/priming of host T cells to viral antigen *in the periphery* is almost certainly a required component for therapeutic efficacy. Indeed, the very nice 2020 de Melo et al EMBO Molecular Medicine article, cited by Revs 1 and 2 (EMBO Mol Med (2020) 12: e12628) exclusively employed inoculation in the gastrocnemius muscle of the rear legs for their study, which described a different antibody-based therapeutic approach.

Regarding, "_no data provided on validity of this . . . challenge model_**," extensive data on the model are provided in Mastraccio KE, et al (2020) Establishment of a longitudinal pre-clinical model of lyssavirus infection. *Journal of virological methods* 281: 113882, which is cited throughout this manuscript.**

Finally, in the absence of treatment, our footpad infections are 100% lethal, so concrete data stand in contradiction to the assertion that this model is "mild."

14. *What was the sensitivity (in rabies virus copies in the CNS tissues) of your In vivo bioluminescence imaging? I'm not sure that this bioluminescence imaging is very sensitive for detection of rabies virus replication in the CNS!*

The sensitivity is demonstrated in our earlier publication that we cite many times (Mastraccio KE, et al (2020) *Journal of virological methods* 281: 113882). Although low viral loads (measured by qPCR as ~10e4 copies of N sense strand RNA transcripts per ug of total brain RNA) cannot be reliably detected by bioluminescence imaging, we show in that publication that there is a very strong correlation between bioluminescence intensity and viral load as measured by qPCR in the brain at 10e5 copies of N/ug total RNA and higher. In this study, the low level of transcriptionally active ABLV maintained in the brains of F11-treated animals (undetectable by bioluminescent imaging) is quantified by qPCR in Fig. 6G and Table 2.

15. *Why brain sections were stained with mouse anti-rabies G (clone 1C5, Novus Biologicals Cat#NBP2-11630) overnight at 4{degree sign}C? It is very unusual protocol!*

We have found that overnight incubation at 4C gives better signal-to-noise than ambient temperature incubation for 30-60 min. This is true for most antibodies that we have tested. Although the protocol may be unusual, we employ it because it gives better results.

16. The "Results" section is too wordy, and many paragraphs looks like the "Discussion" section.

We have written the results in a manner that we believe describes the data as clearly and completely as possible. We will follow the guidance of the Editors regarding any structural changes to our revised manuscript.

17. *The Discussion is too long and can be cut in half.*

We have edited the Discussion substantially, but we have also needed to add text to describe the new data and to fully respond to all reviewer concerns, which included adding references and *more* discussion. We will follow the guidance of the editors regarding any required changes to our revised manuscript

18. The originality of this study in respect that T cell-dependent immune response is required for successful control of rabies infection in the CNS is questionable, because it was previously demonstrated in several publications (as examples: J Immunol. 2015 Nov 1; 195(9): 4358-4368; J Virol . 2018 Mar 28;92(8):e02029-17; PLoS Negl Trop Dis. 2015 Aug 20;9(8):e0004023; J Virol. 2001 Apr;75(7):3427-34.)

We believe that the reviewer is failing to appreciate the central focus of our manuscript, which is mechanisms of immunity specifically *<u>induced by antibody therapy</u>*. The above studies focus on the following:

J Immunol. 2015 Nov 1; 195(9): 4358-4368 – immune mechanisms of clearance of an attenuated rabies strain.

J Virol . 2018 Mar 28;92(8):e02029-17 – immune mechanisms of clearance of an attenuated rabies strain.

PLoS Negl Trop Dis. 2015 Aug 20;9(8):e0004023 – comparison of immune responses induced by an attenuated RABV vs. a highly pathogenic wt RABV

J Virol. 2001 Apr;75(7):3427-34 - Assessment of the role of T cells following rabies virus ocular disease

In short, none of the above studies involve antibody therapy, which is the main focus of our work. More specifically, our manuscript is focused on how this particular antibody therapy changes the host immune response in a manner that protects infected animals from mortality. The only comparable study of which we are aware is the above-mentioned study by de Melo. The novelty of our work in comparison to that publication is that we employ only peripherally administered mAb, whereas they employ both peripheral and CNS-administered mAb. They use continuous infusion in the CNS, whereas we use only a single dose peripherally. Each study has its strengths, and we believe that both studies will serve as catalysts for many interesting and important follow-up studies in rabies therapy and other antibody-based antiviral therapies.

11th Aug 2023

Dear Dr. Schaefer,

Thank you for the submission of your revised manuscript to EMBO Molecular Medicine. I am pleased to inform you that we will be able to accept your manuscript pending the following final amendments:

1) Please shorten the last paragraph of the introduction. It should include the aim and the main conclusions of the study.
2) Please address all the minor points raised by the referees, particular attention should be given to the points concerning interpretation of the data.
3) Authors: E-mail correspondence to Austin Feasly could not be delivered. Please update his e-mail address and make sure to enter correct e-mail addresses for all authors in our submission system.
4) In the main manuscript file, please do the following:
- Correct/answer the track changes suggested by our data editors by working from the attached document.
- Add up to 5 keywords.
- Add callouts for Fig 7B and Fig EV2 A-F.
- In M&M, provide the antibody dilutions that were used for each antibody.
- Please rename "Competing Interest" to "Disclosure Statement & Competing Interests". We updated our journal's competing interests policy in January 2022 and request authors to consider both actual and perceived competing interests. Please review the policy https://www.embopress.org/competing-interests and update your competing interests if necessary.
- Author contributions: Please remove it from the manuscript and specify author contributions in our submission system. CRediT has replaced the traditional author contributions section because it offers a systematic machine-readable author contributions format that allows for more effective research assessment. You are encouraged to use the free text boxes beneath each contributing author's name to add specific details on the author's contribution. More information is available in our guide to authors:
https://www.embopress.org/page/journal/17574684/authorguide#authorshipguidelines
- Please remove "Data and materials availability" and only leave "Data availability" section.
5) Appendix: Please rename appendix figures to "Appendix Figure S1" etc. and update their callouts in the main manuscript text.
6) Synopsis: Every published paper now includes a 'Synopsis' to further enhance discoverability. Synopses are displayed on the journal webpage and are freely accessible to all readers. They include separate synopsis image and synopsis text.
- Synopsis image: Please provide the visual abstract as a high-resolution jpeg file 550 px-wide x (250-400)-px high.
- Synopsis text: Please provide a short standfirst (maximum of 300 characters, including space) as well as 2-5 one sentence bullet points that summarise the paper as a .doc file. Please write the bullet points to summarise the key NEW findings. They should be designed to be complementary to the abstract - i.e. not repeat the same text. We encourage inclusion of key acronyms and quantitative information (maximum of 30 words / bullet point). Please use the passive voice.
- Please check your synopsis text and image before submission with your revised manuscript. Please be aware that in the proof stage minor corrections only are allowed (e.g., typos).
7) For more information: This space should be used to list relevant web links for further consultation by our readers. Could you identify some relevant ones and provide such information as well? Some examples are patient associations, relevant databases, OMIM/proteins/genes links, author's websites, etc...
8) As part of the EMBO Publications transparent editorial process initiative (see our Editorial at http://embomolmed.embopress.org/content/2/9/329), EMBO Molecular Medicine will publish online a Review Process File (RPF) to accompany accepted manuscripts. This file will be published in conjunction with your paper and will include the anonymous referee reports, your point-by-point response and all pertinent correspondence relating to the manuscript. Let us know whether you agree with the publication of the RPF and as here, if you want to remove or not any figures from it prior to publication. Please note that the Authors checklist will be published at the end of the RPF.
9) Please provide a point-by-point letter INCLUDING my comments as well as the reviewer's reports and your detailed responses (as Word file).

I look forward to reading a new revised version of your manuscript as soon as possible.

Yours sincerely,

Zeljko Durdevic

Zeljko Durdevic
Editor
EMBO Molecular Medicine

*** Instructions to submit your revised manuscript ***

*** PLEASE NOTE *** As part of the EMBO Publications transparent editorial process initiative (see our Editorial at https://www.embopress.org/doi/pdf/10.1002/emmm.201000094), EMBO Molecular Medicine will publish online a Review Process File to accompany accepted manuscripts.

In the event of acceptance, this file will be published in conjunction with your paper and will include the anonymous referee reports, your point-by-point response and all pertinent correspondence relating to the manuscript. If you do NOT want this file to be published, please inform the editorial office at contact@embomolmed.org.

When submitting your revised manuscript, please include:

1) a .docx formatted version of the manuscript text (including Figure legends and tables)

2) Separate figure files*

3) supplemental information as Expanded View and/or Appendix. Please carefully check the authors guidelines for formatting Expanded view and Appendix figures and tables at https://www.embopress.org/page/journal/17574684/authorguide#expandedview

4) a letter INCLUDING the reviewer's reports and your detailed responses to their comments (as Word file).

5) The paper explained: EMBO Molecular Medicine articles are accompanied by a summary of the articles to emphasize the major findings in the paper and their medical implications for the non-specialist reader. Please provide a draft summary of your article highlighting
- the medical issue you are addressing,
- the results obtained and
- their clinical impact.
This may be edited to ensure that readers understand the significance and context of the research.
Please refer to any of our published articles for an example.

6) For more information: There is space at the end of each article to list relevant web links for further consultation by our readers. Could you identify some relevant ones and provide such information as well? Some examples are patient associations, relevant databases, OMIM/proteins/genes links, author's websites, etc...

7) Author contributions: the contribution of every author must be detailed in a separate section.

8) EMBO Molecular Medicine now requires a complete author checklist (https://www.embopress.org/page/journal/17574684/authorguide) to be submitted with all revised manuscripts. Please use the checklist as guideline for the sort of information we need WITHIN the manuscript. The checklist should only be filled with page numbers were the information can be found. This is particularly important for animal reporting, antibody dilutions (missing) and exact values and n that should be indicted instead of a range.

9) Every published paper now includes a 'Synopsis' to further enhance discoverability. Synopses are displayed on the journal webpage and are freely accessible to all readers. They include a short stand first (maximum of 300 characters, including space) as well as 2-5 one sentence bullet points that summarise the paper. Please write the bullet points to summarise the key NEW findings. They should be designed to be complementary to the abstract - i.e. not repeat the same text. We encourage inclusion of key acronyms and quantitative information (maximum of 30 words / bullet point). Please use the passive voice. Please attach these in a separate file or send them by email, we will incorporate them accordingly.

You are also welcome to suggest a striking image or visual abstract to illustrate your article. If you do please provide a jpeg file 550 px-wide x 400-px high.

10) A Conflict of Interest statement should be provided in the main text

11) Please note that we now mandate that all corresponding authors list an ORCID digital identifier. This takes <90 seconds to

complete. We encourage all authors to supply an ORCID identifier, which will be linked to their name for unambiguous name identification.

Currently, our records indicate that the ORCID for your account is 0000-0001-8877-3507.

Please click the link below to modify this ORCID:
Link Not Available

12) The system will prompt you to fill in your funding and payment information. This will allow Wiley to send you a quote for the article processing charge (APC) in case of acceptance. This quote takes into account any reduction or fee waivers that you may be eligible for. Authors do not need to pay any fees before their manuscript is accepted and transferred to our publisher.

*Additional important information regarding Figures

Each figure should be given in a separate file and should have the following resolution:
Graphs 800-1,200 DPI
Photos 400-800 DPI
Colour (only CMYK) 300-400 DPI"

Figures are not edited by the production team. All lettering should be the same size and style; figure panels should be indicated by capital letters (A, B, C etc). Gridlines are not allowed except for log plots. Figures should be numbered in the order of their appearance in the text with Arabic numerals. Each Figure must have a separate legend and a caption is needed for each panel.

*Additional important information regarding figures and illustrations can be found at https://bit.ly/EMBOPressFigurePreparationGuideline. See also figure legend preparation guidelines: https://www.embopress.org/page/journal/17574684/authorguide#figureformat

The system will prompt you to fill in your funding and payment information. This will allow Wiley to send you a quote for the article processing charge (APC) in case of acceptance. This quote takes into account any reduction or fee waivers that you may be eligible for. Authors do not need to pay any fees before their manuscript is accepted and transferred to our publisher.

***** Reviewer's comments *****

Referee #1 (Comments on Novelty/Model System for Author):

The mouse model is adequate for this study.

Referee #1 (Remarks for Author):

In this revised the version, the authors have added more data to support their claims. Also, they have improved the manuscript a lot according to the suggestions from the reviewers. I am satisfied with the most of the responses from the authors. But there are still several concerns need to be mentioned as below:
(1)The authors added flow cytometry data as suggested in the new Fig. 6. However, the pattern of Fig. 6C is quite different from others, which should be unified. In Fig. 6F, the statistics is missing.
(2)In Fig. EV5, the resolution of the flow cytometry plots is not high enough to read.
(3)In the supplemental figures (S1-S3), the sample sizes in the figure legends are missing.

Referee #2 (Comments on Novelty/Model System for Author):

Previously mentioned in the first review.

Referee #2 (Remarks for Author):

The authors have answered most of the questions raised.

However, on some specific points, I still disagree with the authors'comments/interpretations:

1. "Specifically, what is the distinction between "immune provoking" and "a role for host immune responses" in the context of experiments that show (1) the adaptive immune response, particularly CD4 T cells, play an essential role in efficacy (2) simple mAb neutralization is clearly NOT the mechanism and (3) the mAb Fc region plays a major mechanistic role? From our

perspective, the only conclusion that fits with these data is that F11-FcRg interactions are stimulating an immune response that results in control of infection

This statement "the only conclusion that fits with these data is that F11-FcRg interactions are stimulating an immune response that results in control of infection" is wrong. This is not demonstrated at all: the experiments using the LALA-PG mutant only show a decreased protection, but NO data is shown on the effect of this mutant on the stimulation ofimmune response. Fc-FcgR interactions might be simply necessary to recruit effector cells that contribute to viral control. NO evidence of enhancement of immune responses in a Fc-dependent manner is shown.

2 . The authors cannot conclude that "sterilizing immunity" has been induced on the basis of Figure 4. The sensitivity of bioluminescent imaging is not high enough. In addition, the authors describe viral persistence detected by PCR approaches. "Protective immunity" would be more appropriate.

Responses to editors and reviewers (revised manuscript review) – We thank the editors and reviewers for their comments to improve the manuscript. Author responses in bold type; manuscript changes made on our end indicated by tracked changes in the uploaded Word doc:

I am pleased to inform you that we will be able to accept your manuscript pending the following final amendments:

1) Please shorten the last paragraph of the introduction. It should include the aim and the main conclusions of the study.

The length of this paragraph has been cut approximately in half.

2) Please address all the minor points raised by the referees, particular attention should be given to the points concerning interpretation of the data.

Completed as requested.

3) Authors: E-mail correspondence to Austin Feasly could not be delivered. Please update his e-mail address and make sure to enter correct e-mail addresses for all authors in our submission system.

Email has been updated.

4) In the main manuscript file, please do the following:

- Correct/answer the track changes suggested by our data editors by working from the attached document.

Done.

- Add up to 5 keywords.

Done.

- Add callouts for Fig 7B and Fig EV2 A-F.

Done.

- In M&M, provide the antibody dilutions that were used for each antibody.

Done.

- Please rename "Competing Interest" to "Disclosure Statement & Competing Interests". We updated our journal's competing interests policy in January 2022 and request authors to consider both actual and perceived competing interests. Please review the policy https://www.embopress.org/competing-interests and update your competing interests if necessary.

Done.

- Author contributions: Please remove it from the manuscript and specify author contributions in our submission system. CRediT has replaced the traditional author contributions section because it offers a systematic machine-readable author contributions format that allows for more effective research assessment. You are encouraged to use the free text boxes beneath

each contributing author's name to add specific details on the author's contribution. More information is available in our guide to authors:

https://www.embopress.org/page/journal/17574684/authorguide#authorshipguidelines

Done.

- Please remove "Data and materials availability" and only leave "Data availability" section.

I have a question about this one. We uploaded the original microscopy and flow cytometry data - so our statement needs to be updated somehow. I have made an attempt, but this may need to be revised.

5) Appendix: Please rename appendix figures to "Appendix Figure S1" etc. and update their callouts in the main manuscript text.

Appendix Figures have been renamed. Callouts were in the proper format.

6) Synopsis: Every published paper now includes a 'Synopsis' to further enhance discoverability. Synopses are displayed on the journal webpage and are freely accessible to all readers. They include separate synopsis image and synopsis text.

- Synopsis image: Please provide the visual abstract as a high-resolution jpeg file 550 px-wide x (250-400)-px high.

- Synopsis text: Please provide a short standfirst (maximum of 300 characters, including space) as well as 2-5 one sentence bullet points that summarise the paper as a .doc file. Please write the bullet points to summarise the key NEW findings. They should be designed to be complementary to the abstract - i.e. not repeat the same text. We encourage inclusion of key acronyms and quantitative information (maximum of 30 words / bullet point). Please use the passive voice.

- Please check your synopsis text and image before submission with your revised manuscript. Please be aware that in the proof stage minor corrections only are allowed (e.g., typos).

Both items have been completed and included in our upload.

7) For more information: This space should be used to list relevant web links for further consultation by our readers. Could you identify some relevant ones and provide such information as well? Some examples are patient associations, relevant databases, OMIM/proteins/genes links, author's websites, etc...

Relevant databases:

https://ictv.global/report/chapter/rhabdoviridae/rhabdoviridae/lyssavirus

https://www.who.int/news-room/fact-sheets/detail/rabies

https://www.woah.org/app/uploads/2018/06/zero-by-30-final-online-version.pdf

Author websites:

https://www.researchgate.net/profile/Brian-Schaefer-5

https://www.researchgate.net/profile/Christopher-Broder

8) As part of the EMBO Publications transparent editorial process initiative (see our Editorial at http://embomolmed.embopress.org/content/2/9/329), EMBO Molecular Medicine will publish online a Review Process File (RPF) to accompany accepted manuscripts. This file will be published in conjunction with your paper and will include the anonymous referee reports, your point-by-point response and all pertinent correspondence relating to the manuscript. Let us know whether you agree with the publication of the RPF and as here, if you want to remove or not any figures from it prior to publication. Please note that the Authors checklist will be published at the end of the RPF.

No concerns.

9) Please provide a point-by-point letter INCLUDING my comments as well as the reviewer's reports and your detailed responses (as Word file).

This document.

****** Reviewer's comments ******

Referee #1 (Comments on Novelty/Model System for Author):

The mouse model is adequate for this study.

Referee #1 (Remarks for Author):

In this revised the version, the authors have added more data to support their claims. Also, they have improved the manuscript a lot according to the suggestions from the reviewers. I am satisfied with the most of the responses from the authors. But there are still several concerns need to be mentioned as below:
(1)The authors added flow cytometry data as suggested in the new Fig. 6. However, the pattern of Fig. 6C is quite different from others, which should be unified. In Fig. 6F, the statistics is missing.

Fig 6C has been changed as requested. For 6F, the number of samples in each group is quite small, and the variance in values in the infected, untreated is quite high, which is why statistical analysis was not performed. At this stage in the review process, it does not seem appropriate for us to add more data points (which would be required for meaningful statistics with this kind of dataset). However, we will follow the guidance of the editors on this issue. Overall, we think this panel plus panel 6G provides ample evidence that treatment reduces ABLV N copy number.

(2)In Fig. EV5, the resolution of the flow cytometry plots is not high enough to read.

In the submitted version of Fig EV5, all text can be read upon zooming. We suspect that during the manuscript assembly process on the EMBO server, there was some loss of resolution. We will work with the editors to ensure that EV5 remains readable in the final version.

(3) In the supplemental figures (S1-S3), the sample sizes in the figure legends are missing.

Appendix Figure S1A states "n=6 mice/group, n=3 Uninfected, Untreated mice/group" – this applies to all panels. The information regarding sample sized for S2 and S3 has now been added.

Referee #2 (Comments on Novelty/Model System for Author):

Previously mentioned in the first review.

Referee #2 (Remarks for Author):

The authors have answered most of the questions raised.

However, on some specific points, I still disagree with the authors'comments/interpretations:

1. "Specifically, what is the distinction between "immune provoking" and "a role for host immune responses" in the context of experiments that show (1) the adaptive immune response, particularly CD4 T cells, play an essential role in efficacy (2) simple mAb neutralization is clearly NOT the mechanism and (3) the mAb Fc region plays a major mechanistic role? From our perspective, the only conclusion that fits with these data is that F11-FcRg interactions are stimulating an immune response that results in control of infection

This statement "the only conclusion that fits with these data is that F11-FcRg interactions are stimulating an immune response that results in control of infection" is wrong. This is not demonstrated at all: the experiments using the LALA-PG mutant only show a decreased protection, but NO data is shown on the effect of this mutant on the stimulation of immune response. Fc-FcgR interactions might be simply necessary to recruit effector cells that contribute to viral control. NO evidence of enhancement of immune responses in a Fc-dependent manner is shown.

The reviewer has expressed a difference in opinion regarding a statement made in our Responses to Reviewers (and a data interpretation made in our Discussion). Although our opinion is that "recruit[ment] of effector cells" is equivalent to "enhancement of immune responses," (as immune cell recruitment is an activity that would not have existed without the therapeutic antibody), we acknowledge the views of this reviewer, and see no point in arguing further. We will thus make changes in accordance with the Reviewer's critique. Our understanding of the thrust of this (and the original) critique is that the reviewer objects to the following statement in the Discussion: "Similar to other recent studies mentioned above, our data show an <u>immune provoking component</u> of mAb therapy which is ultimately essential for therapeutic efficacy." We have now changed this statement to, "Similar to other recent studies mentioned above, our data show that mAb therapy involves Fc-FcRγ interactions that are ultimately essential for therapeutic efficacy." There are no further instances of "immune provoking" in the current version of the manuscript, and we therefore believe we have fully addressed this

critique. However, if the reviewer believes that there are highly similar interpretive statements that also require modification, please identify these specifically.

2 The authors cannot conclude that "sterilizing immunity" has been induced on the basis of Figure 4. The sensitivity of bioluminescent imaging is not high enough. In addition, the authors describe viral persistence detected by PCR approaches. "Protective immunity" would be more appropriate.

Our apologies – on the basis of the prior review, our intention was to change all instances of "sterilizing immunity" to "protective immunity." We did this throughout results, but missed this instance in the discussion. The sentence in the discussion has now been changed to "protective immune response."

25th Aug 2023

Dear Dr. Schaefer,

We are pleased to inform you that your manuscript is accepted for publication and is now being sent to our publisher to be included in the next available issue of EMBO Molecular Medicine.

Please read below for additional IMPORTANT information regarding your article, its publication and the production process.

Congratulations on your interesting work,

Zeljko Durdevic

Zeljko Durdevic
Editor
EMBO Molecular Medicine

Follow us on Twitter @EmboMolMed
Sign up for eTOCs at embopress.org/alertsfeeds

*** *** *** IMPORTANT INFORMATION *** *** ***

SPEED OF PUBLICATION
The journal aims for rapid publication of papers, using using the advance online publication "Early View" to expedite the process: A properly copy-edited and formatted version will be published as "Early View" after the proofs have been corrected. Please help the Editors and publisher avoid delays by providing e-mail address(es), telephone and fax numbers at which author(s) can be contacted.

Should you be planning a Press Release on your article, please get in contact with embomolmed@wiley.com as early as possible, in order to coordinate publication and release dates.

LICENSE AND PAYMENT:

All articles published in EMBO Molecular Medicine are fully open access: immediately and freely available to read, download and share.

EMBO Molecular Medicine charges an article processing charge (APC) to cover the publication costs. You, as the corresponding author for this manuscript, should have already received a quote with the article processing fee separately. Please let us know in case this quote has not been received.

Once your article is at Wiley for editorial production you will receive an email from Wiley's Author Services system, which will ask you to log in and will present you with the publication license form for completion. Within the same system the publication fee can be paid by credit card, an invoice, pro forma invoice or purchase order can be requested.

Payment of the publication charge and the signed Open Access Agreement form must be received before the article can be published online.

PROOFS

You will receive the proofs by e-mail approximately 2 weeks after all relevant files have been sent o our Production Office. Please return them within 48 hours and if there should be any problems, please contact the production office at embopressproduction@wiley.com.

Please inform us if there is likely to be any difficulty in reaching you at the above address at that time. Failure to meet our

deadlines may result in a delay of publication.

All further communications concerning your paper proofs should quote reference number EMM-2022-16394-V3 and be directed to the production office at embopressproduction@wiley.com.

Thank you,

Zeljko Durdevic
Editor
EMBO Molecular Medicine

EMBO Press Author Checklist

Corresponding Author Name: Brian C Schaefer, PhD
Journal Submitted to: EMBO Molecular Medicine
Manuscript Number: EMM-2022-16394-V2

USEFUL LINKS FOR COMPLETING THIS FORM
The EMBO Journal - Author Guidelines
EMBO Reports - Author Guidelines
Molecular Systems Biology - Author Guidelines
EMBO Molecular Medicine - Author Guidelines

Reporting Checklist for Life Science Articles (updated January
This checklist is adapted from Materials Design Analysis Reporting (MDAR) Checklist for Authors. MDAR establishes a minimum set of requirements in transparent reporting in the life sciences (see Statement of Task: 10.31222/osf.io/9sm4x). Please follow the journal's guidelines in preparing your manuscript.
Please note that a copy of this checklist will be published alongside your article.

Abridged guidelines for figures
1. Data
The data shown in figures should satisfy the following conditions:
- the data were obtained and processed according to the field's best practice and are presented to reflect the results of the experiments in an accurate and unbiased manner.
- ideally, figure panels should include only measurements that are directly comparable to each other and obtained with the same assay.
- plots include clearly labeled error bars for independent experiments and sample sizes. Unless justified, error bars should not be shown for technical
- if n<5, the individual data points from each experiment should be plotted. Any statistical test employed should be justified.
- Source Data should be included to report the data underlying figures according to the guidelines set out in the authorship guidelines on Data

2. Captions
Each figure caption should contain the following information, for each panel where they are relevant:
- a specification of the experimental system investigated (eg cell line, species name).
- the assay(s) and method(s) used to carry out the reported observations and measurements.
- an explicit mention of the biological and chemical entity(ies) that are being measured.
- an explicit mention of the biological and chemical entity(ies) that are altered/varied/perturbed in a controlled manner.
- the exact sample size (n) for each experimental group/condition, given as a number, not a range;
- a description of the sample collection allowing the reader to understand whether the samples represent technical or biological replicates (including how many animals, litters, cultures, etc.).
- a statement of how many times the experiment shown was independently replicated in the laboratory.
- definitions of statistical methods and measures:
  - common tests, such as t-test (please specify whether paired vs. unpaired), simple χ2 tests, Wilcoxon and Mann-Whitney tests, can be unambiguously identified by name only, but more complex techniques should be described in the methods section;
  - are tests one-sided or two-sided?
  - are there adjustments for multiple comparisons?
  - exact statistical test results, e.g., P values = x but not P values < x;
  - definition of 'center values' as median or average;
  - definition of error bars as s.d. or s.e.m.

Please complete ALL of the questions below.
Select "Not Applicable" only when the requested information is not relevant for your study.

Materials

Newly Created Materials	Information included in the manuscript?	In which section is the information available? (Reagents and Tools Table, Materials and Methods, Figures, Data Availability Section)
New materials and reagents need to be available; do any restrictions apply?	Yes	Some materials require an MTA. All materials are described (or mentioned with references to original description) in Materials and Methods

Antibodies	Information included in the manuscript?	In which section is the information available? (Reagents and Tools Table, Materials and Methods, Figures, Data Availability Section)
For **antibodies** provide the following information:<br>- Commercial antibodies: RRID (if possible) or supplier name, catalogue number and or/clone number<br>- Non-commercial: RRID or citation	Yes	All materials are described with cat#s and/or RRIDs in Materials and Methods

DNA and RNA sequences	Information included in the manuscript?	In which section is the information available? (Reagents and Tools Table, Materials and Methods, Figures, Data Availability Section)
**Short novel DNA or RNA including primers, probes**: provide the sequences.	Yes	Listed in Materials and Methods

Cell materials	Information included in the manuscript?	In which section is the information available? (Reagents and Tools Table, Materials and Methods, Figures, Data Availability Section)
**Cell lines:** Provide species information, strain. Provide accession number in repository **OR** supplier name, catalog number, clone number, and/**OR** RRID.	Yes	Listed in Materials and Methods
**Primary cultures:** Provide species, strain, sex of origin, genetic modification status.	Not Applicable	
Report if the cell lines were recently **authenticated** (e.g., by STR profiling) and tested for mycoplasma contamination.	Yes	Cell lines were purchased from ATCC and stored as early passage stocks from that initial freeze - thus, required authentication was performed by ATCC

Experimental animals	Information included in the manuscript?	In which section is the information available? (Reagents and Tools Table, Materials and Methods, Figures, Data Availability Section)
**Laboratory animals or Model organisms:** Provide species, strain, sex, age, genetic modification status. Provide accession number in repository **OR** supplier name, catalog number, clone number, **OR** RRID.	Yes	All strains are commercially available or are crosses of commercially available strains. All materials are described with cat#s and/or RRIDs in Materials and Methods.
**Animal observed in or captured from the field:** Provide species, sex, and age where possible.	Not Applicable	
Please detail **housing and husbandry conditions**.	Yes	Animals were housed either at our Charles River Laboratories breeding colony or within our University vivarium. Both are AAALAC-accredited SPF

Plants and microbes	Information included in the manuscript?	In which section is the information available? (Reagents and Tools Table, Materials and Methods, Figures, Data Availability Section)
**Plants:** provide species and strain, ecotype and cultivar where relevant, unique accession number if available, and source (including location for collected wild specimens).	Not Applicable	
**Microbes:** provide species and strain, unique accession number if available, and source.	Not Applicable	

Human research participants	Information included in the manuscript?	In which section is the information available? (Reagents and Tools Table, Materials and Methods, Figures, Data Availability Section)
If collected and within the bounds of privacy constraints report on age, sex and gender or ethnicity for all study participants.	Not Applicable	

Core facilities	Information included in the manuscript?	In which section is the information available? (Reagents and Tools Table, Materials and Methods, Figures, Data Availability Section)
If your work benefited from core facilities, was their service mentioned in the acknowledgments section?	Yes	Acknowledgments

Design

Study protocol	Information included in the manuscript?	In which section is the information available? (Reagents and Tools Table, Materials and Methods, Figures, Data Availability Section)
If study protocol has been **pre-registered, provide DOI in the manuscript**. For clinical trials, provide the trial registration number **OR** cite DOI.	Not Applicable	
Report the **clinical trial registration number** (at ClinicalTrials.gov or equivalent), where applicable.	Not Applicable	

Laboratory protocol	Information included in the manuscript?	In which section is the information available? (Reagents and Tools Table, Materials and Methods, Figures, Data Availability Section)
Provide DOI OR other citation details if **external detailed step-by-step protocols** are available.	Yes	References: Mastraccio et al (2022) DOI: 10.1007/978-1-0716-2453-1_30

Experimental study design and statistics	Information included in the manuscript?	In which section is the information available? (Reagents and Tools Table, Materials and Methods, Figures, Data Availability Section)
Include a statement about **sample size** estimate even if no statistical methods were used.	Not Applicable	
Were any steps taken to minimize the effects of subjective bias when allocating animals/samples to treatment (e.g. **randomization procedure**)? If yes, have they been described?	Yes	Where possible, samples for analysis were analyzed in a blinded fashion. This is explicitly stated in Materials and Methods.
Include a statement about **blinding** even if no blinding was done.	Yes	Where applicable.
Describe **inclusion/exclusion criteria** if samples or animals were excluded from the analysis. Were the criteria pre-established?<br><br>If sample or data points were omitted from analysis, report if this was due to attrition or intentional exclusion and provide justification.	Not Applicable	No data were excluded
For every figure, are **statistical tests** justified as appropriate? Do the data meet the assumptions of the tests (e.g., normal distribution)? Describe any methods used to assess it. Is there an estimate of variation within each group of data? Is the variance similar between the groups that are being statistically compared?	Yes	Statistical methods and corrections for multiple testing are described in Materials and Methods and/or Figure Legends

Sample definition and in-laboratory replication	Information included in the manuscript?	In which section is the information available? (Reagents and Tools Table, Materials and Methods, Figures, Data Availability Section)
In the figure legends: state number of times the experiment was **replicated** in laboratory.	Yes	Figure legends
In the figure legends: define whether data describe **technical or biological replicates**.	Yes	Figure legends - samples analyzed are individual animals or materials from individual animals and are thus by definition biological replicates

Ethics

Ethics	Information included in the manuscript?	In which section is the information available? (Reagents and Tools Table, Materials and Methods, Figures, Data Availability Section)
Studies involving **human participants**: State details of **authority granting ethics approval** (IRB or equivalent committee(s), provide reference number for approval.	Not Applicable	
Studies involving **human participants**: Include a statement confirming that **informed consent** was obtained from all subjects and that the experiments conformed to the principles set out in the WMA Declaration of Helsinki and the Department of Health and Human Services Belmont Report.	Not Applicable	
Studies involving **human participants:** For publication of **patient photos**, include a statement confirming that consent to publish was obtained.	Not Applicable	
Studies involving experimental **animals**: State details of **authority granting ethics approval** (IRB or equivalent committee(s), provide reference number for approval. Include a statement of compliance with ethical regulations.	Yes	Information is in Materials and Methods.
Studies involving **specimen and field samples:** State if relevant **permits** obtained, provide details of authority approving study; if none were required, explain why.	Not Applicable	

Dual Use Research of Concern (DURC)	Information included in the manuscript?	In which section is the information available? (Reagents and Tools Table, Materials and Methods, Figures, Data Availability Section)
Could your study fall under dual use research restrictions? Please check biosecurity documents and list of **select agents and toxins** (CDC): https://www.selectagents.gov/sat/list.htm	Not Applicable	
If you used a select agent, is the security level of the lab appropriate and reported in the manuscript?	Not Applicable	
If a study is subject to dual use research of concern regulations, is the name of the **authority granting approval and reference number** for the regulatory approval provided in the manuscript?	Not Applicable	

Reporting

The MDAR framework recommends adoption of discipline-specific guidelines, established and endorsed through community initiatives. Journals have their own policy about requiring specific guidelines and recommendations to complement MDAR.

Adherence to community standards	Information included in the manuscript?	In which section is the information available? (Reagents and Tools Table, Materials and Methods, Figures, Data Availability Section)
State if relevant guidelines or checklists (e.g., **ICMJE, MIBBI, ARRIVE, PRISMA**) have been followed or provided.	Not Applicable	
For **tumor marker prognostic studies,** we recommend that you follow the **REMARK** reporting guidelines (see link list at top right). See author guidelines, under 'Reporting Guidelines'. Please confirm you have followed these guidelines.	Not Applicable	
For **phase II and III randomized controlled trials**, please refer to the **CONSORT** flow diagram (see link list at top right) and submit the CONSORT checklist (see link list at top right) with your submission. See author guidelines, under 'Reporting Guidelines'. Please confirm you have submitted this list.	Not Applicable	

Data Availability

Data availability	Information included in the manuscript?	In which section is the information available? (Reagents and Tools Table, Materials and Methods, Figures, Data Availability Section)
Have **primary datasets** been deposited according to the journal's guidelines (see 'Data Deposition' section) and the respective accession numbers provided in the Data Availability Section?	Not Applicable	To our knowledge, none of our data require deposition in external databases. We have gathered datasets to supply to EMBO (microscopy files, flow cytometry files). These are quite large and we need some guidance regarding how to deliver these.
Were **human clinical and genomic datasets** deposited in a public access-controlled repository in accordance to ethical obligations to the patients and to the applicable consent agreement?	Not Applicable	
Are **computational models** that are central and integral to a study available without restrictions in a machine-readable form? Were the relevant accession numbers or links provided?	Not Applicable	
If publicly available data were reused, provide the respective **data citations in the reference list**.	Not Applicable	
